# Supplementary material for: Variation in surface protein expression leads to heterogeneous Trypanosoma cruzi populations during host cell infection
Source: Nat Commun. 2025 Nov 12;16:9949. doi: 10.1038/s41467-025-64900-2 (PMC12612098; doi:10.1038/s41467-025-64900-2)
Supplement: Supplementary file 1 — Supplementary Information [file 41467_2025_64900_MOESM1_ESM.pdf]

Supplementary Figures

Variation in surface protein expression leads to heterogeneous *Trypanosoma cruzi* populations during host cell infection

Lissa Cruz-Saavedra[1], Mira Loock[1], Luiza Berenguer Antunes[1], Igor Cestari[1,2,*]

[1]Institute of Parasitology, McGill University, Sainte-Anne-de-Bellevue, QC H9X 3V9, Canada

[2]Division of Clinical and Translational Research, Department of Medicine, McGill University, Montreal, QC, H4A 3J1, Canada

* Correspondence: igor.cestari@mcgill.ca

Supplementary Figure 1. Pore-C interaction analysis. A) Heatmap of pore-c interaction matrix. The matrix was balanced using the Knight and Ruiz method using HicExplorer tools. B) Heatmap of pore-c interaction matrix after observed/expected normalization to remove distance bias. C) Total, cis and trans interactions obtained by pore-c. Analysis was performed using Pairtools. D) Fraction of Cis interactions per distance from data in C. For these analyses, the pore-c fastq data were mapped to the *T. cruzi* Sylvio X10 strain genome (this work), DNA contacts were calculated using Pairtools and converted to a matrix using Cooler. Matrix normalization and visualization were generated using HicExplorer. One Pore-C experiment was performed.

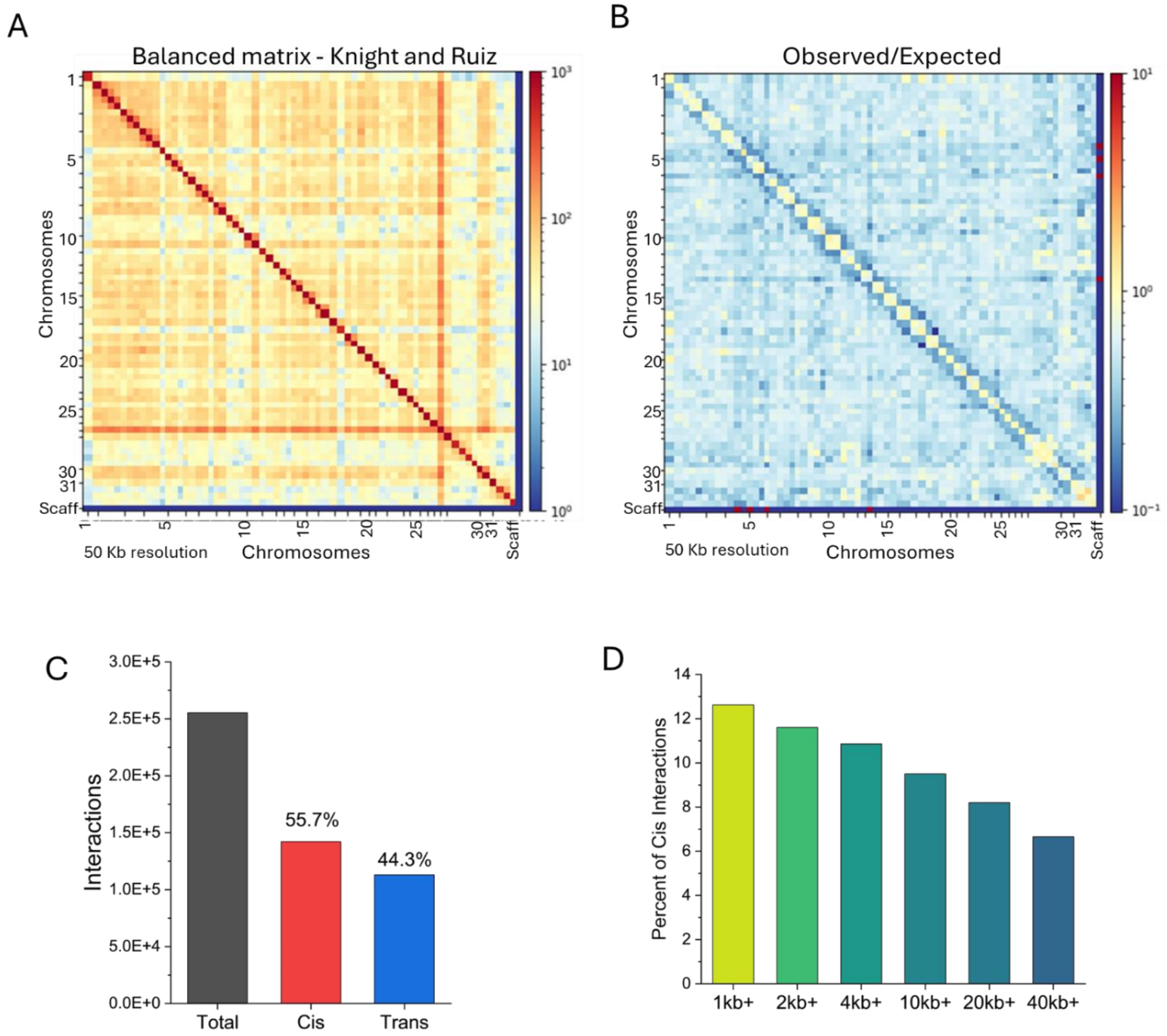

Supplementary Figure 2. Chromosomal depth and repeat content in *T. cruzi* TcI strain Sylvio X10. A) The circos plot displays chromosomal depth calculated in 10,000 bp windows to PacBio (397x) and nanopore (70x) data mapped against the *T. cruzi* Sylvio X10 2025 assembled genome. Chromosome 30 shows an increase in depth, consistent with a trisomy structure, while chromosome 1 exhibits decreased depth. Some chromosomes, such as chromosomes 10, show evidence of segmental aneuploidy, indicated by a decrease in depth in the initial regions of the chromosome. B) The percentage of repeat content is shown across chromosomes, with high repeat content (>70%) observed on chromosomes 9, 12, 29 and 30 (indicated with asterisks), which are enriched in multigene family (MGF) sequences.

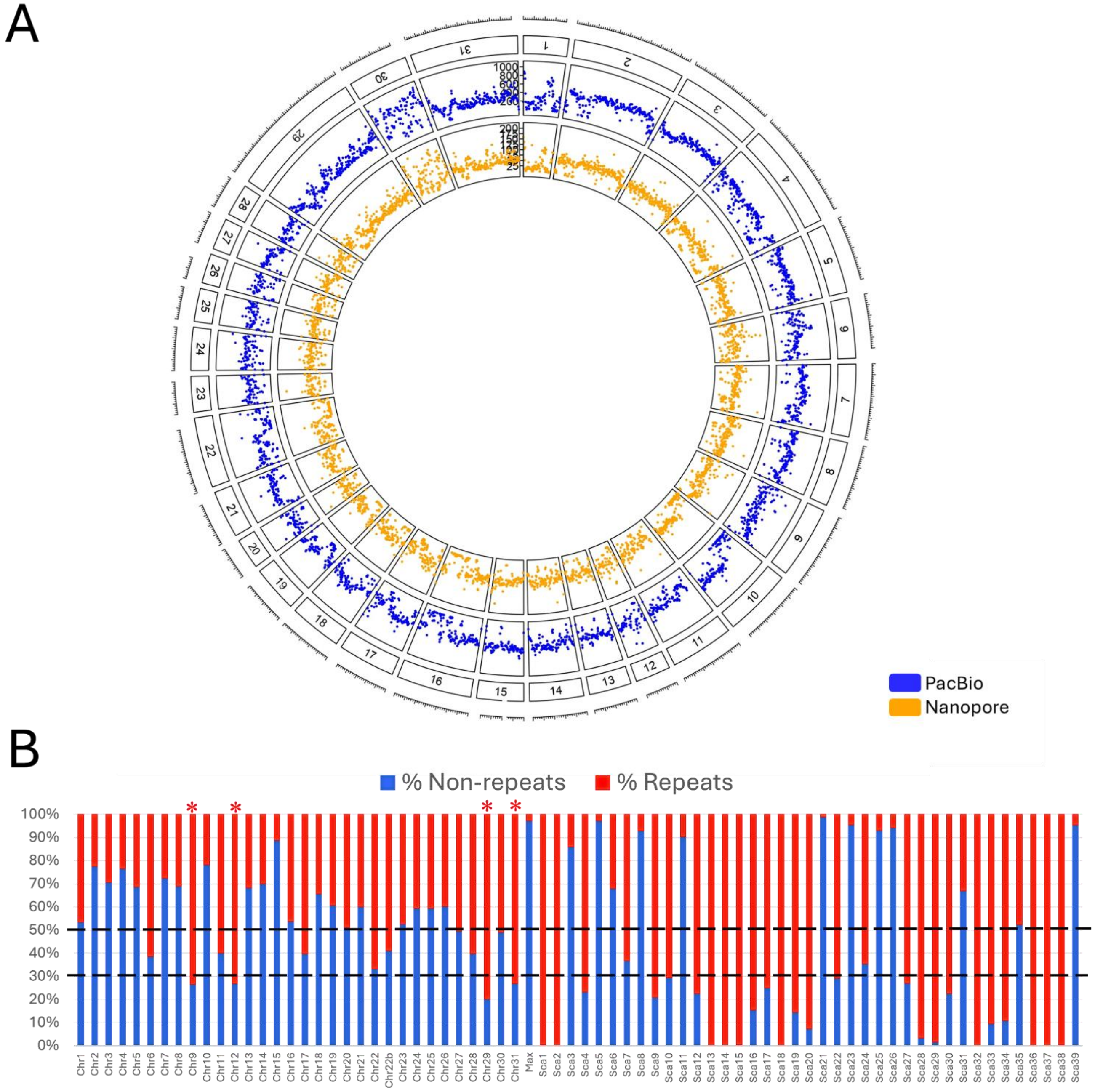

Supplementary Figure 3. Mitochondrial genome structure. A) The circos plot displays a map of the assembled *T. cruzi* mitochondrial maxicircles, highlighting the locations of unedited genes, long repeat regions, and short repeat regions.

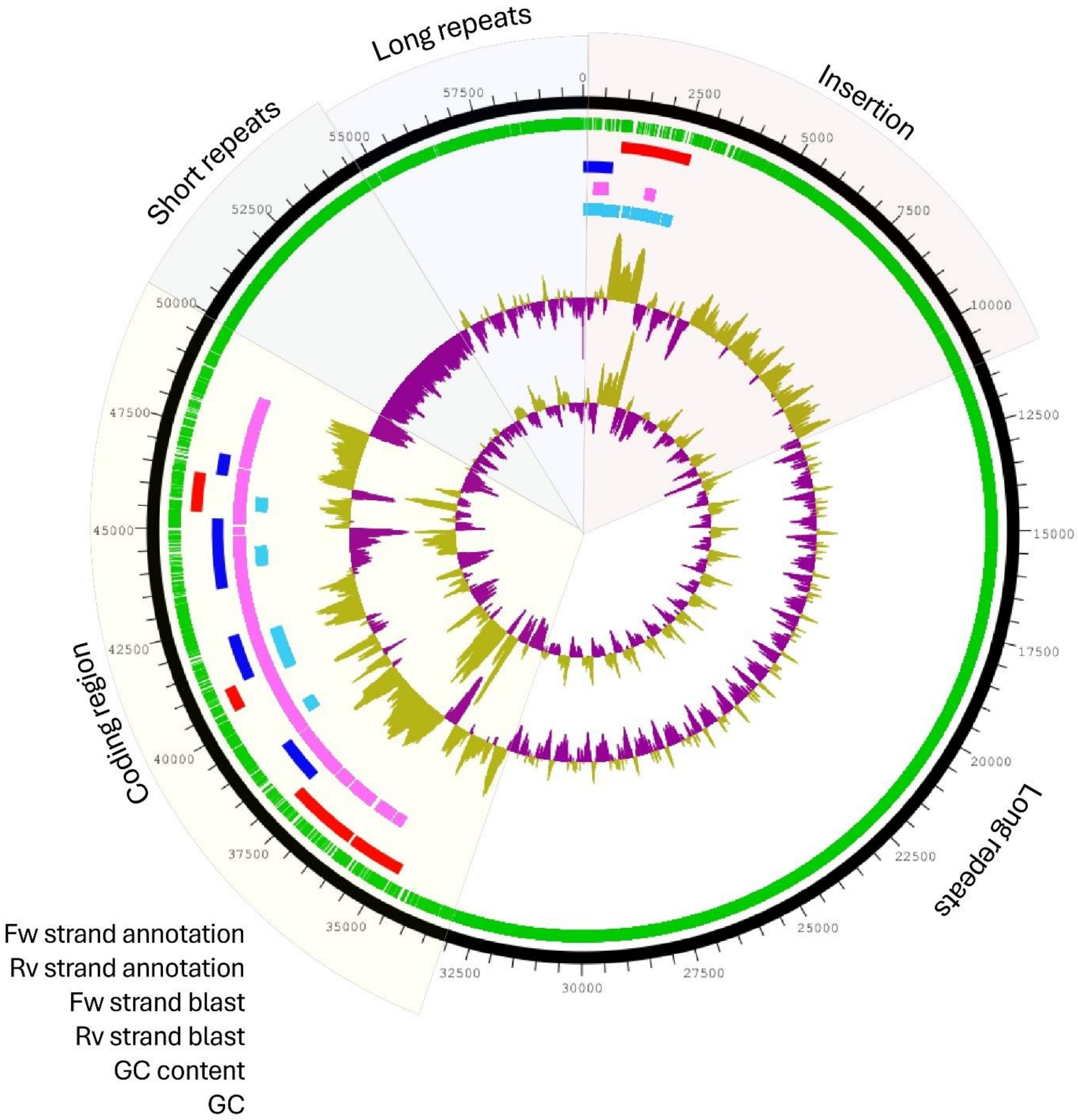

Supplementary Figure 4. Synteny analysis of *T. cruzi* scaffolds and chromosomes. Comparison of assembled short-length scaffolds (<68 kb) to chromosomes. The chromosomes are ordered from the highest to the lowest number of scaffold matches. Chromosome 16 shows synteny with eight scaffolds, chromosome 30 with six scaffolds, and chromosome 26 with five scaffolds. All 39 scaffolds display synteny with some chromosomes.

Chr3-11: 1 scaffolds synteny

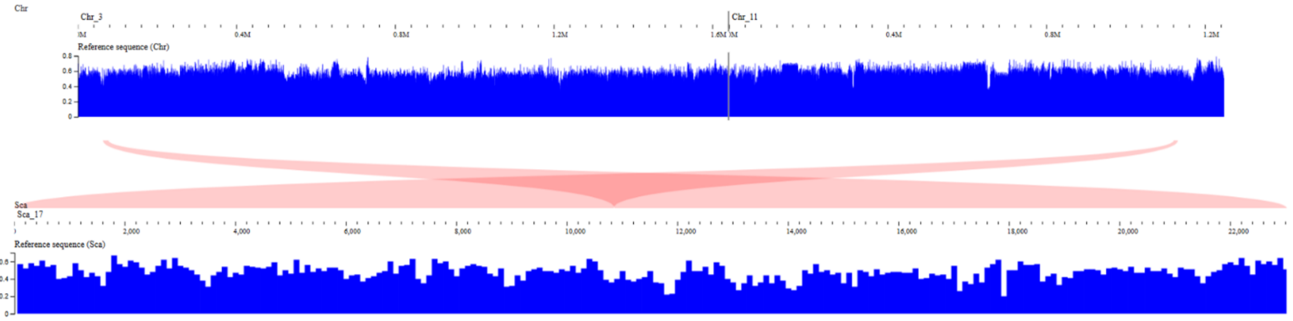

Chr9: 1 scaffolds synteny

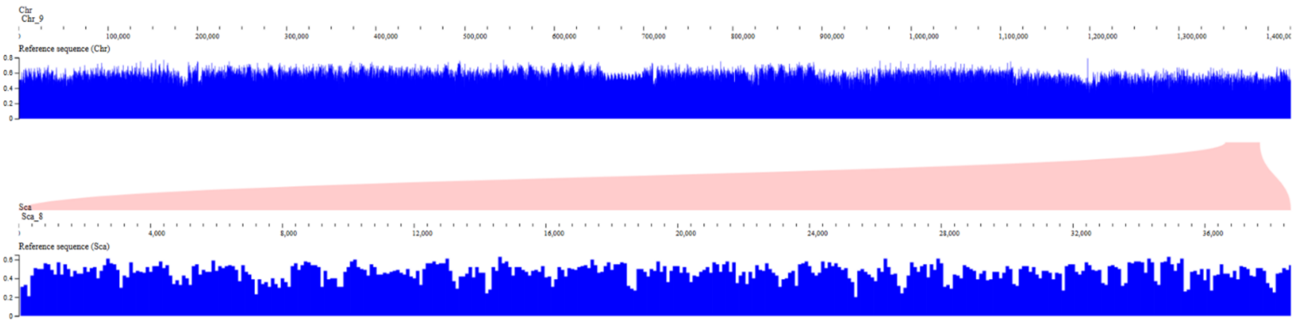

Chr25: 1 scaffold synteny

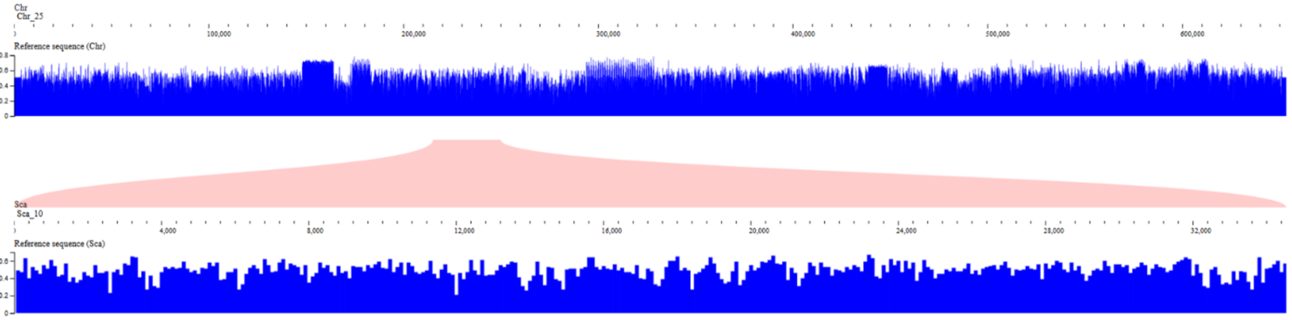

Chr15: 1 scaffold synteny

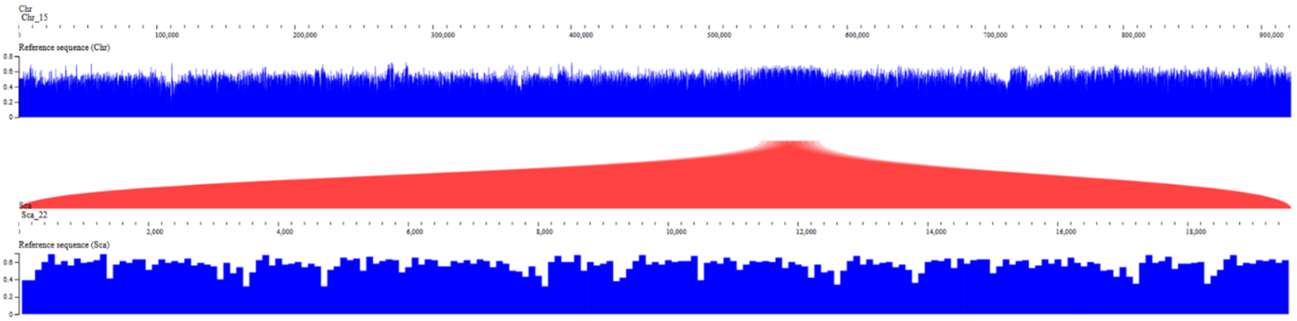

Chr12: 2 scaffolds synteny

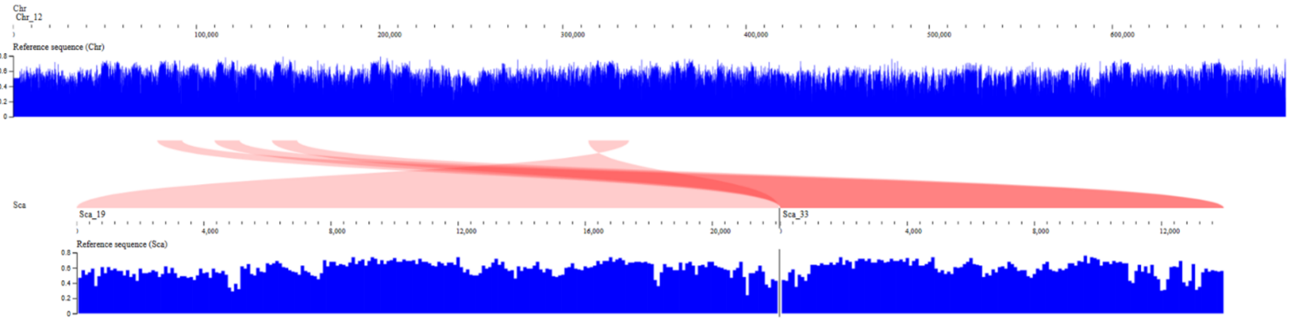

Chr10: 2 scaffolds synteny

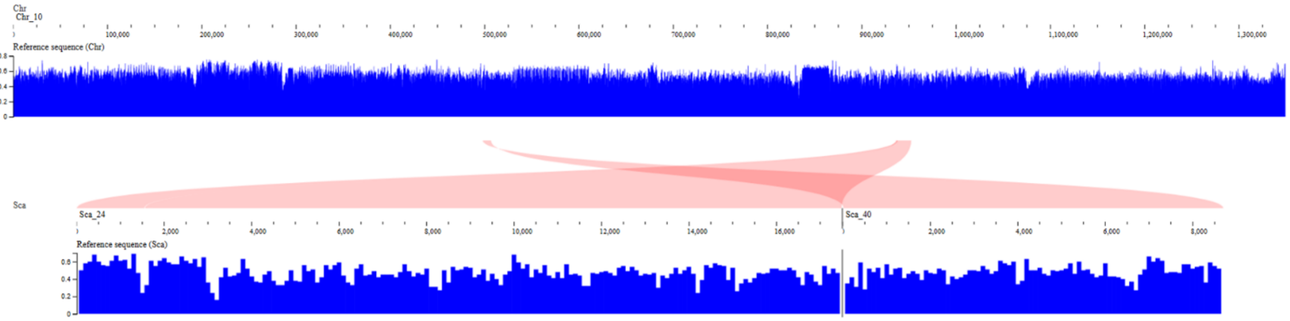

Chr8: 2 scaffolds synteny

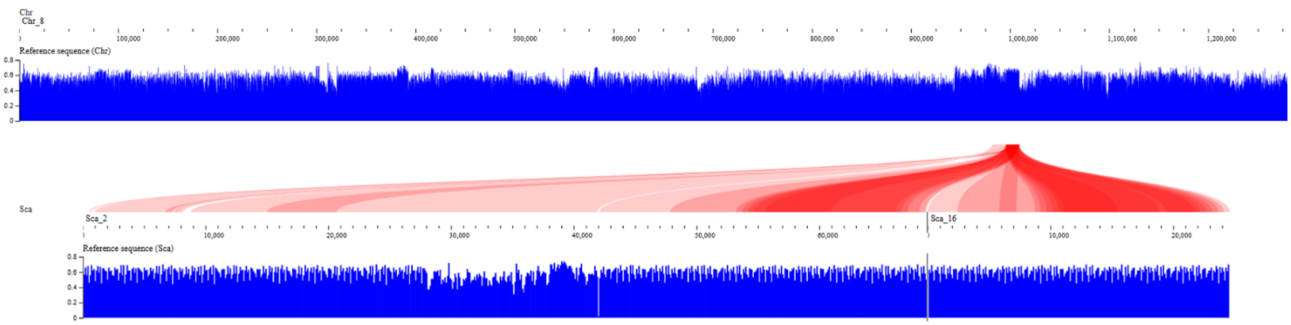

Chr4: 2 scaffolds synteny

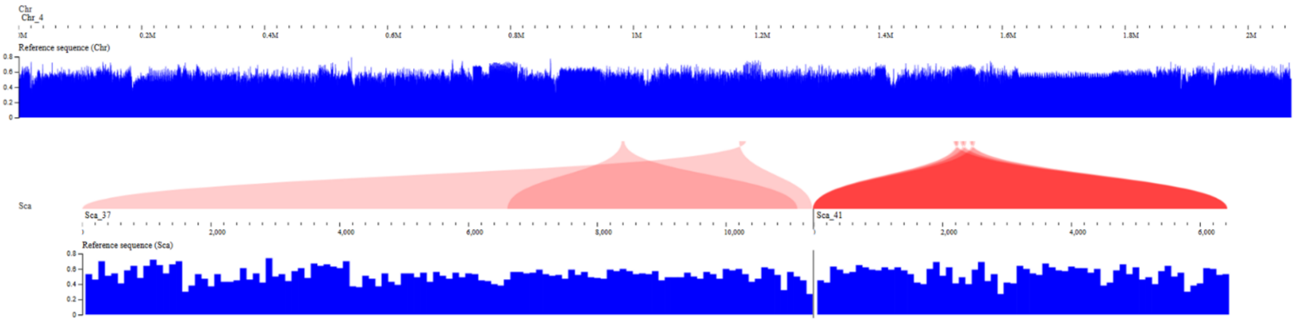

Chr2: 2 scaffolds synteny

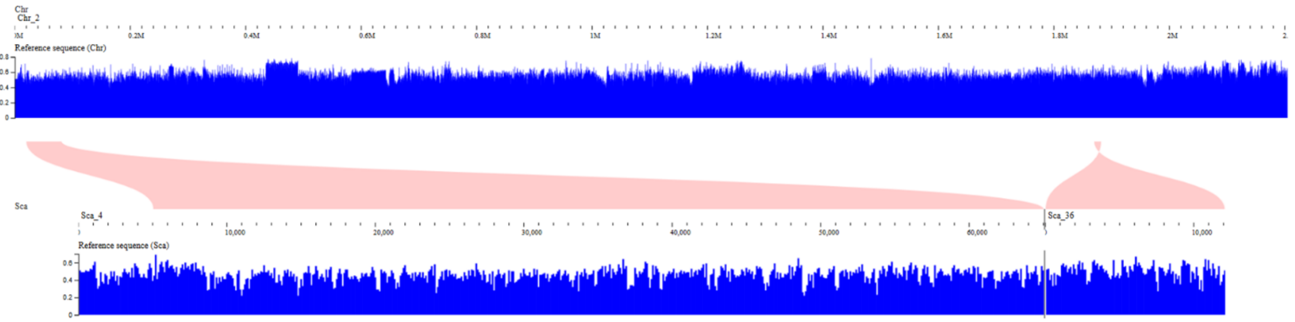

Chr22: 2 scaffolds synteny

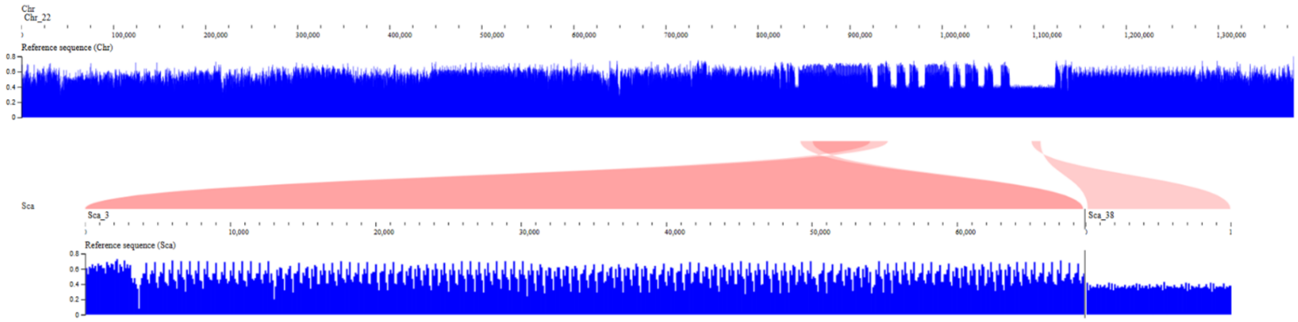

Chr_7: 3 scaffolds synteny

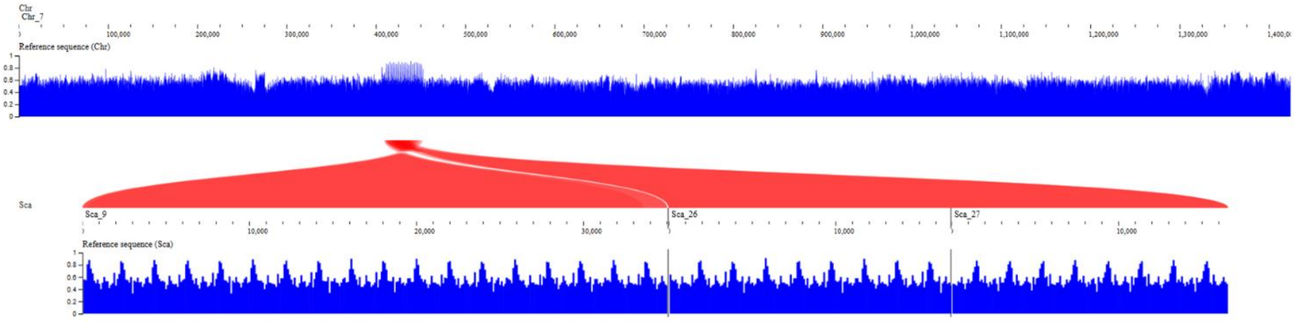

Chr_26: 4 scaffolds synteny

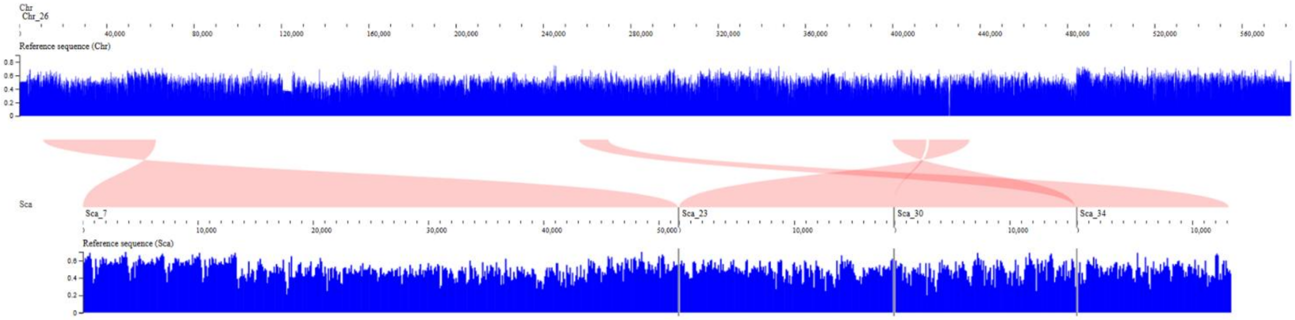

Chr_30: 6 scaffolds synteny

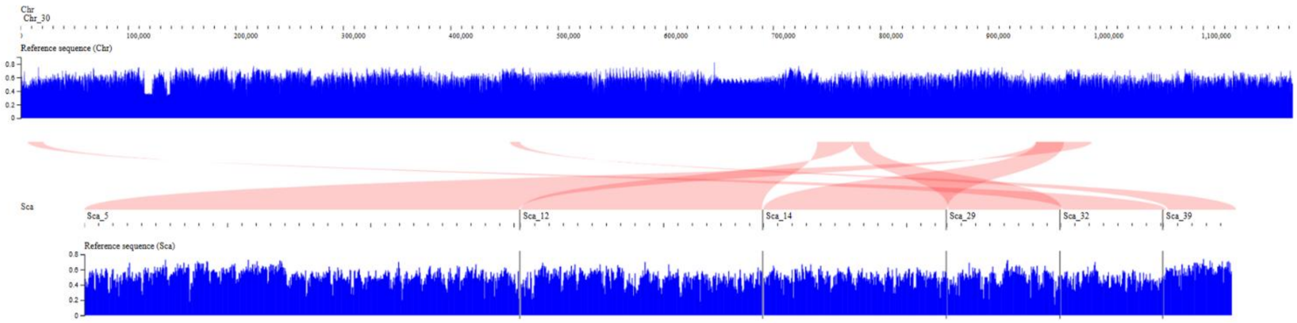

Chr_16: 8 scaffolds synteny

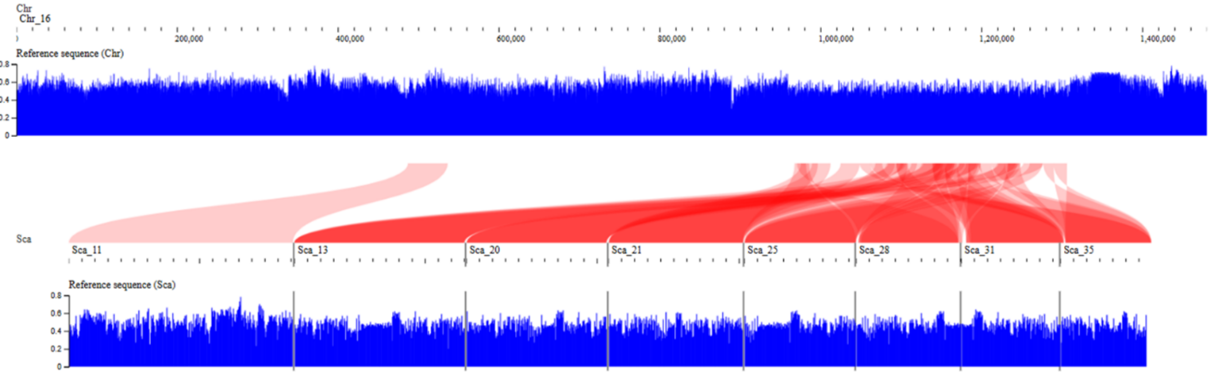

Chr7-28: 1 scaffolds synteny

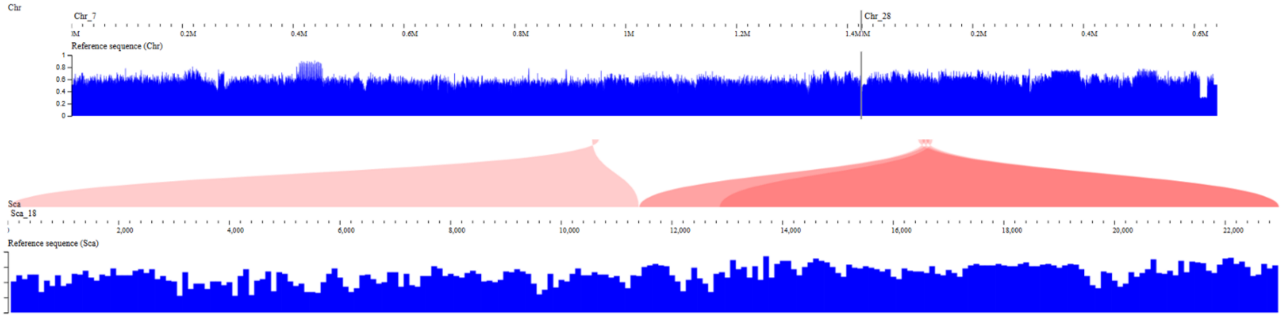

Supplementary Figure 5. Mapping quality statistics using the *T. cruzi* Sylvio X10 genome assemblies from 2018 (Talavera-Lopez C, et al. 2021) and 2025 (this work). A) PacBio HiFi and Nanopore reads were mapped against the *T. cruzi* Sylvio X10 genome assemblies from 2018 and 2025 using Minimap2. In blue, total mapped reads; in orange, primary mapped reads. The 2025 assembly shows higher overall mapping, indicating improved genome completeness and accuracy. B) RNA-seq data generated from Illumina HiSeq and MiSeq platforms were mapped to both the 2018 (dark blue) and 2025 (light blue) genomes using STAR. The 2025 genome markedly improves mapping performance, increasing unique mapping percentages and reducing unmapped reads. This reflects better genome continuity and annotation. A slight increase in multi-mapping events is consistent with improved resolution of repetitive regions, particularly multigene families. C) Distribution of RNA-seq reads from HiSeq (red) and MiSeq (blue) platforms mapped per chromosome/scaffold of the 2025 genome. Most reads mapped to chromosomes rather than scaffolds, confirming improved assembly structure and chromosomal representation.

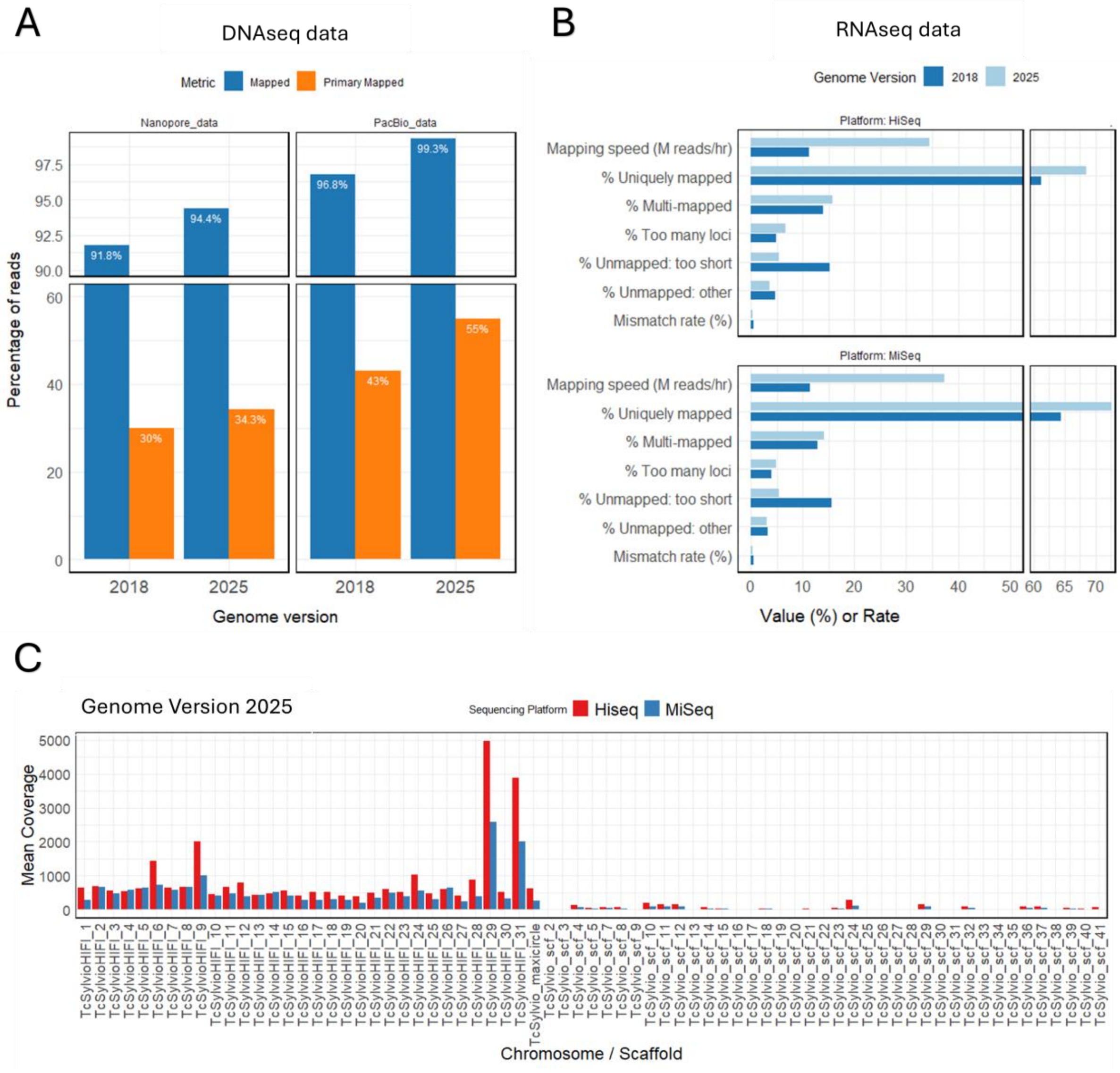

Supplementary Figure 6. Synteny between of *T. cruzi* genomes. The graph compares synteny of the genomes of *T. cruzi* strain X10 (this work) and the genomes of the strains DM25, Brazil A4, Sylvio X10-2018 assembly, and CL-Brener Esmeraldo-like.

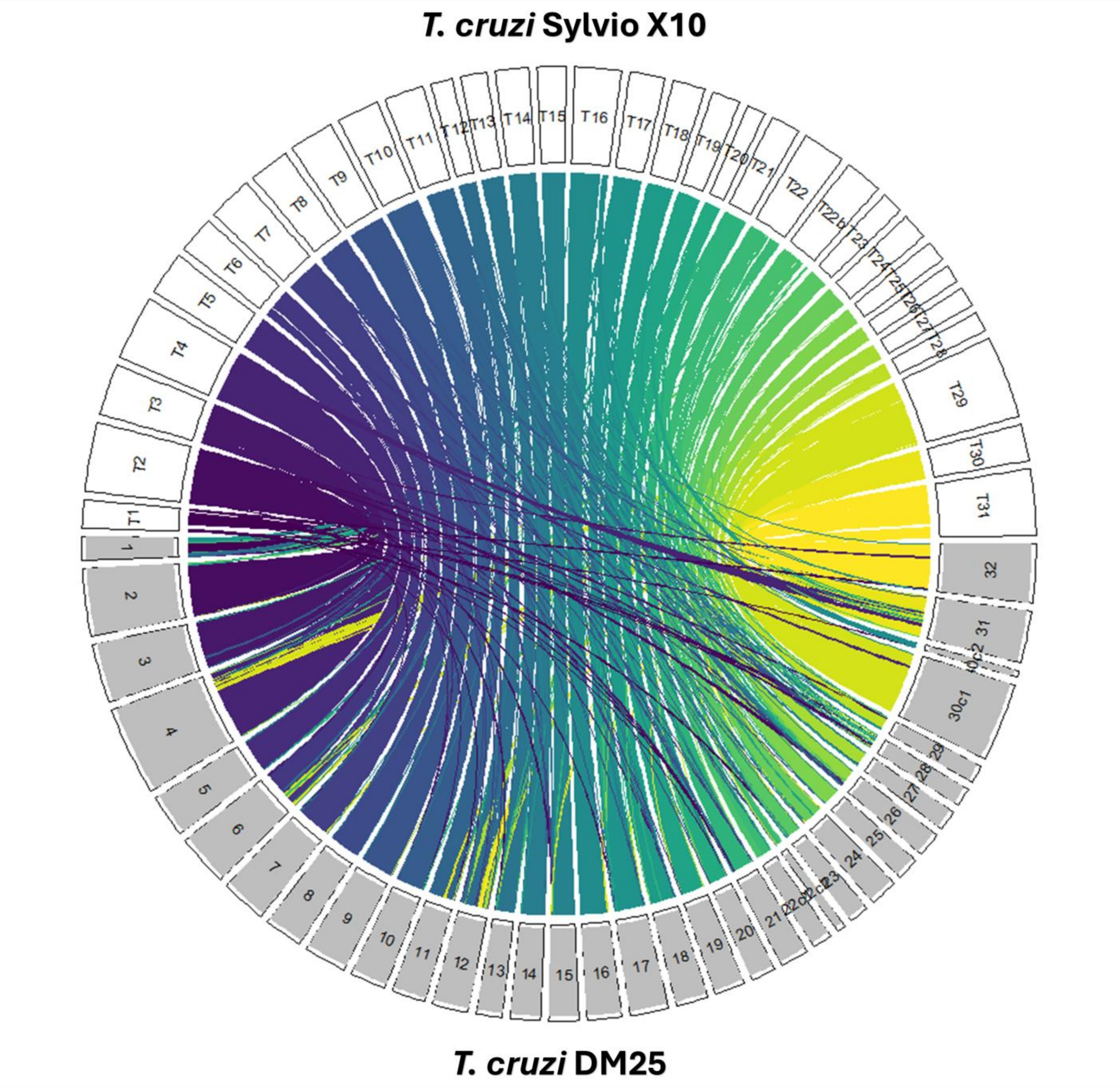

T. cruzi Sylvio X10

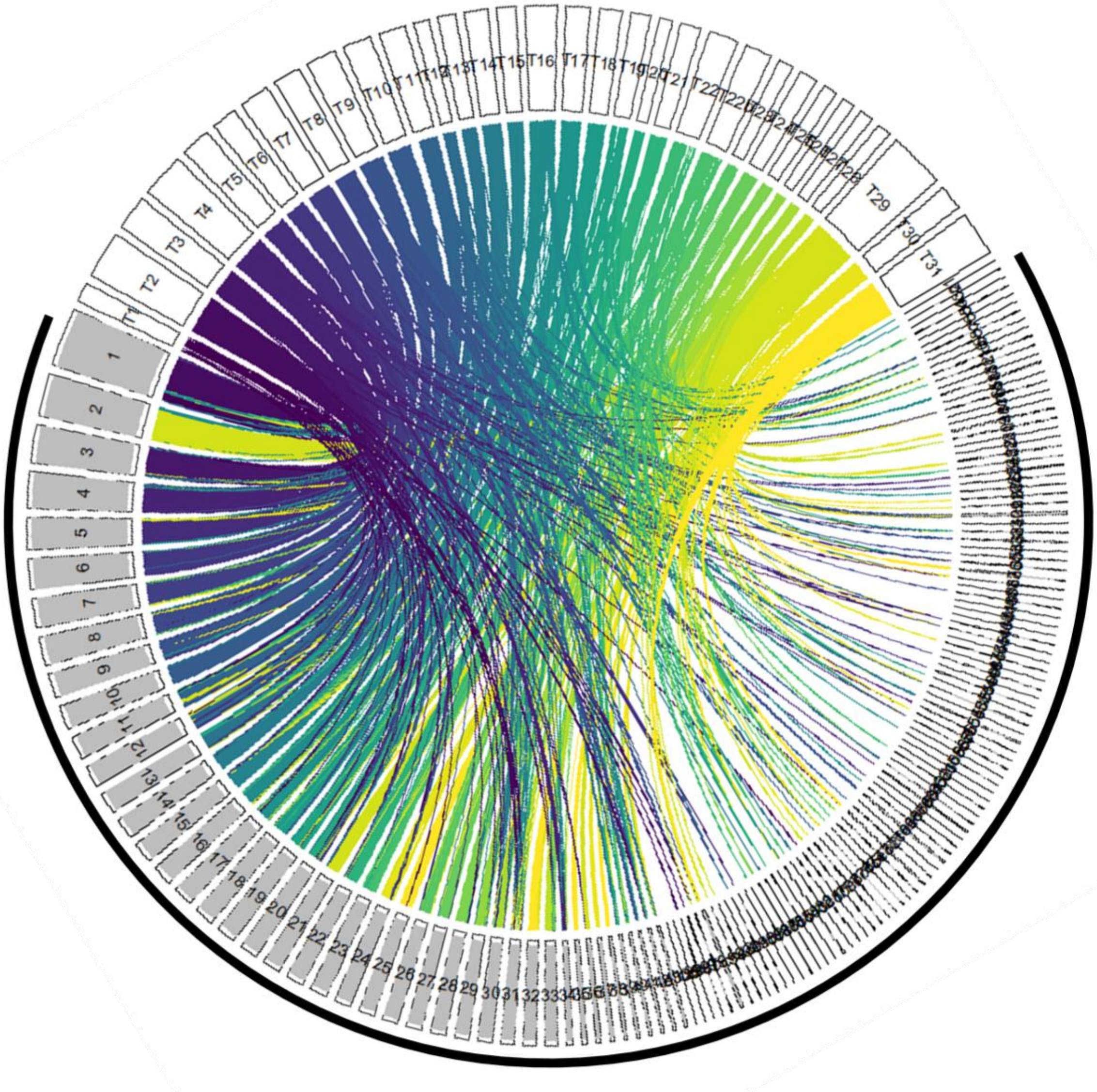

T. cruzi Brazil A4

***T. cruzi* Sylvio X10**

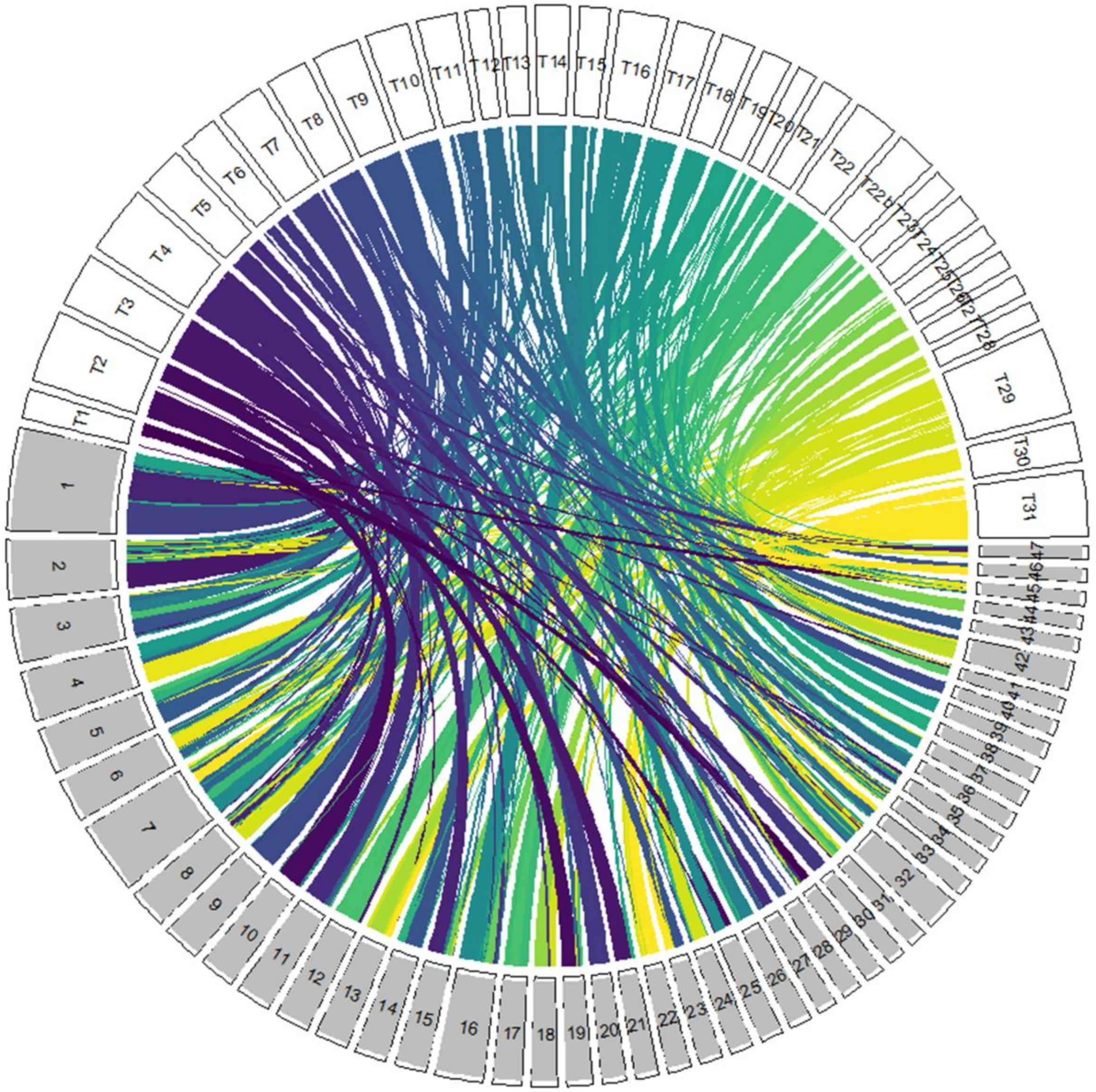

***T. cruzi* Sylvio X10 - 2018**

***T. cruzi* Sylvio X10**

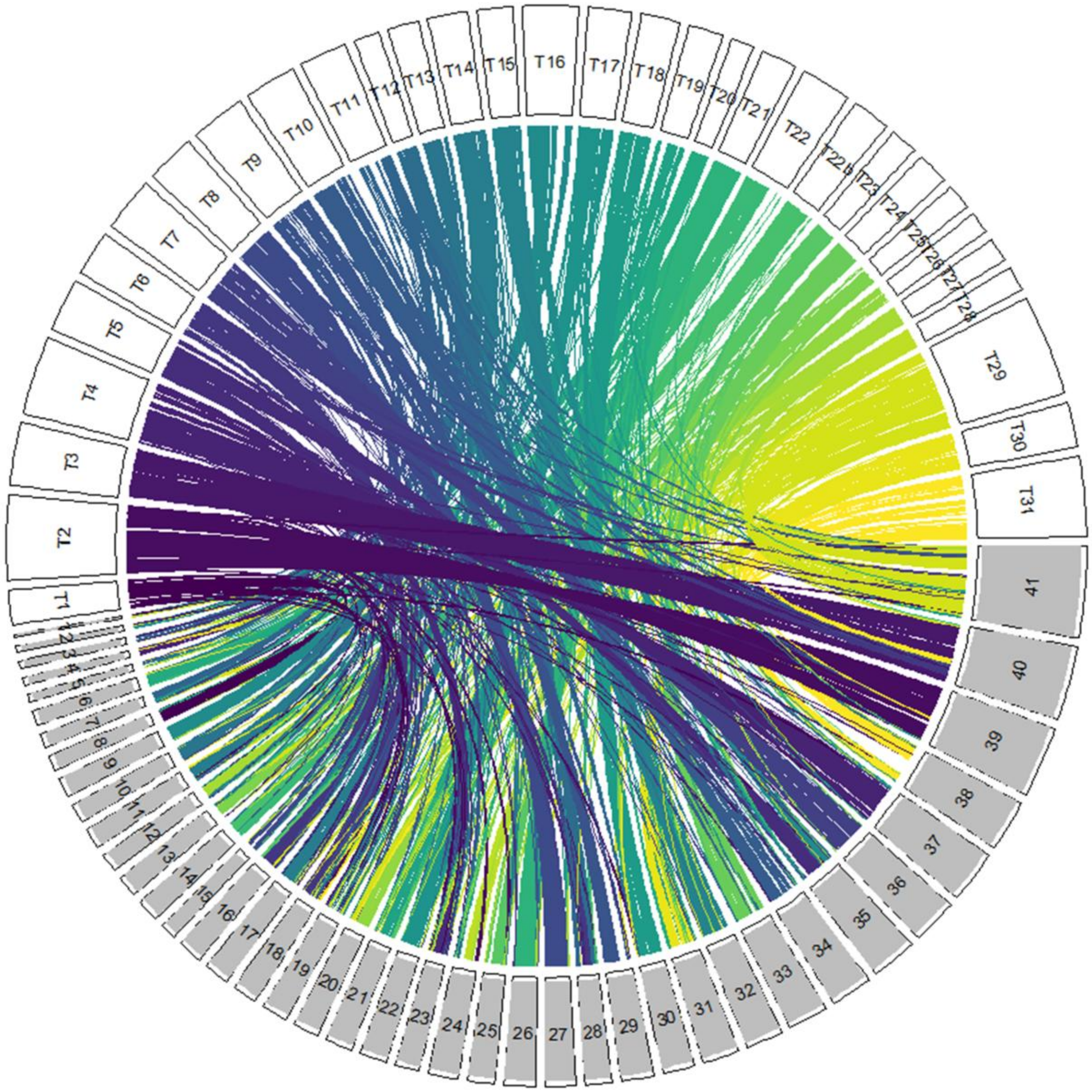

***T. cruzi* Sylvio Cl-Brenner Esmeraldo like**

Supplementary Figure 7. Gene counts and GC content of subtelomeric and non-subtelomeric RHS and DGF-1 genes. A) Total counts of RHS and DGF-1 genes across the *T. cruzi* Sylvio X10 genome (this work), separated into subtelomeric (blue) and non-subtelomeric (green) regions. B) Percent of GC content (%) of RHS and DGF-1 genes located in subtelomeric (blue) versus non-subtelomeric (green) regions. The red line indicates the 50% threshold. The subtelomeric regions were defined as a 50 Kb segment from the chromosome end, excluding telomeric repeats. The Violin plots illustrate the kernel density distribution of GC content from subtelomeric regions or core regions. The horizontal line within the box indicates the median; box limits, the 25th and 75th percentiles; whiskers, the minima and maxima within 1.5x the interquartile range. Dots represent individual measured values. The red dotted line across the graph indicates 50% GC content.

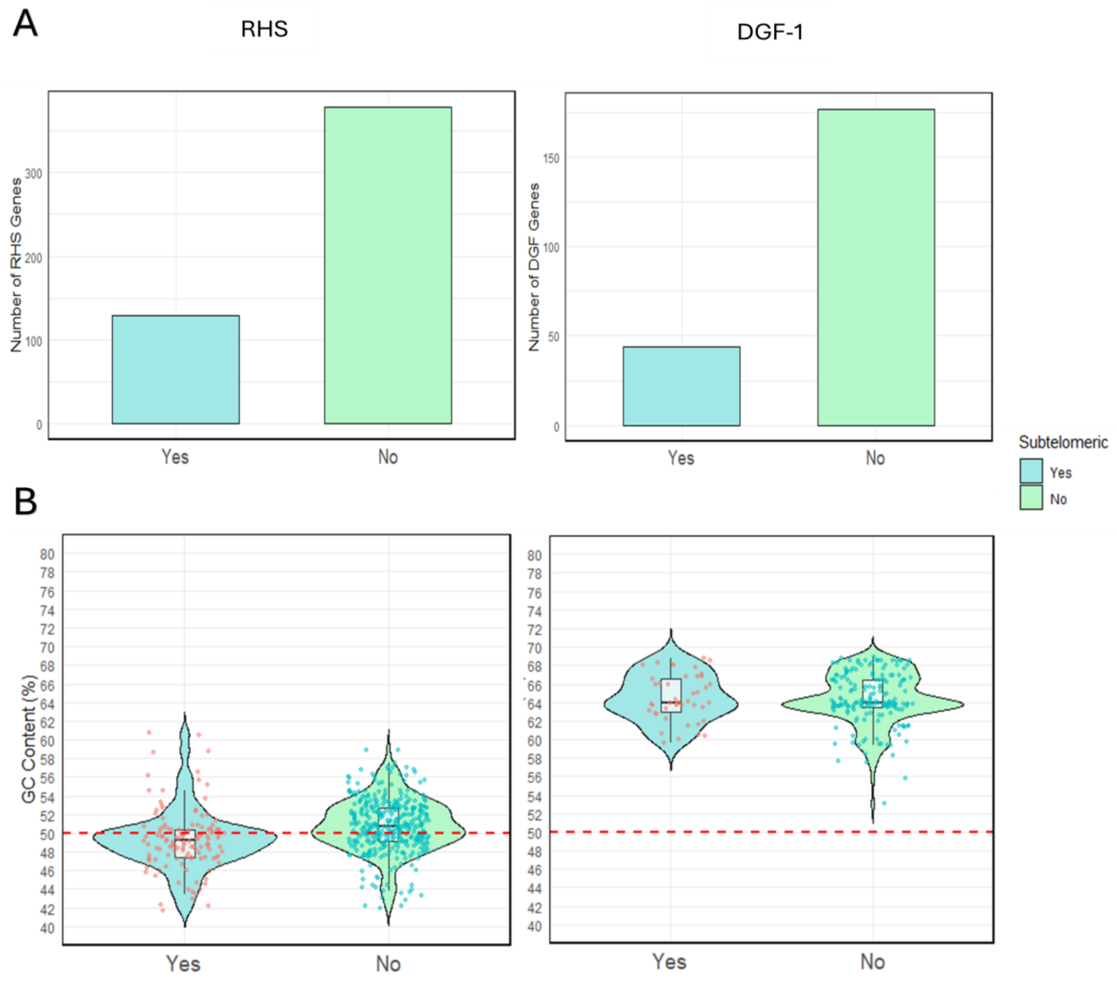

Supplementary Figure 8. Differentially expressed proteins in the *T. cruzi* life cycle. A) Comparison between protein expression changes in non-replicative and replicative stages. MT vs AM, MT vs EP, CT vs AM, and CT vs EP. The top 10 differentially expressed proteins are listed. B) Comparison between CT vs MT. C) Comparison between EP vs AP. Blue bars indicate the top 10 down-regulated proteins, while red bars represent the top 10 up-regulated proteins (Life cycle image created with BioRender.com). Data is based on four biological replicates for each life stage. Diagrams created in BioRender. Cestari, I. (2025) https://BioRender.com/1arv0dd.

A

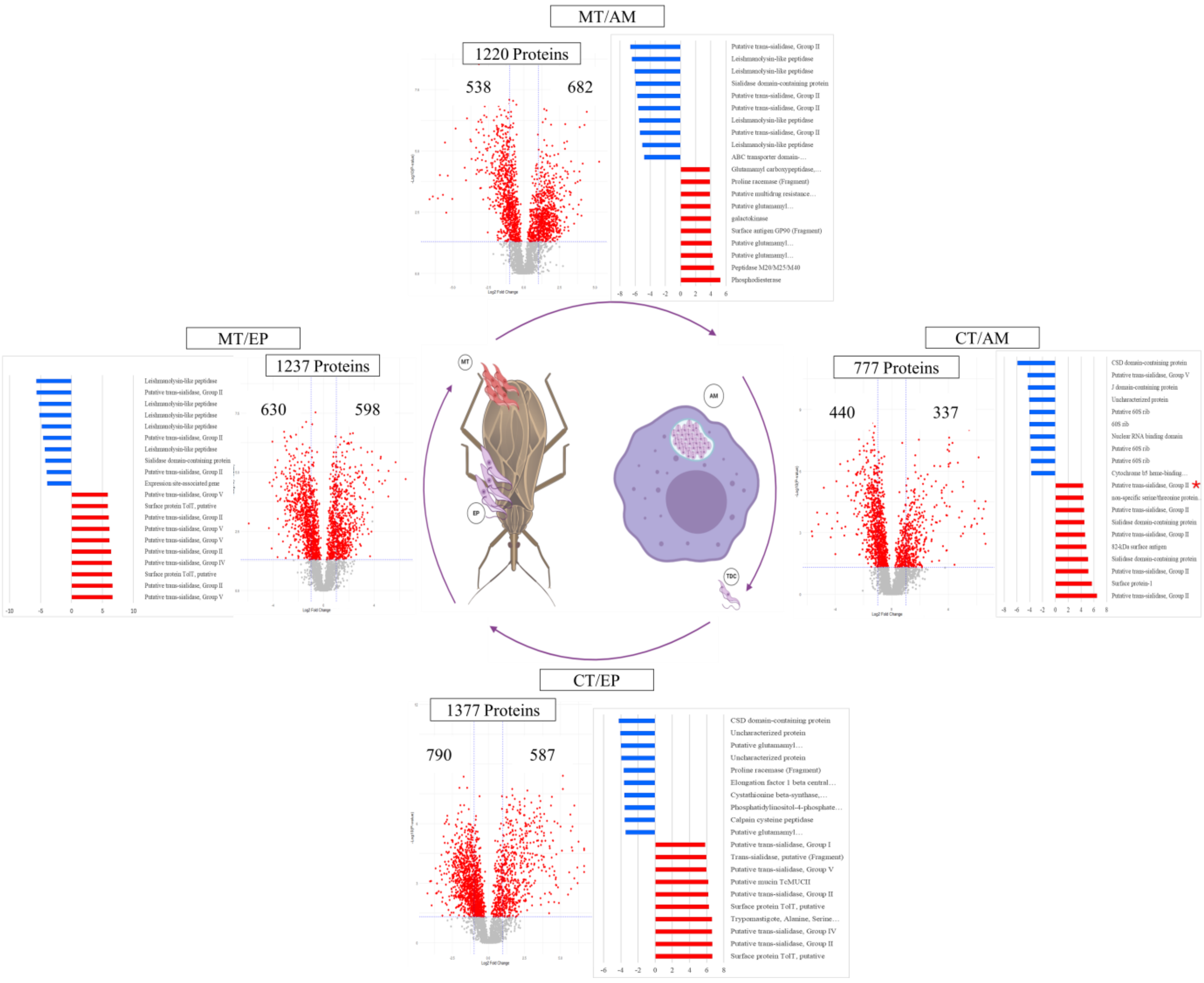

B

Trypomastigotes Infective stages

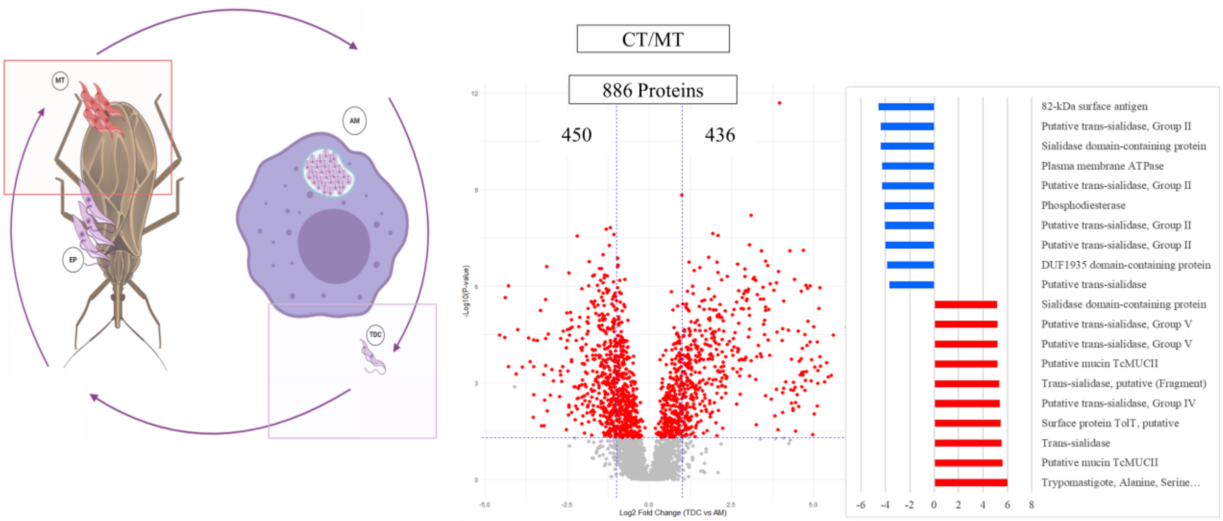

C

Replicative stages

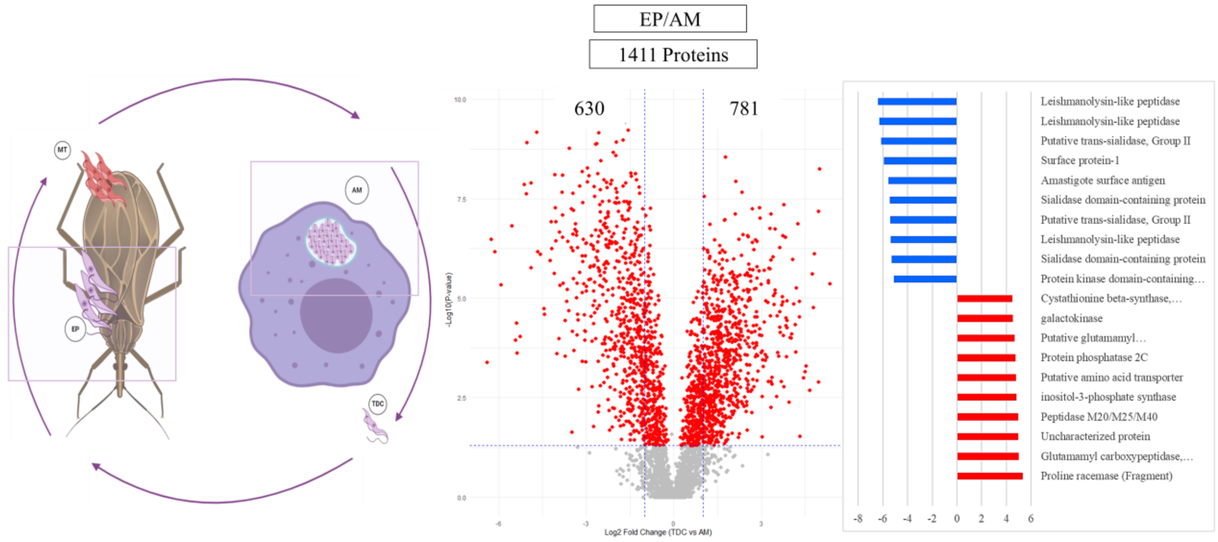

Supplementary Figure 9. Protein expression pattern of MGFs across *T. cruzi* life stages. Cumulative abundance of all dispersed gene family 1 (DGF-1), GP63, mucin-associated surface protein (MASP), mucin, retrotransposon hotspot protein (RHS), and trans-sialidases. Trans-sialidases (TS) are organized into their respective groups I to VIII and the unclassified group. TcMUCII is a mucin group. The abundance of all proteins within a group was summed to represent the cumulative abundance. Data is based on four biological replicates for each life stage.

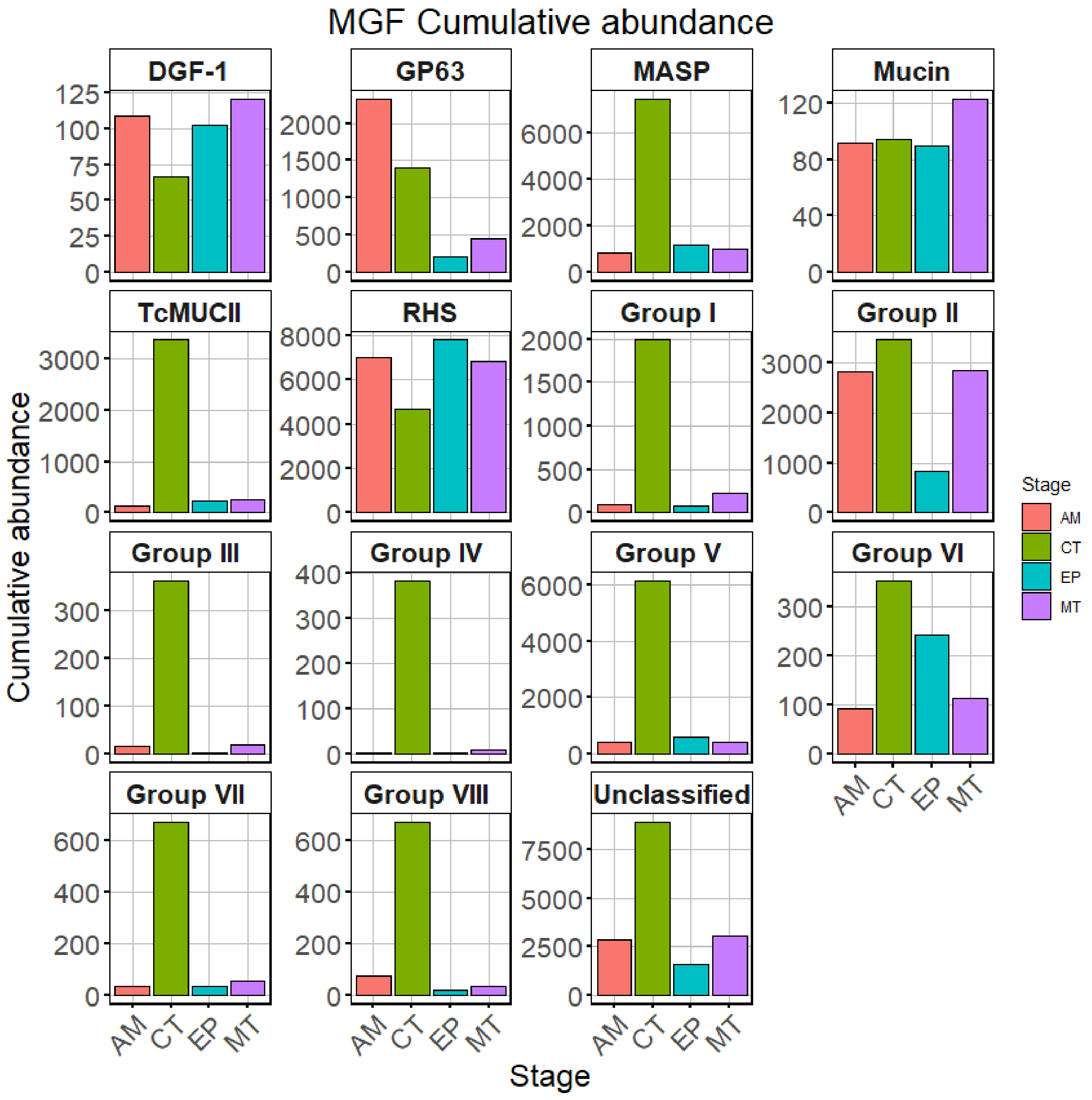

Supplementary Figure 10. Distribution and expression of MGF and core (Other Genes) proteins in all chromosomes across *T. cruzi* life stages. The X-axis represents chromosome length in base pairs, while the Y-axis shows the log_2 protein abundance, calculated as the mean of four biological replicates. The top plot corresponds to MGF and the bottom plot to core genes. Chromosomes enriched in multigene families (MGFs) —specifically chromosomes 6, 9, 29, and 31 — show increased MGF expression in CTs compared to other life cycle stages, as well as compared to non-MGF proteins.

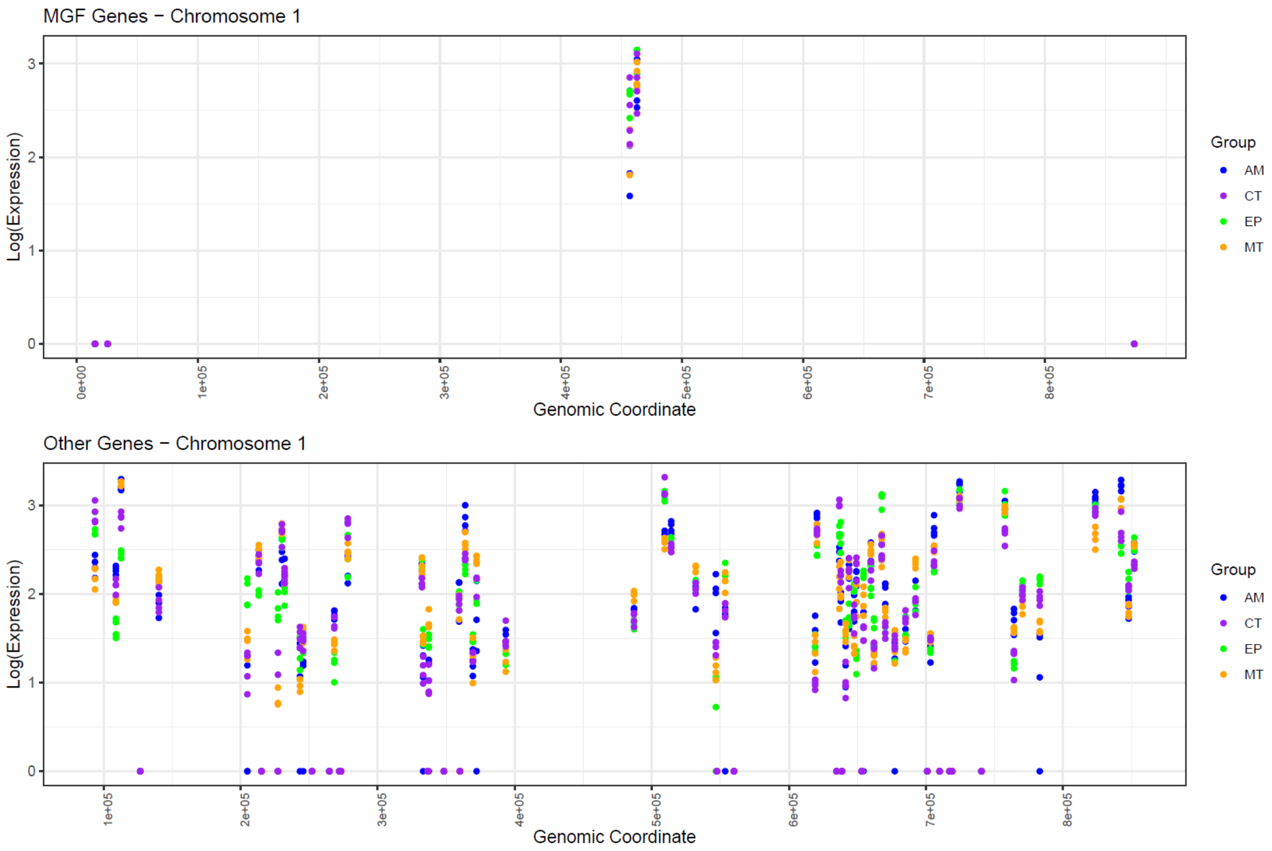

MGF Genes – Chromosome 2

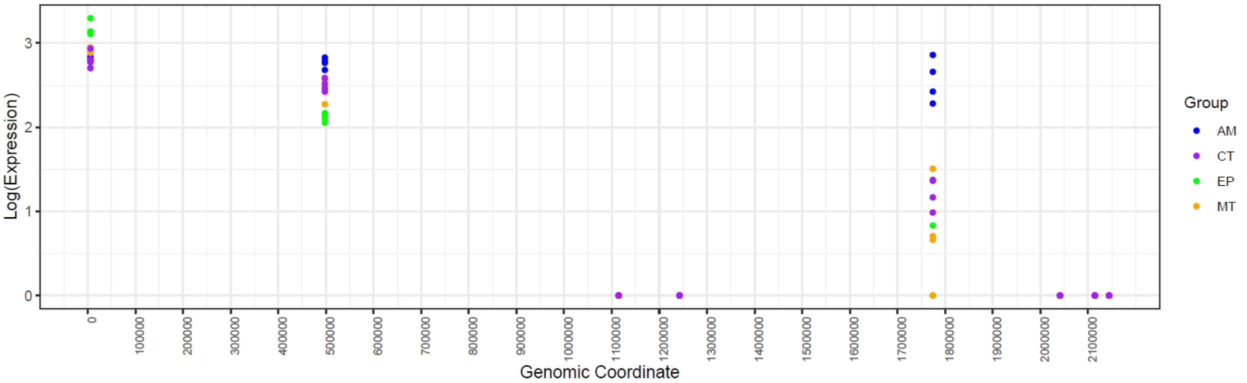

Other Genes – Chromosome 2

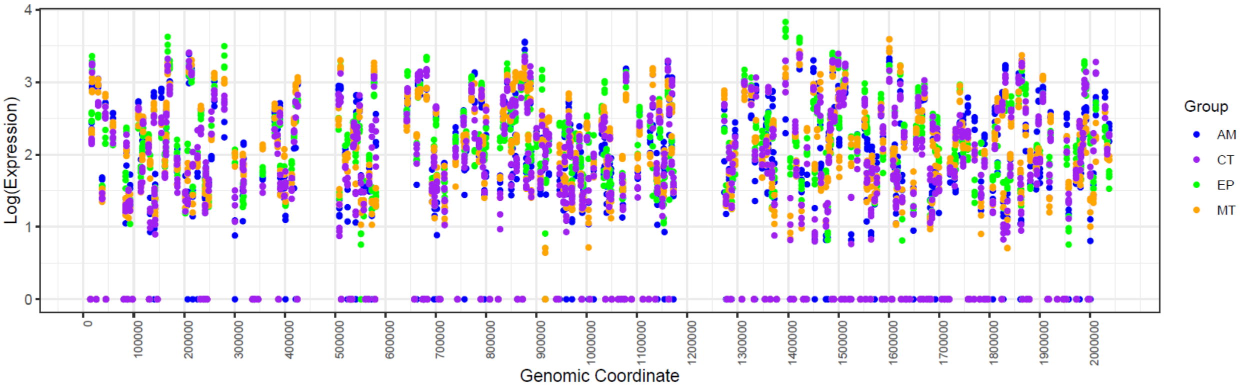

MGF Genes – Chromosome 3

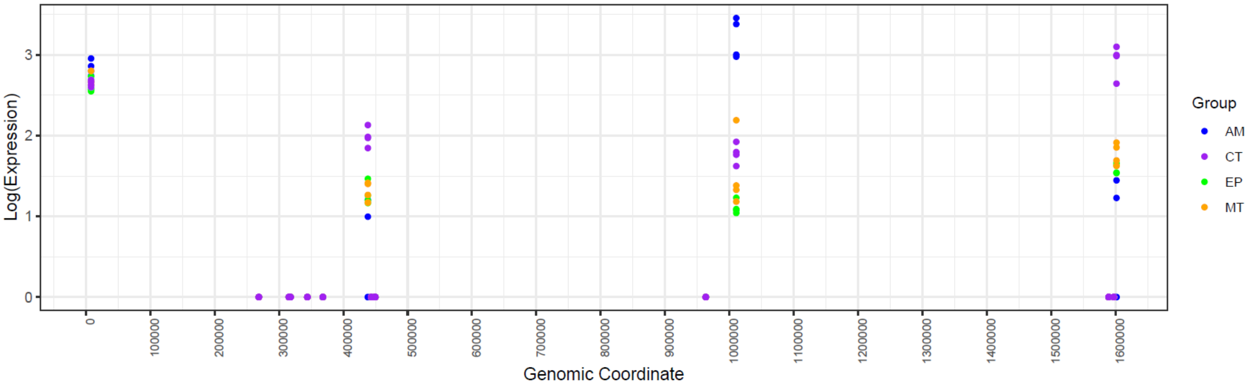

Other Genes – Chromosome 3

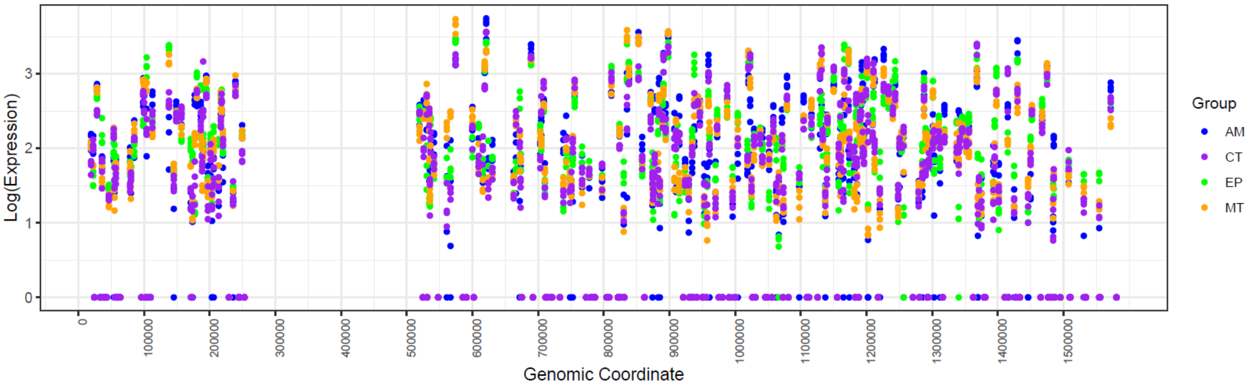

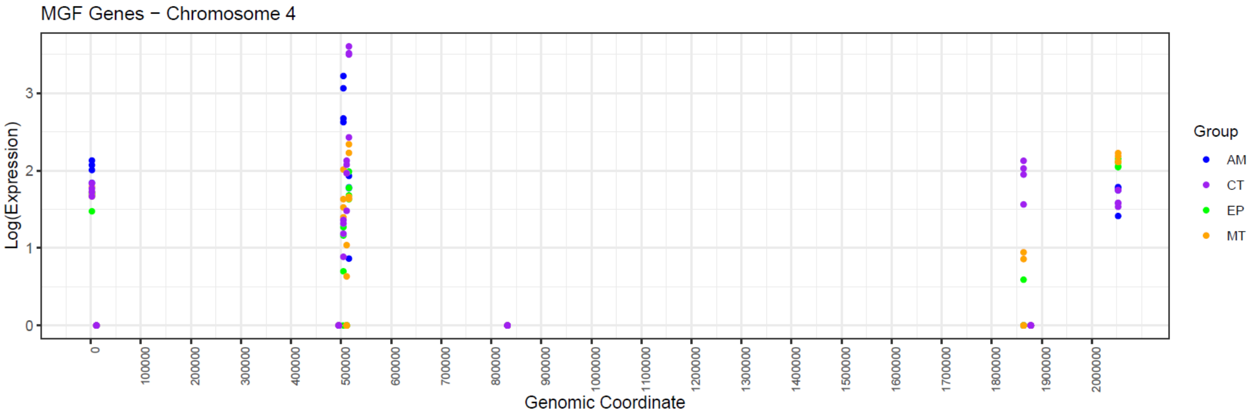
MGF Genes − Chromosome 4
Log(Expression)
Genomic Coordinate
Group
AM
CT
EP
MT

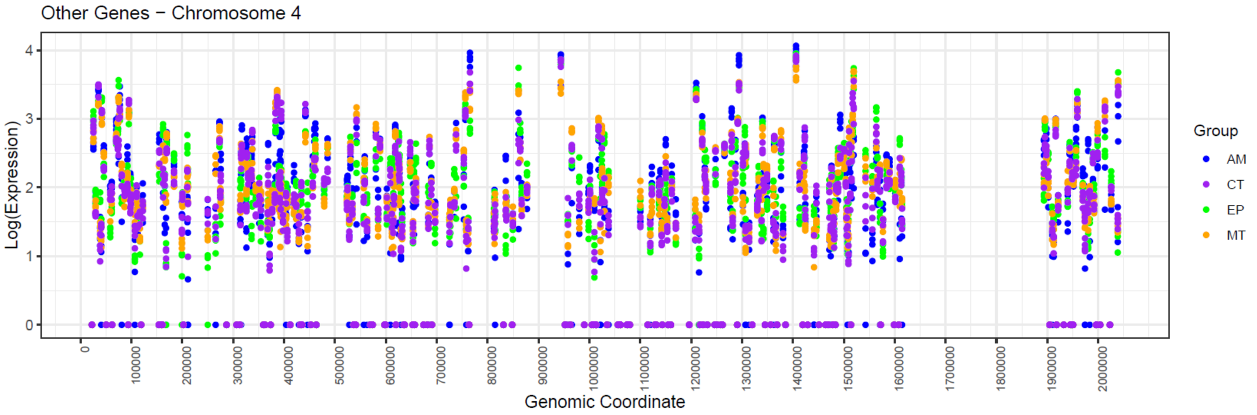
Other Genes − Chromosome 4
Log(Expression)
Genomic Coordinate
Group
AM
CT
EP
MT

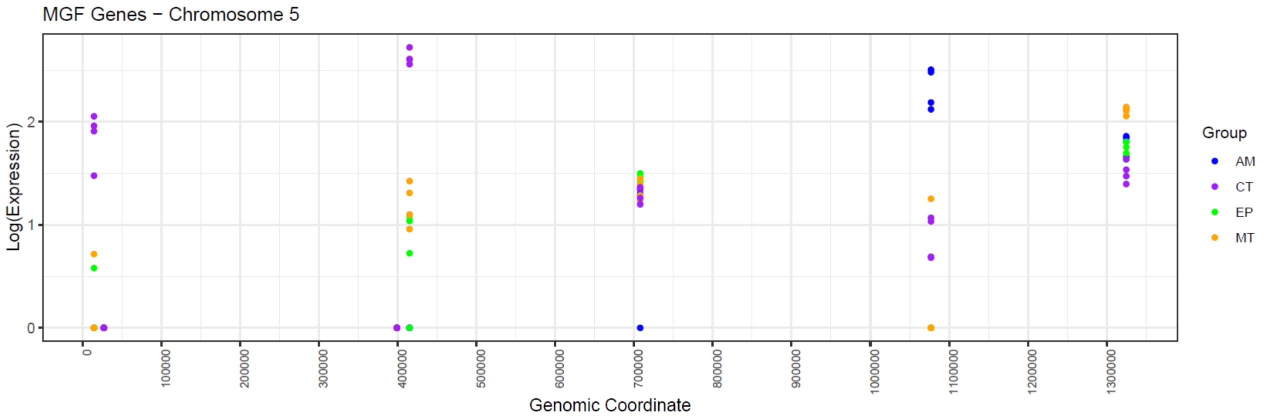
MGF Genes − Chromosome 5
Log(Expression)
Genomic Coordinate
Group
AM
CT
EP
MT

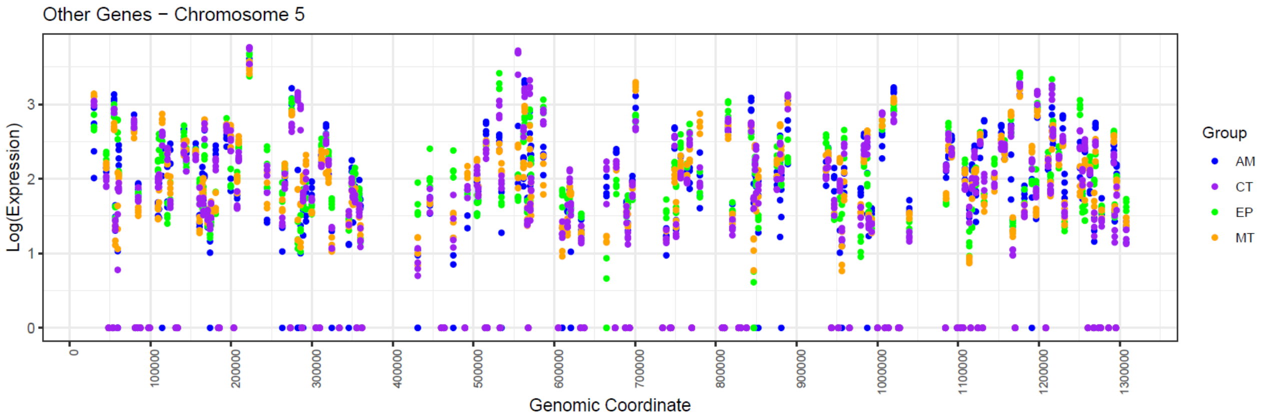
Other Genes − Chromosome 5
Log(Expression)
Genomic Coordinate
Group
AM
CT
EP
MT

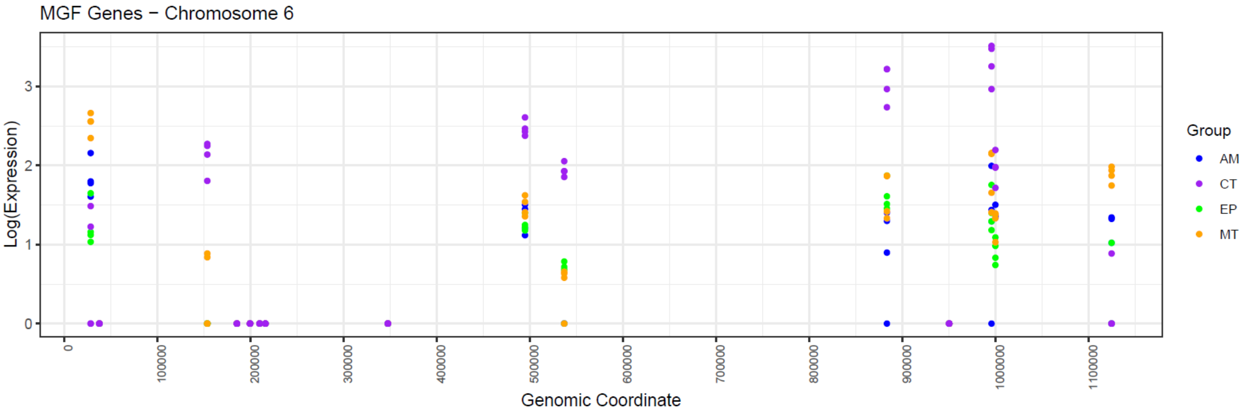
MGF Genes − Chromosome 6
Log(Expression)
Genomic Coordinate
Group
AM
CT
EP
MT

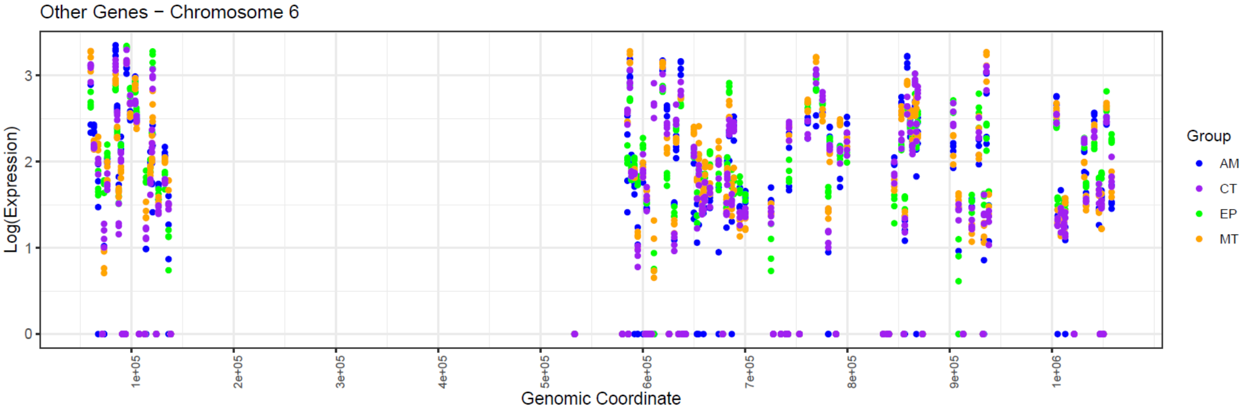
Other Genes − Chromosome 6
Log(Expression)
Genomic Coordinate
Group
AM
CT
EP
MT

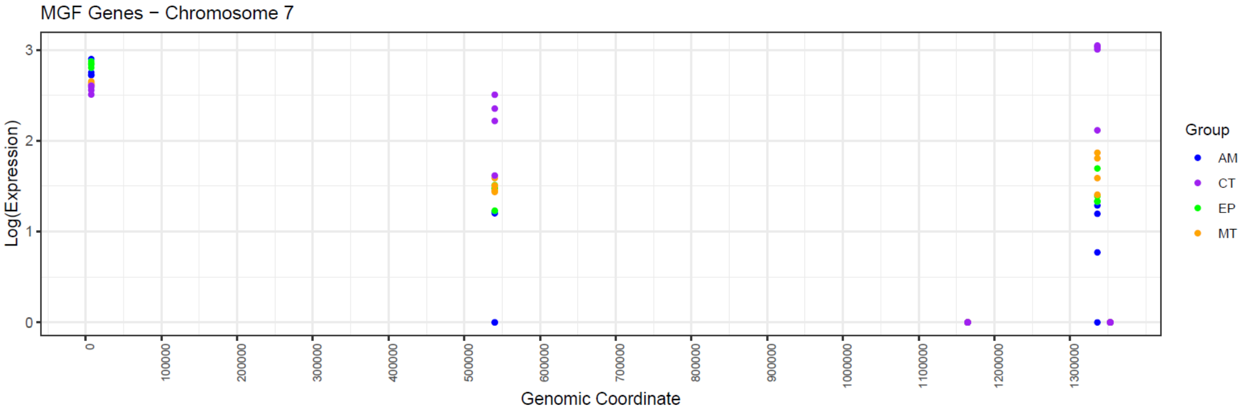
MGF Genes − Chromosome 7
Log(Expression)
Genomic Coordinate
Group
AM
CT
EP
MT

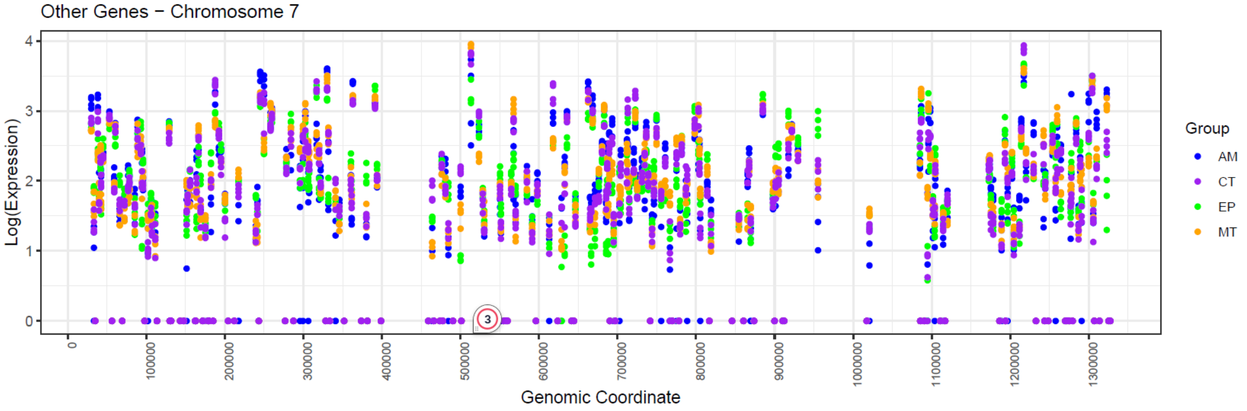
Other Genes − Chromosome 7
Log(Expression)
Genomic Coordinate
Group
AM
CT
EP
MT

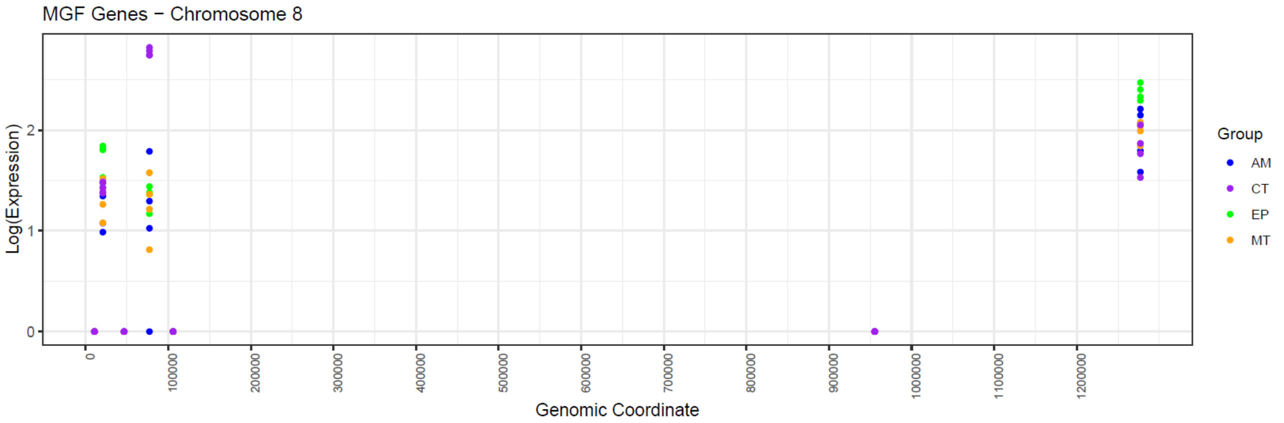
MGF Genes − Chromosome 8
Log(Expression)
Genomic Coordinate
Group
AM
CT
EP
MT

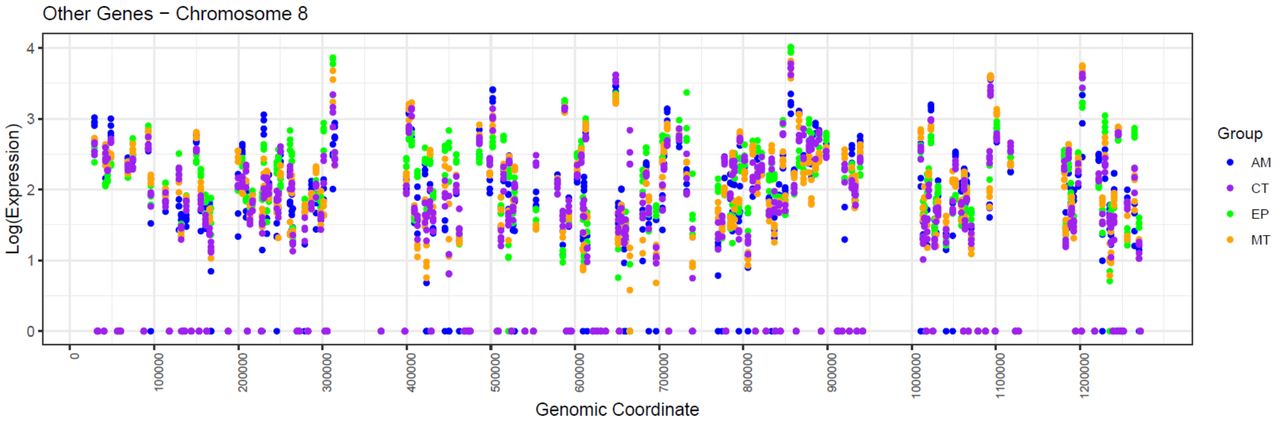
Other Genes − Chromosome 8
Log(Expression)
Genomic Coordinate
Group
AM
CT
EP
MT

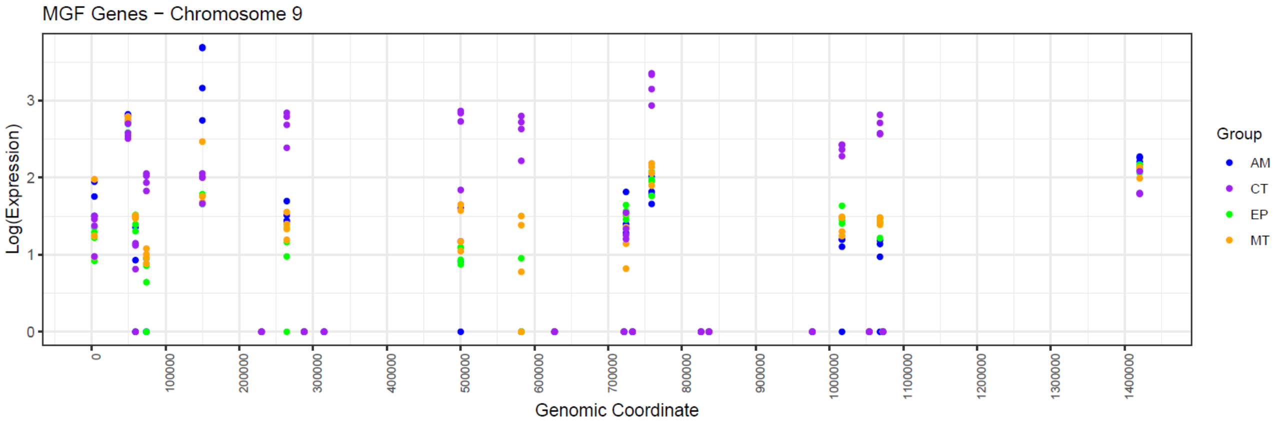
MGF Genes − Chromosome 9
Log(Expression)
Genomic Coordinate
Group
AM
CT
EP
MT

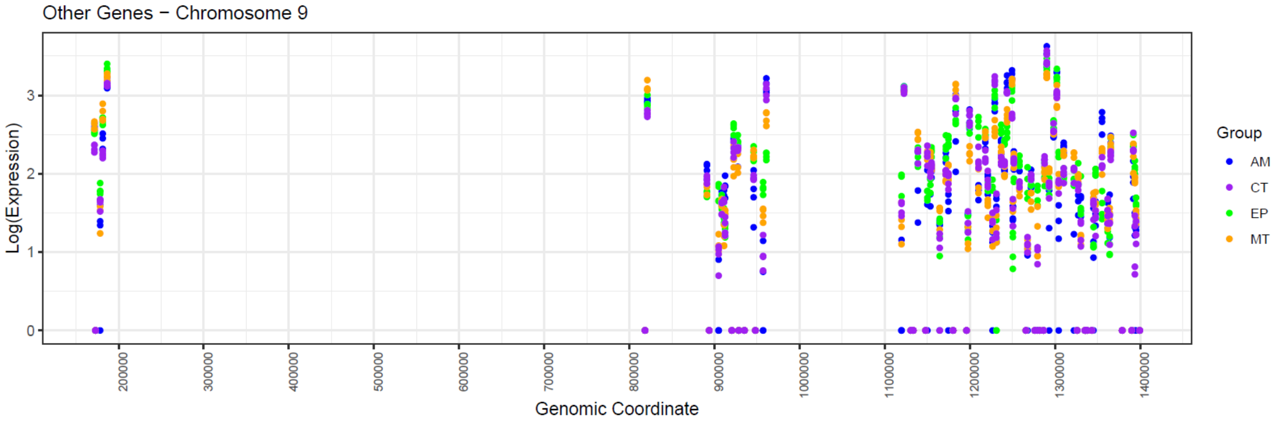
Other Genes − Chromosome 9
Log(Expression)
Genomic Coordinate
Group
AM
CT
EP
MT

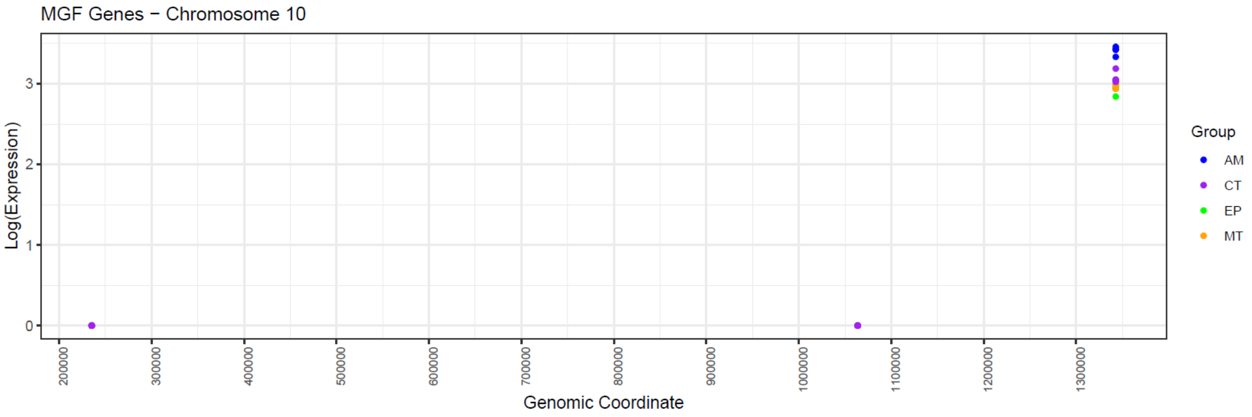
MGF Genes − Chromosome 10
Log(Expression)
0
1
2
3
200000
300000
400000
500000
600000
700000
800000
900000
1000000
1100000
1200000
1300000
Genomic Coordinate
Group
AM
CT
EP
MT

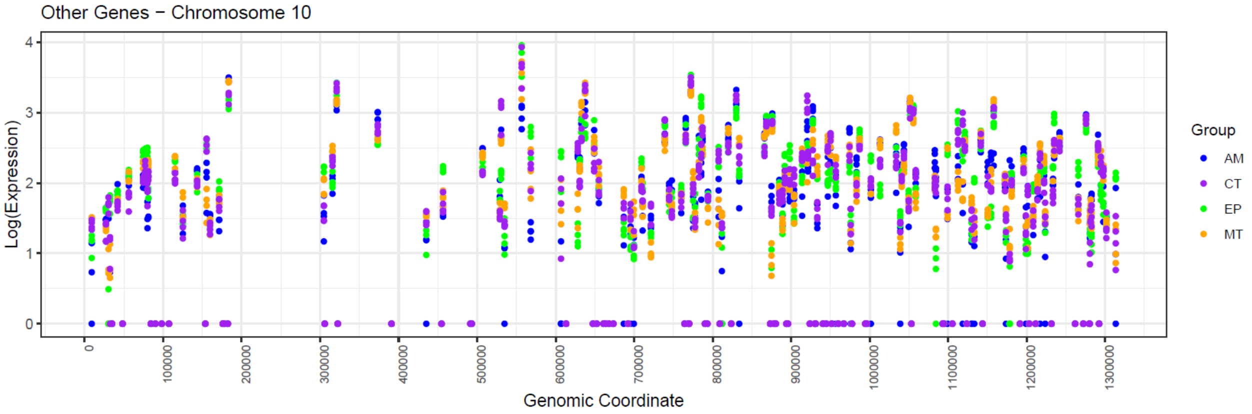
Other Genes − Chromosome 10
Log(Expression)
0
1
2
3
4
0
100000
200000
300000
400000
500000
600000
700000
800000
900000
1000000
1100000
1200000
1300000
Genomic Coordinate
Group
AM
CT
EP
MT

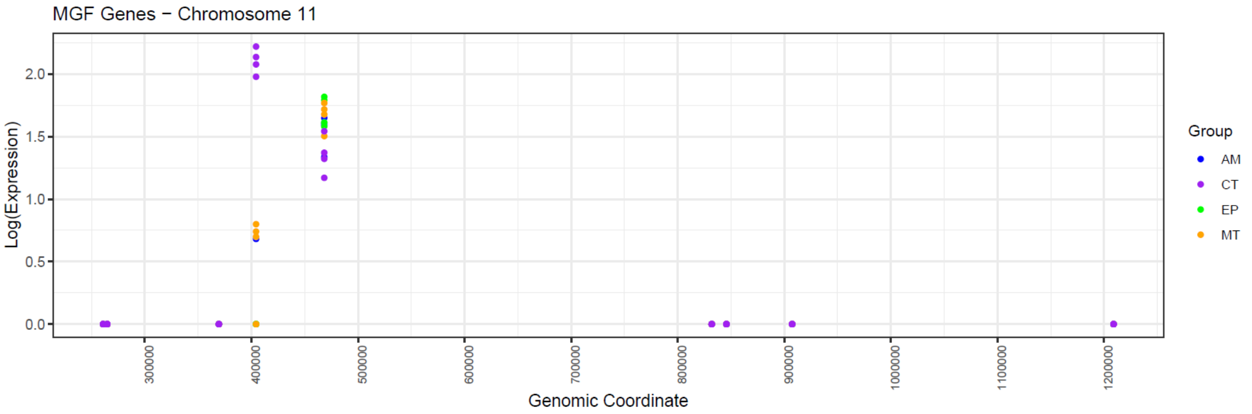
MGF Genes − Chromosome 11
Log(Expression)
0.0
0.5
1.0
1.5
2.0
300000
400000
500000
600000
700000
800000
900000
1000000
1100000
1200000
Genomic Coordinate
Group
AM
CT
EP
MT

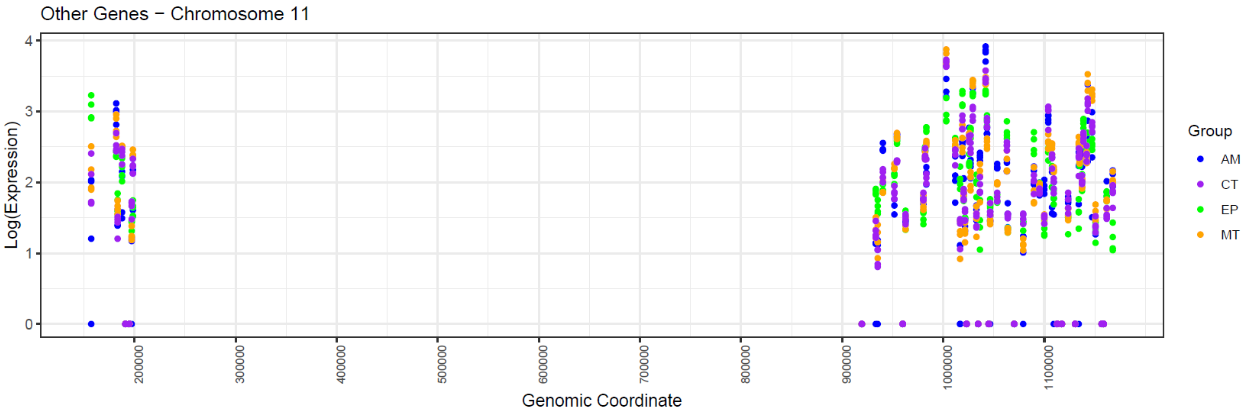
Other Genes − Chromosome 11
Log(Expression)
0
1
2
3
4
200000
300000
400000
500000
600000
700000
800000
900000
1000000
1100000
Genomic Coordinate
Group
AM
CT
EP
MT

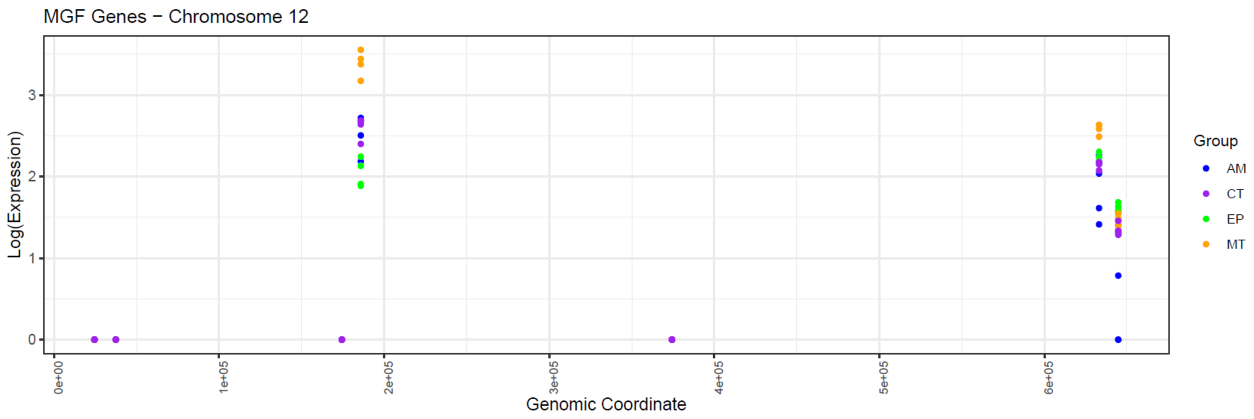
MGF Genes − Chromosome 12
Log(Expression)
0
1
2
3
0e+00
1e+05
2e+05
3e+05
4e+05
5e+05
6e+05
Genomic Coordinate
Group
AM
CT
EP
MT

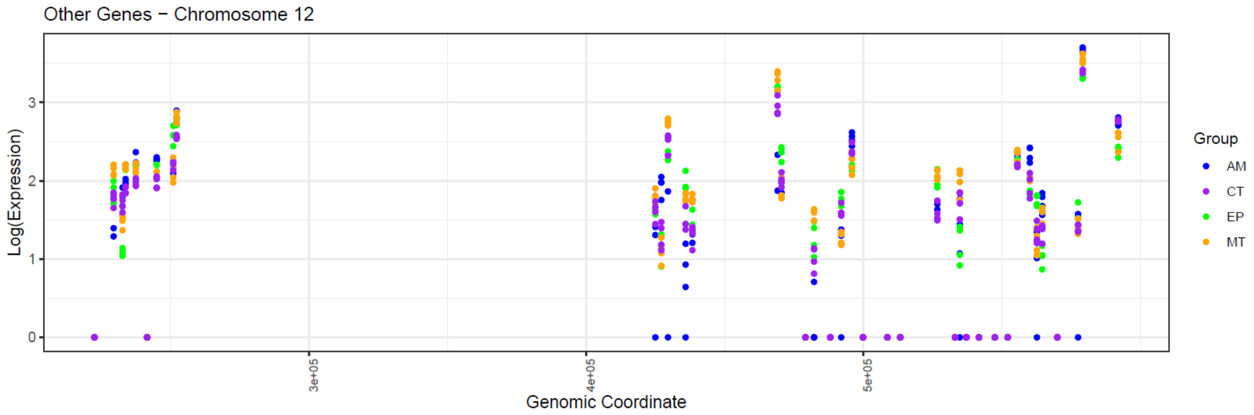
Other Genes − Chromosome 12
Log(Expression)
0
1
2
3
3e+05
4e+05
5e+05
Genomic Coordinate
Group
AM
CT
EP
MT

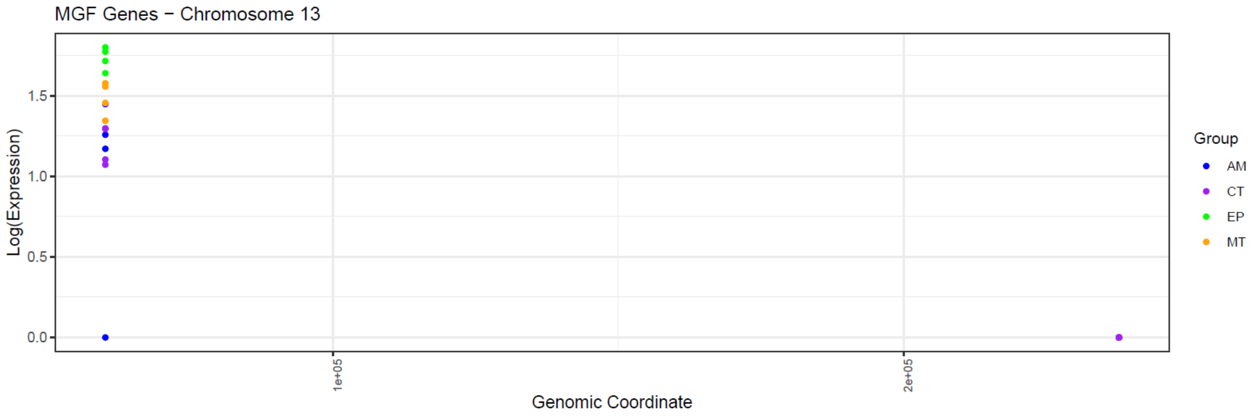
MGF Genes − Chromosome 13
Log(Expression)
0.0
0.5
1.0
1.5
1e+05
2e+05
Genomic Coordinate
Group
AM
CT
EP
MT

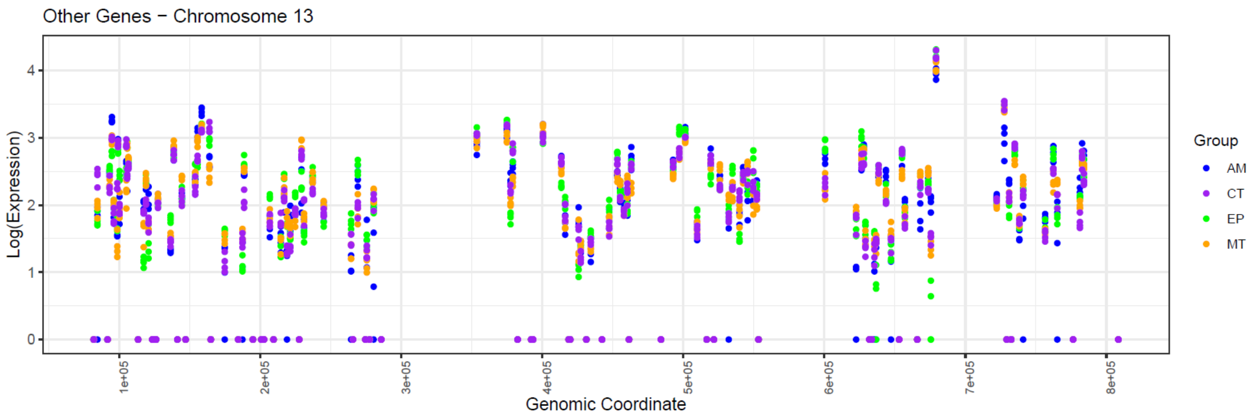
Other Genes − Chromosome 13
Log(Expression)
0
1
2
3
4
1e+05
2e+05
3e+05
4e+05
5e+05
6e+05
7e+05
8e+05
Genomic Coordinate
Group
AM
CT
EP
MT

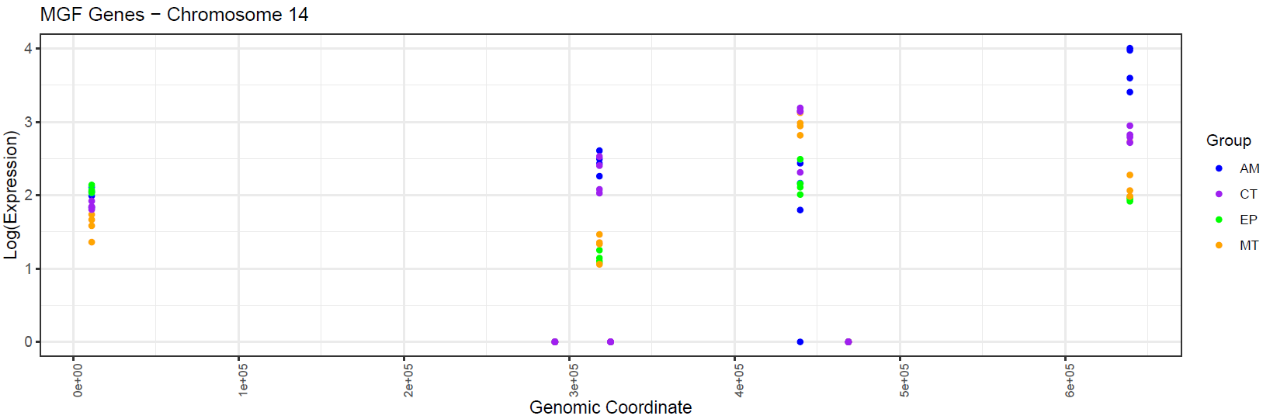
MGF Genes − Chromosome 14
Log(Expression)
0
1
2
3
4
0e+00
1e+05
2e+05
3e+05
4e+05
5e+05
6e+05
Genomic Coordinate
Group
AM
CT
EP
MT

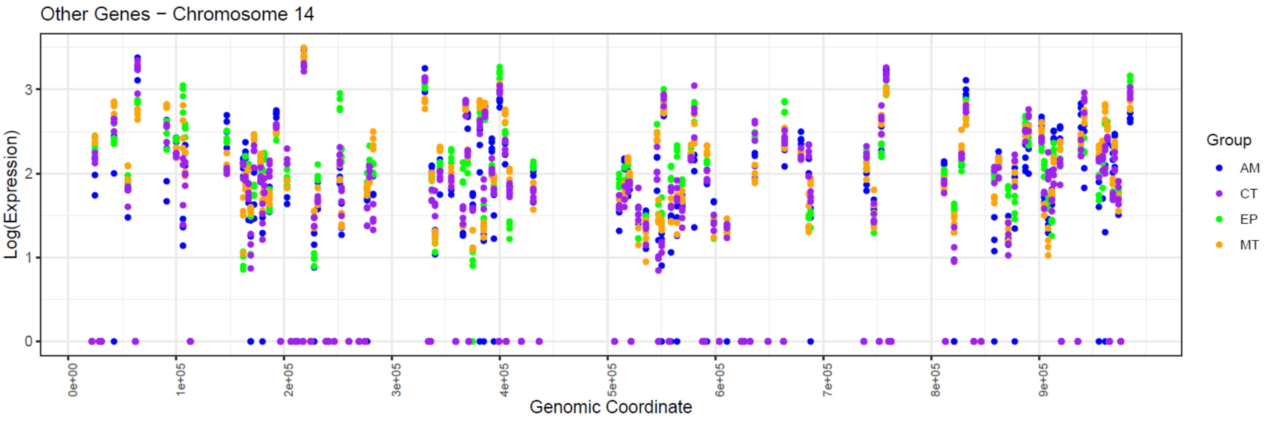
Other Genes − Chromosome 14
Log(Expression)
0
1
2
3
0e+00
1e+05
2e+05
3e+05
4e+05
5e+05
6e+05
7e+05
8e+05
9e+05
Genomic Coordinate
Group
AM
CT
EP
MT

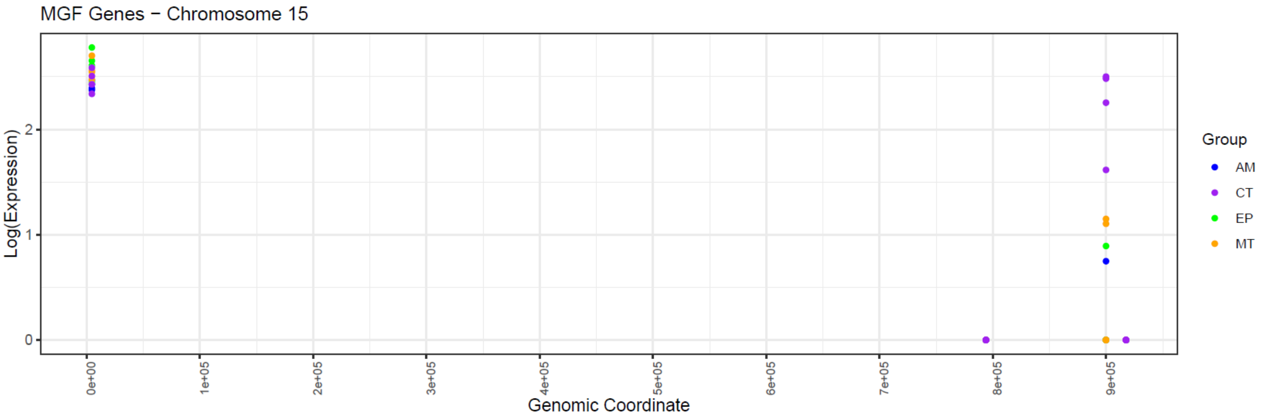
MGF Genes − Chromosome 15
Log(Expression)
0
1
2
0e+00
1e+05
2e+05
3e+05
4e+05
5e+05
6e+05
7e+05
8e+05
9e+05
Genomic Coordinate
Group
AM
CT
EP
MT

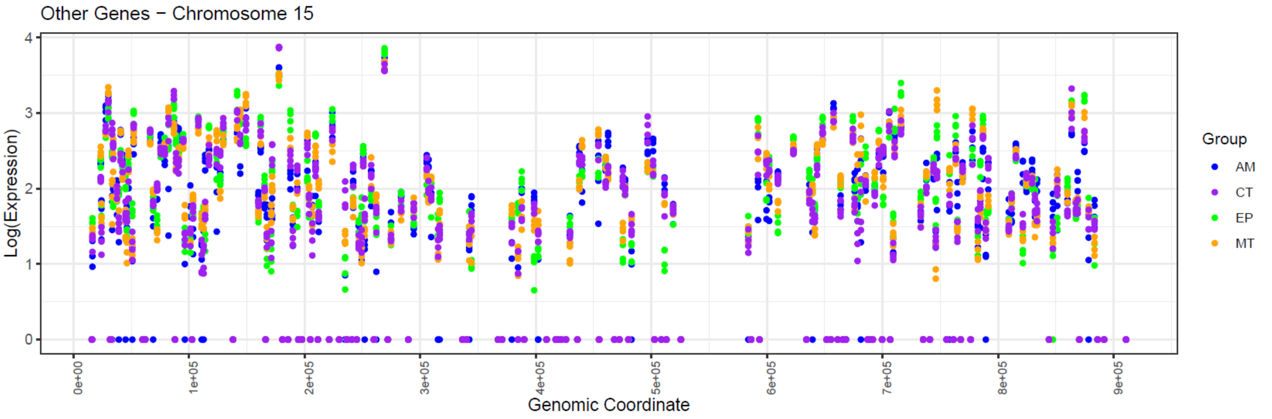
Other Genes − Chromosome 15
Log(Expression)
0
1
2
3
4
0e+00
1e+05
2e+05
3e+05
4e+05
5e+05
6e+05
7e+05
8e+05
9e+05
Genomic Coordinate
Group
AM
CT
EP
MT

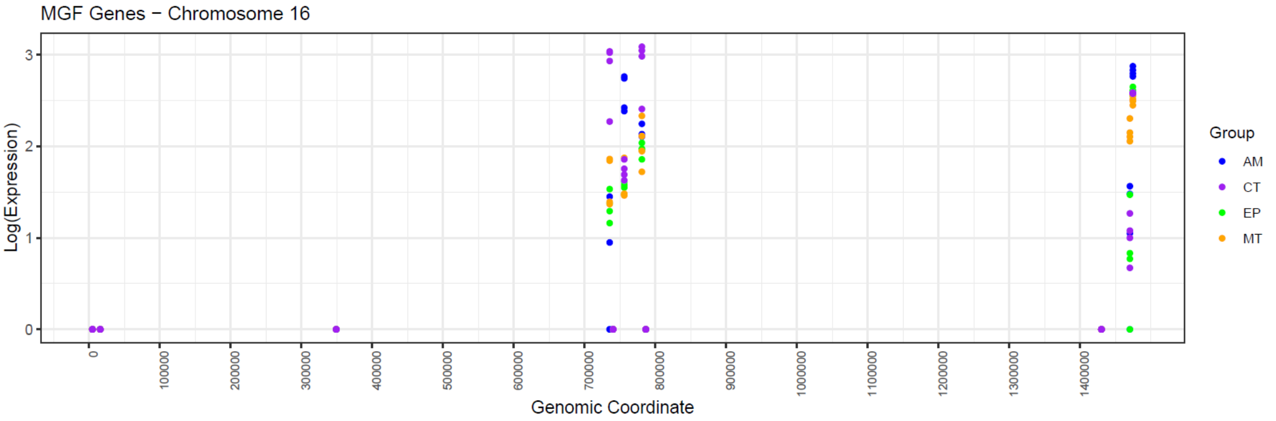
MGF Genes − Chromosome 16
Log(Expression)
Genomic Coordinate
0
100000
200000
300000
400000
500000
600000
700000
800000
900000
1000000
1100000
1200000
1300000
1400000
Group
AM
CT
EP
MT

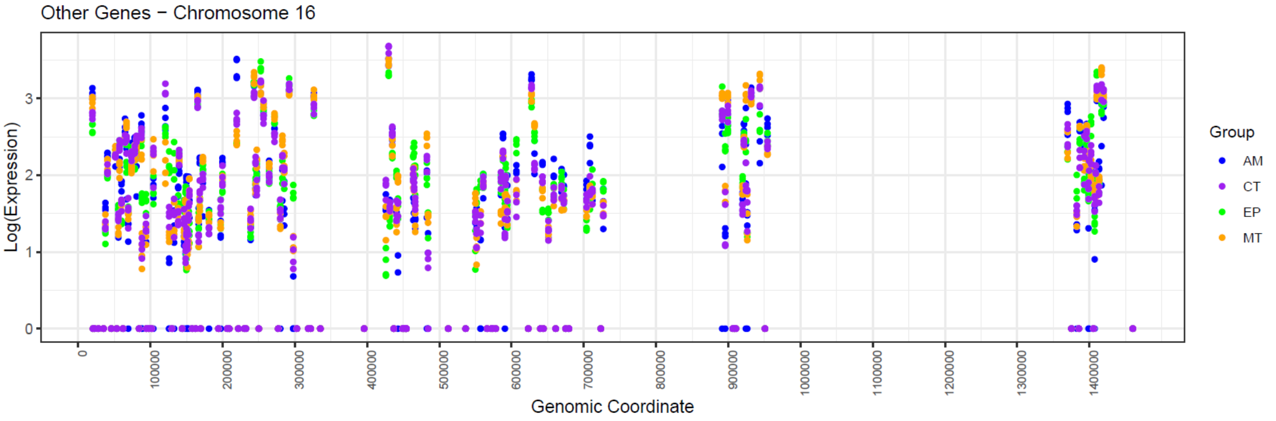
Other Genes − Chromosome 16
Log(Expression)
Genomic Coordinate
0
100000
200000
300000
400000
500000
600000
700000
800000
900000
1000000
1100000
1200000
1300000
1400000
Group
AM
CT
EP
MT

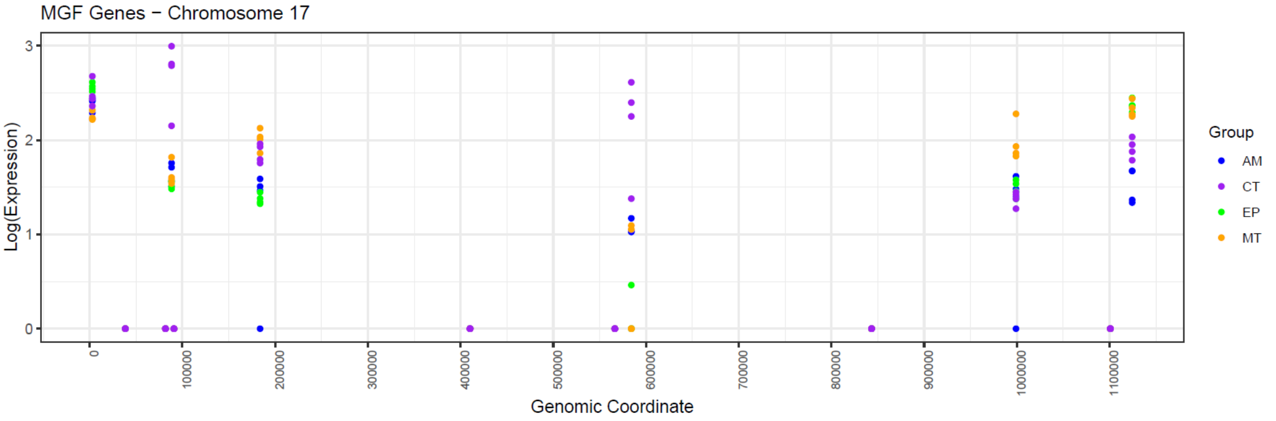
MGF Genes − Chromosome 17
Log(Expression)
Genomic Coordinate
0
100000
200000
300000
400000
500000
600000
700000
800000
900000
1000000
1100000
Group
AM
CT
EP
MT

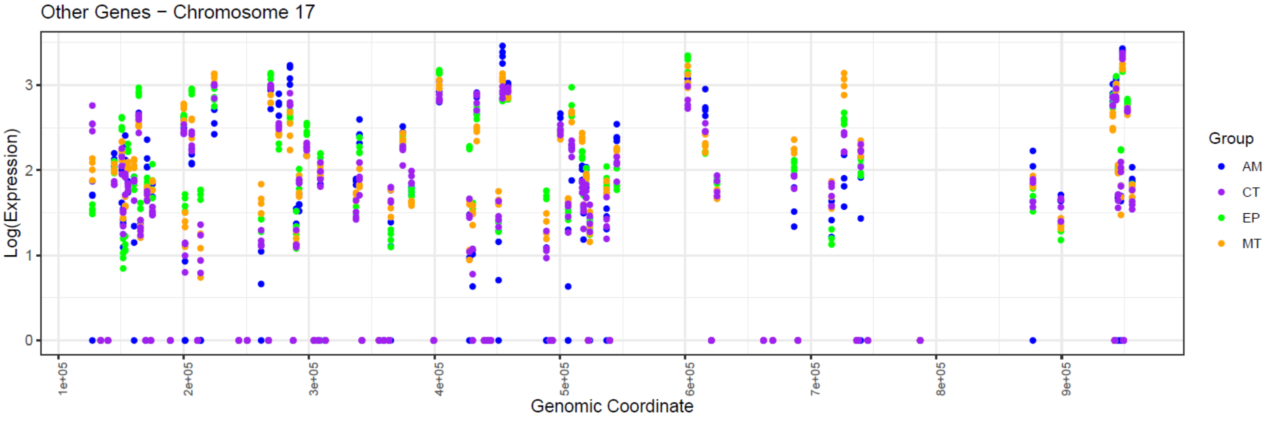
Other Genes − Chromosome 17
Log(Expression)
Genomic Coordinate
1e+05
2e+05
3e+05
4e+05
5e+05
6e+05
7e+05
8e+05
9e+05
Group
AM
CT
EP
MT

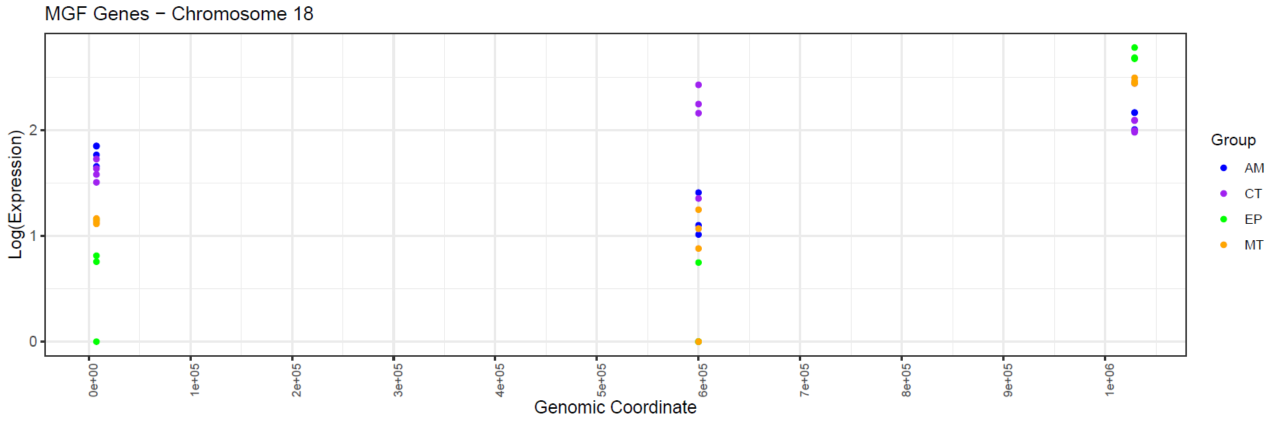
MGF Genes − Chromosome 18
Log(Expression)
Genomic Coordinate
0e+00
1e+05
2e+05
3e+05
4e+05
5e+05
6e+05
7e+05
8e+05
9e+05
1e+06
Group
AM
CT
EP
MT

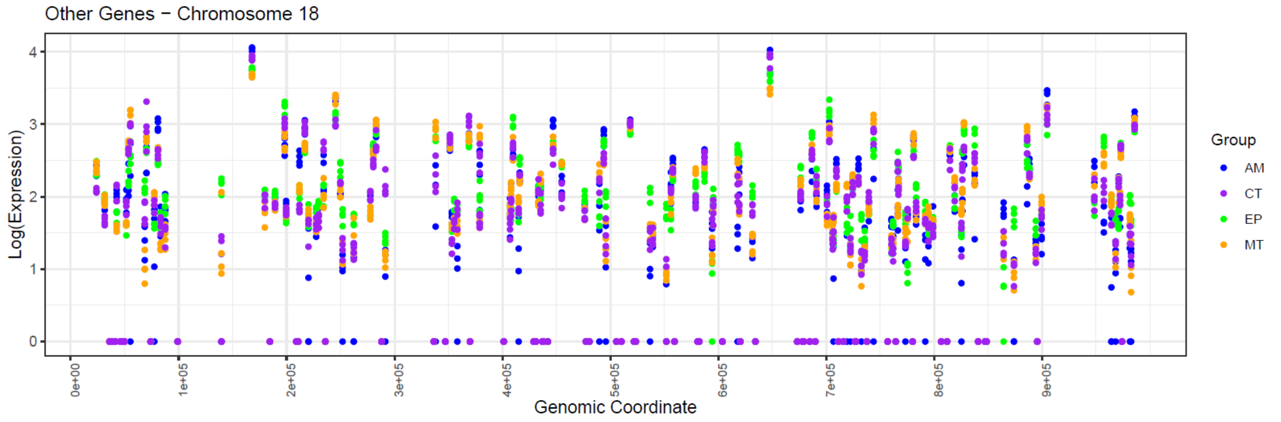
Other Genes − Chromosome 18
Log(Expression)
Genomic Coordinate
0e+00
1e+05
2e+05
3e+05
4e+05
5e+05
6e+05
7e+05
8e+05
9e+05
Group
AM
CT
EP
MT

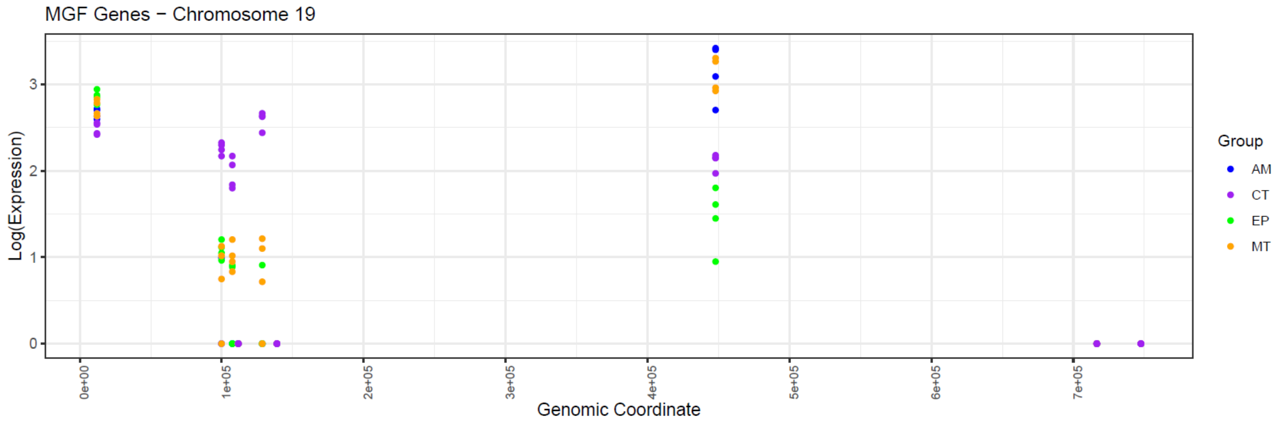
MGF Genes − Chromosome 19
Log(Expression)
Genomic Coordinate
0e+00
1e+05
2e+05
3e+05
4e+05
5e+05
6e+05
7e+05
Group
AM
CT
EP
MT

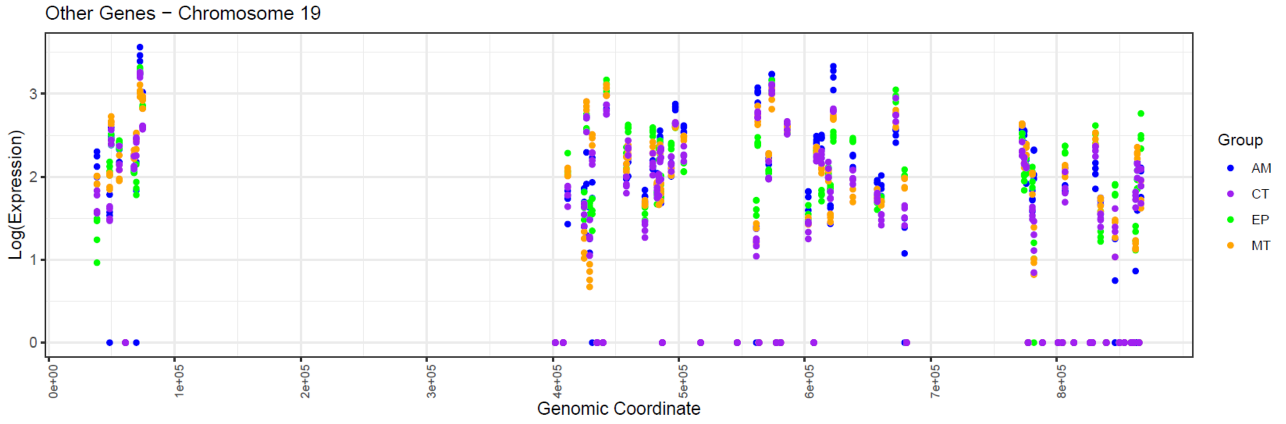
Other Genes − Chromosome 19
Log(Expression)
Genomic Coordinate
0e+00
1e+05
2e+05
3e+05
4e+05
5e+05
6e+05
7e+05
8e+05
Group
AM
CT
EP
MT

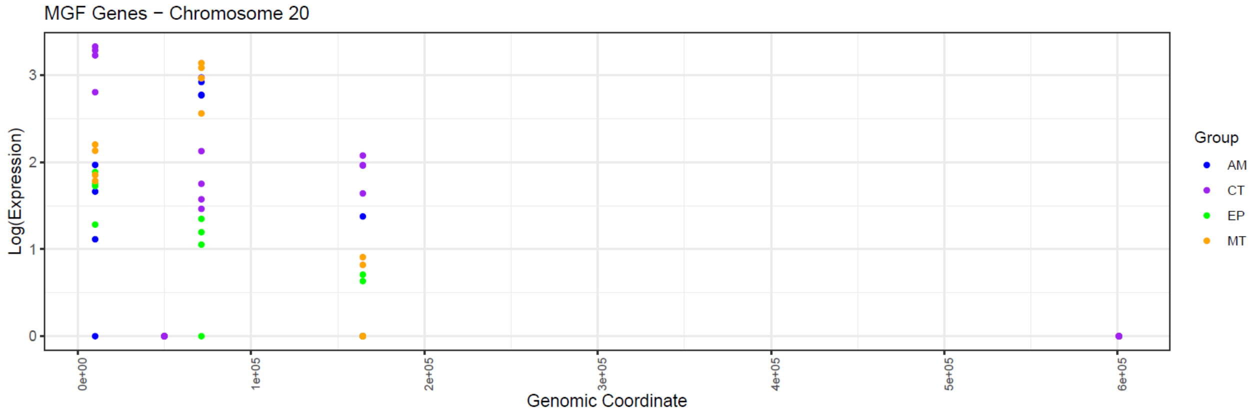
MGF Genes − Chromosome 20
Log(Expression)
Genomic Coordinate
0e+00
1e+05
2e+05
3e+05
4e+05
5e+05
6e+05
0
1
2
3
Group
AM
CT
EP
MT

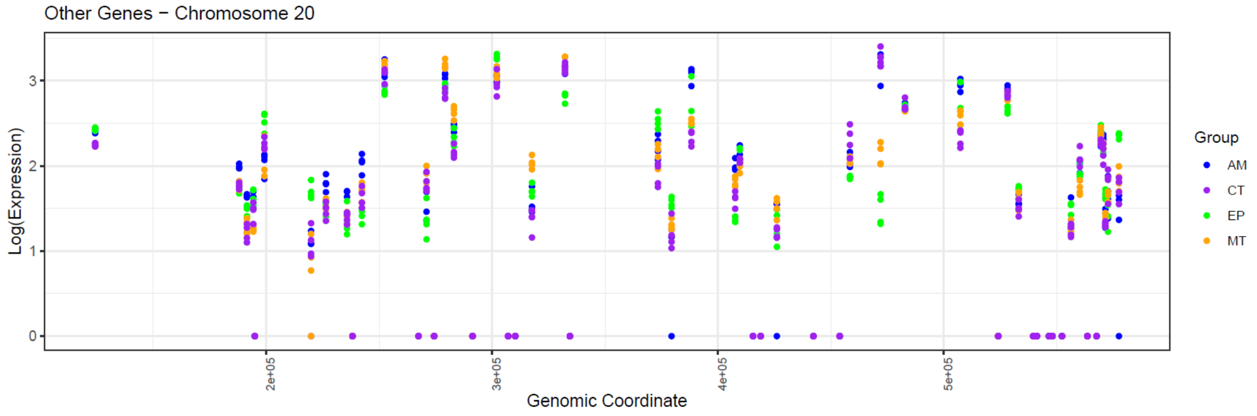
Other Genes − Chromosome 20
Log(Expression)
Genomic Coordinate
2e+05
3e+05
4e+05
5e+05
0
1
2
3
Group
AM
CT
EP
MT

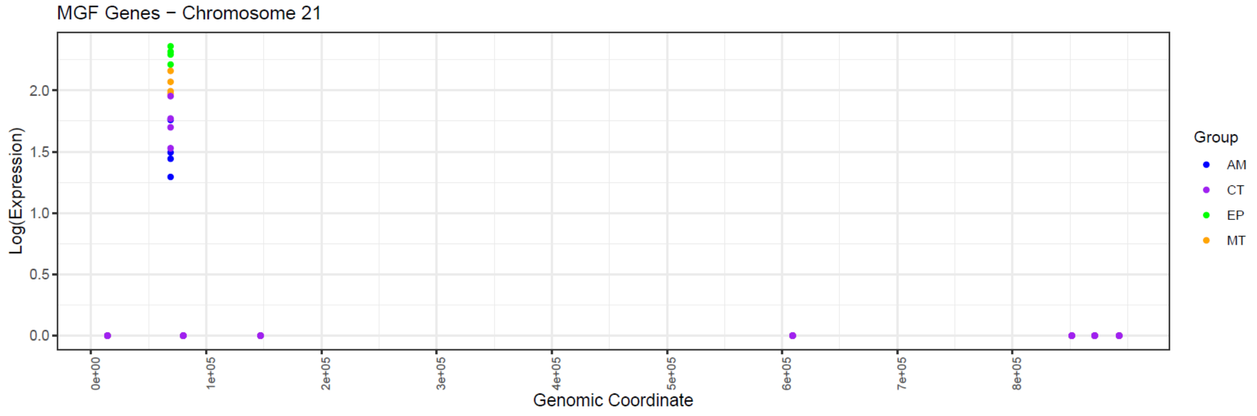
MGF Genes − Chromosome 21
Log(Expression)
Genomic Coordinate
0e+00
1e+05
2e+05
3e+05
4e+05
5e+05
6e+05
7e+05
8e+05
0.0
0.5
1.0
1.5
2.0
Group
AM
CT
EP
MT

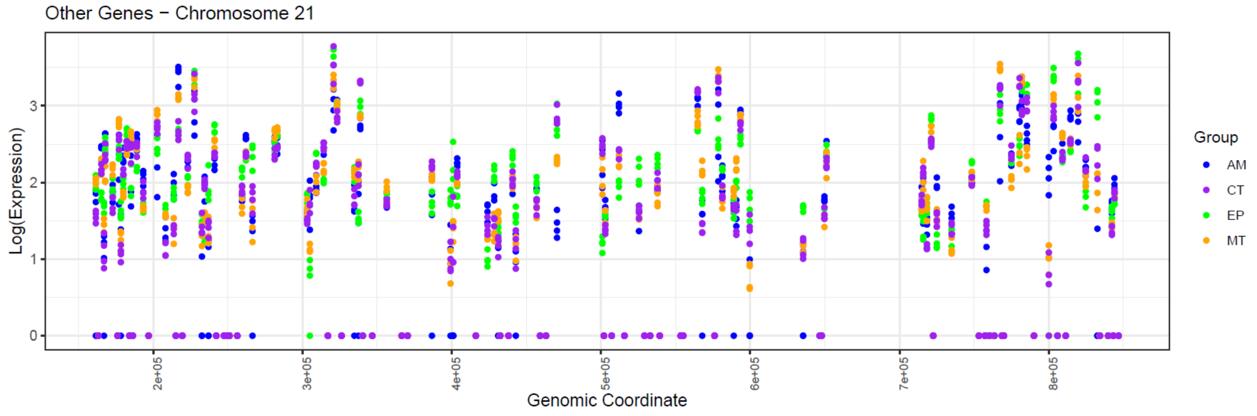
Other Genes − Chromosome 21
Log(Expression)
Genomic Coordinate
2e+05
3e+05
4e+05
5e+05
6e+05
7e+05
8e+05
0
1
2
3
Group
AM
CT
EP
MT

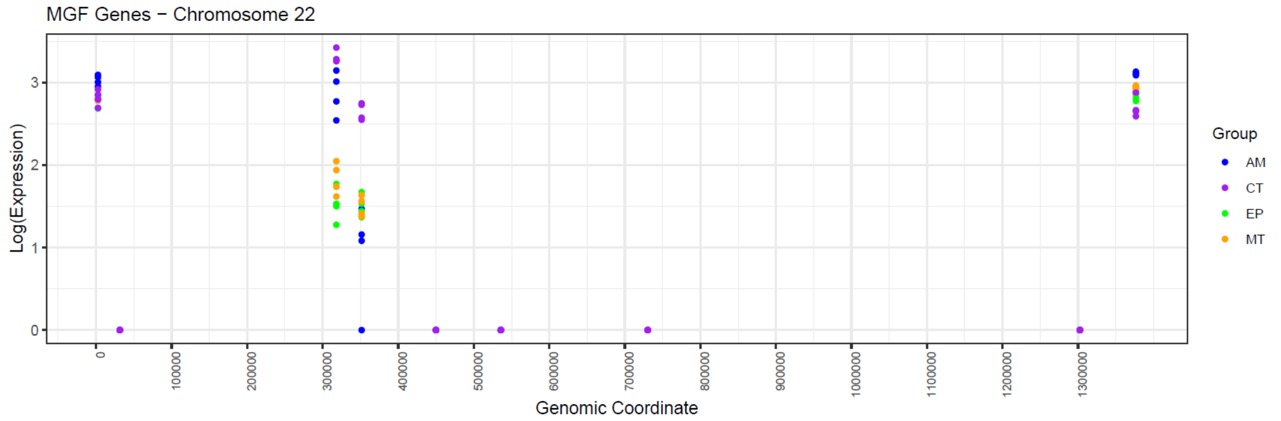
MGF Genes − Chromosome 22
Log(Expression)
0
1
2
3
Genomic Coordinate
0
100000
200000
300000
400000
500000
600000
700000
800000
900000
1000000
1100000
1200000
1300000
Group
AM
CT
EP
MT

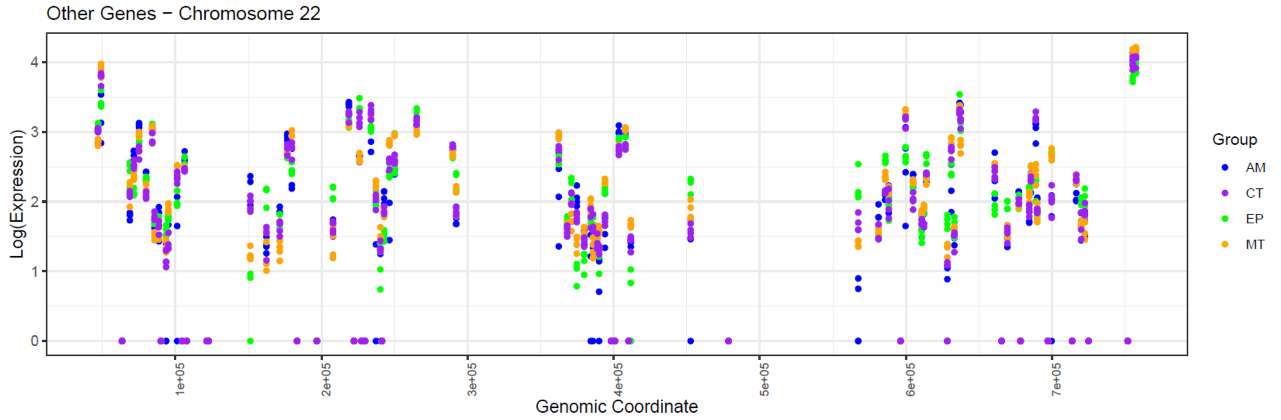
Other Genes − Chromosome 22
Log(Expression)
0
1
2
3
4
Genomic Coordinate
1e+05
2e+05
3e+05
4e+05
5e+05
6e+05
7e+05
Group
AM
CT
EP
MT

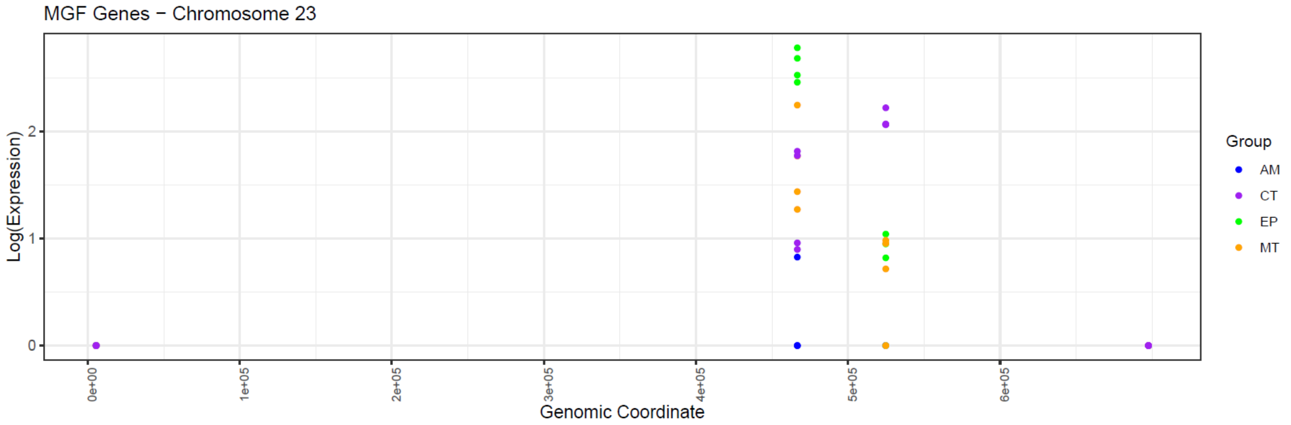
MGF Genes − Chromosome 23
Log(Expression)
0
1
2
Genomic Coordinate
0e+00
1e+05
2e+05
3e+05
4e+05
5e+05
6e+05
Group
AM
CT
EP
MT

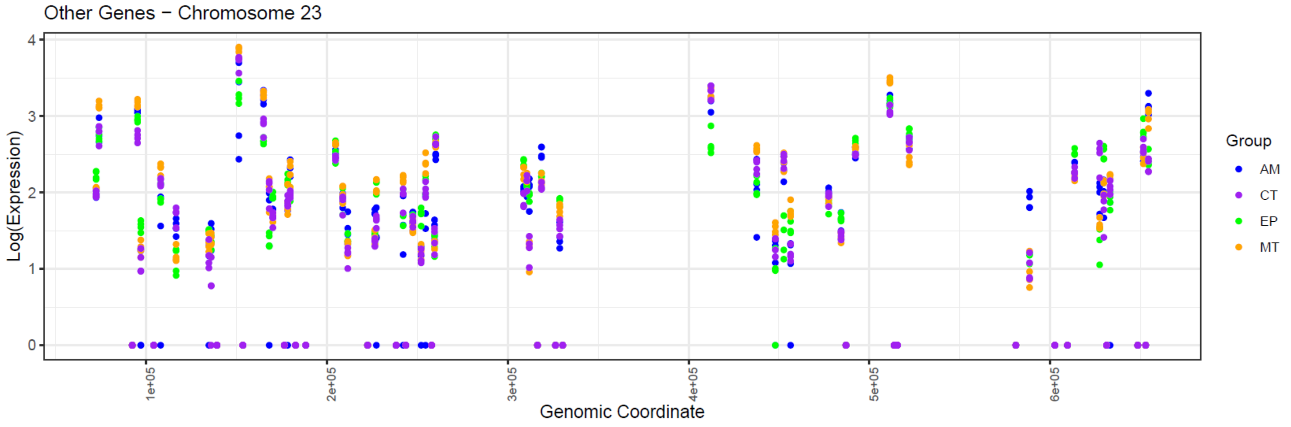
Other Genes − Chromosome 23
Log(Expression)
0
1
2
3
4
Genomic Coordinate
1e+05
2e+05
3e+05
4e+05
5e+05
6e+05
Group
AM
CT
EP
MT

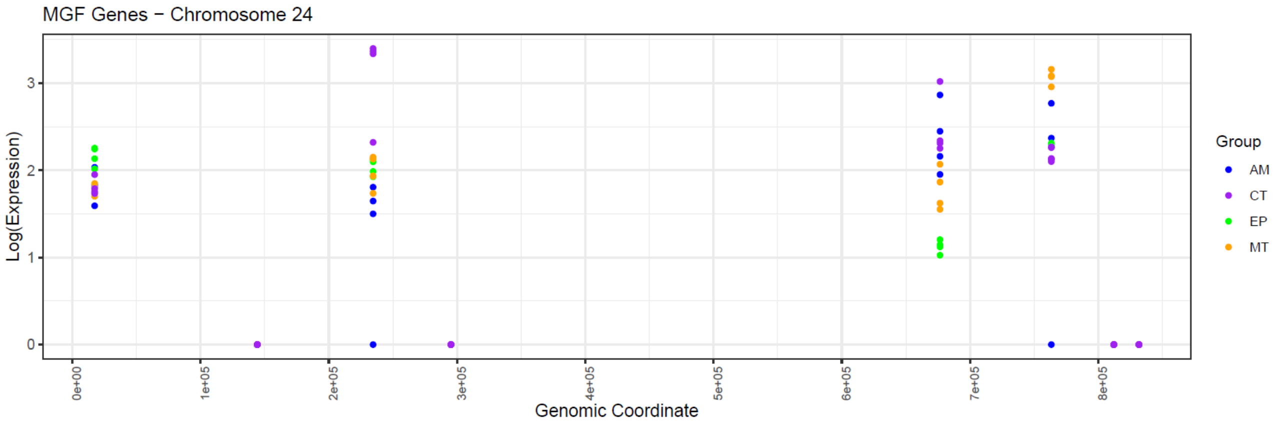
MGF Genes − Chromosome 24
Log(Expression)
0
1
2
3
0e+00
1e+05
2e+05
3e+05
4e+05
5e+05
6e+05
7e+05
8e+05
Genomic Coordinate
Group
AM
CT
EP
MT

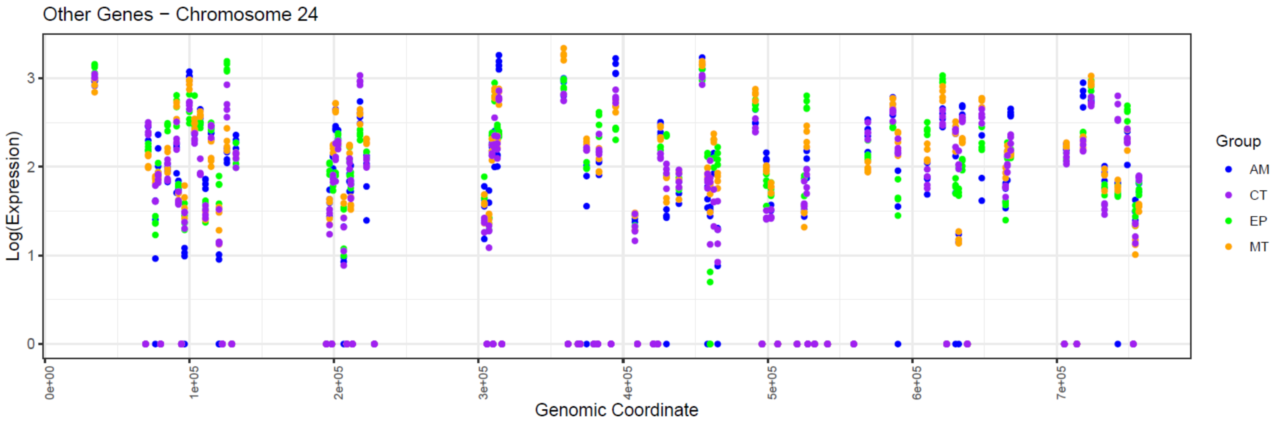
Other Genes − Chromosome 24
Log(Expression)
0
1
2
3
0e+00
1e+05
2e+05
3e+05
4e+05
5e+05
6e+05
7e+05
Genomic Coordinate
Group
AM
CT
EP
MT

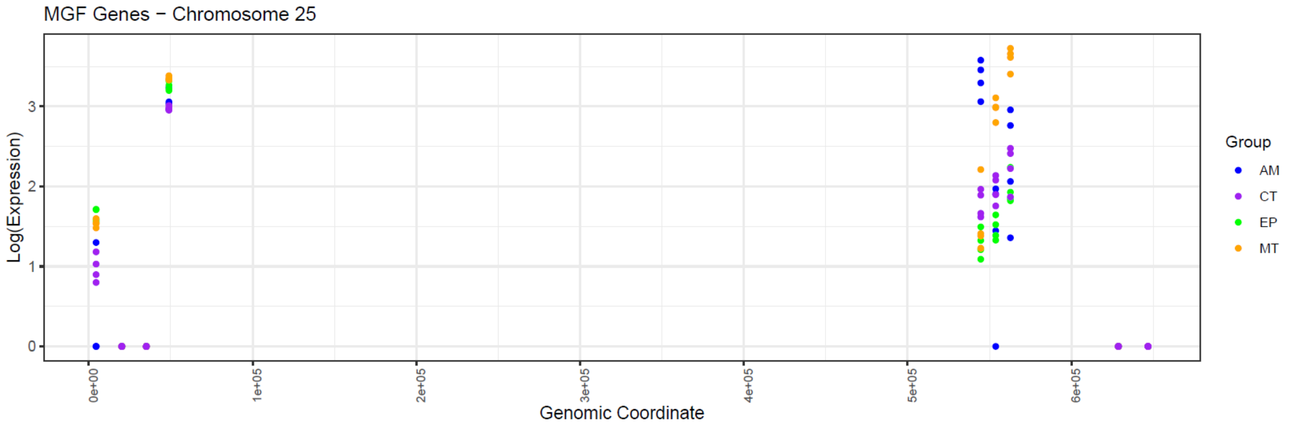
MGF Genes − Chromosome 25
Log(Expression)
0
1
2
3
0e+00
1e+05
2e+05
3e+05
4e+05
5e+05
6e+05
Genomic Coordinate
Group
AM
CT
EP
MT

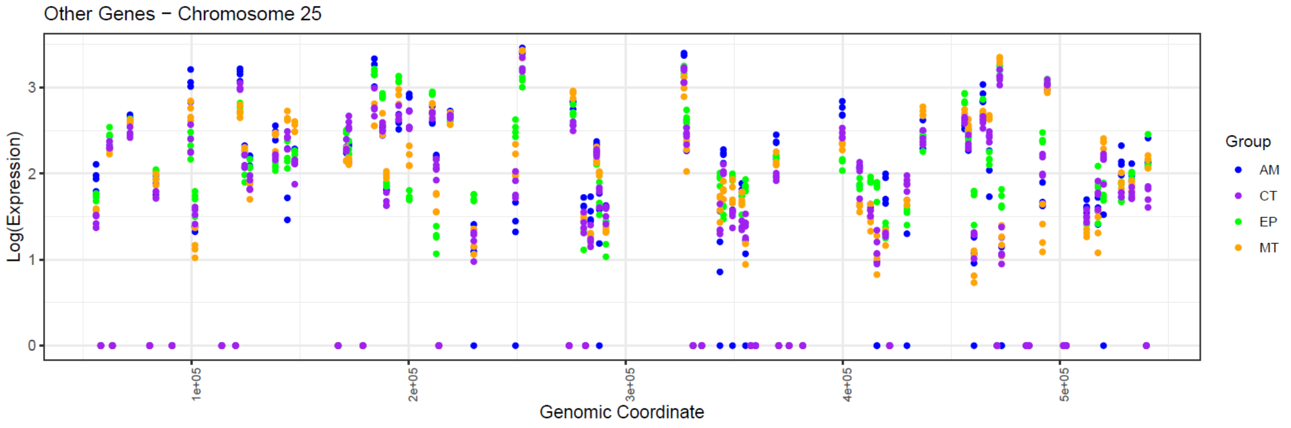
Other Genes − Chromosome 25
Log(Expression)
0
1
2
3
1e+05
2e+05
3e+05
4e+05
5e+05
Genomic Coordinate
Group
AM
CT
EP
MT

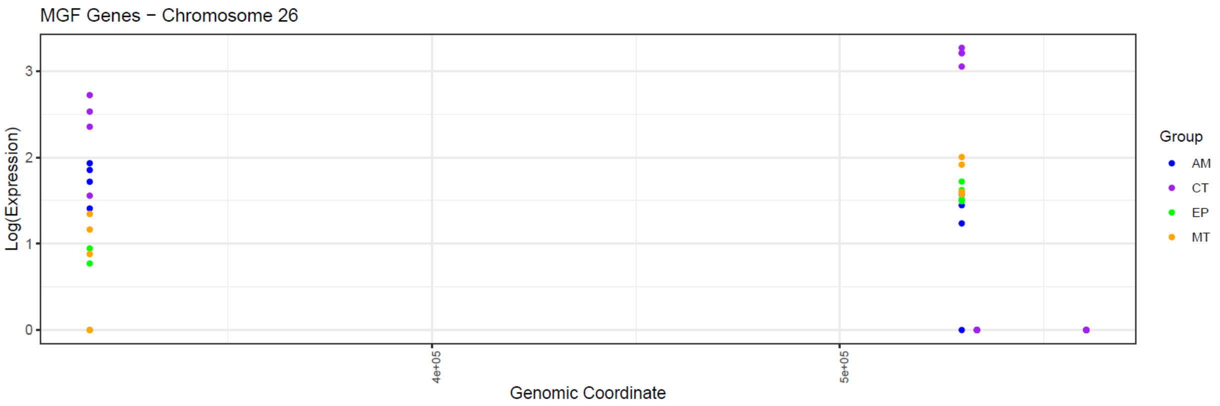
MGF Genes − Chromosome 26
Log(Expression)
0
1
2
3
4e+05
5e+05
Genomic Coordinate
Group
AM
CT
EP
MT

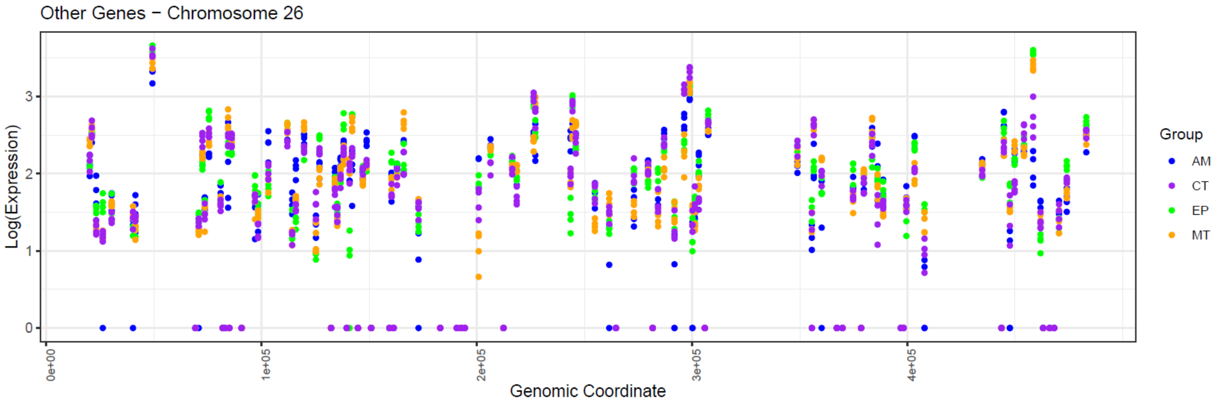
Other Genes − Chromosome 26
Log(Expression)
0
1
2
3
0e+00
1e+05
2e+05
3e+05
4e+05
Genomic Coordinate
Group
AM
CT
EP
MT

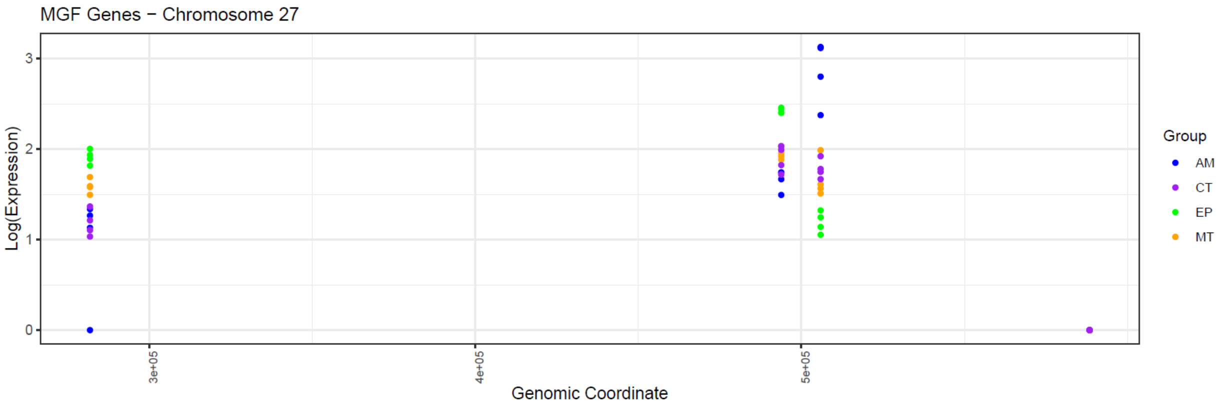
MGF Genes − Chromosome 27
Log(Expression)
0
1
2
3
3e+05
4e+05
5e+05
Genomic Coordinate
Group
AM
CT
EP
MT

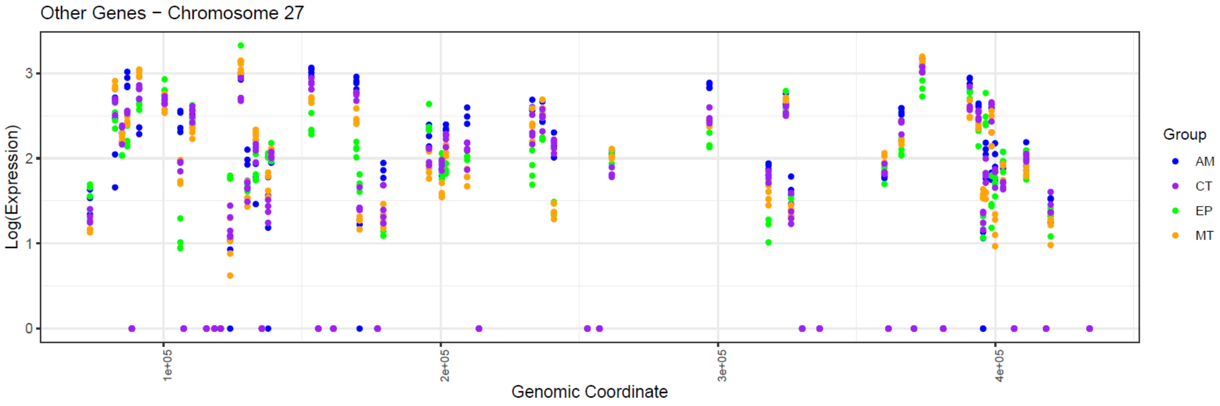
Other Genes − Chromosome 27
Log(Expression)
0
1
2
3
1e+05
2e+05
3e+05
4e+05
Genomic Coordinate
Group
AM
CT
EP
MT

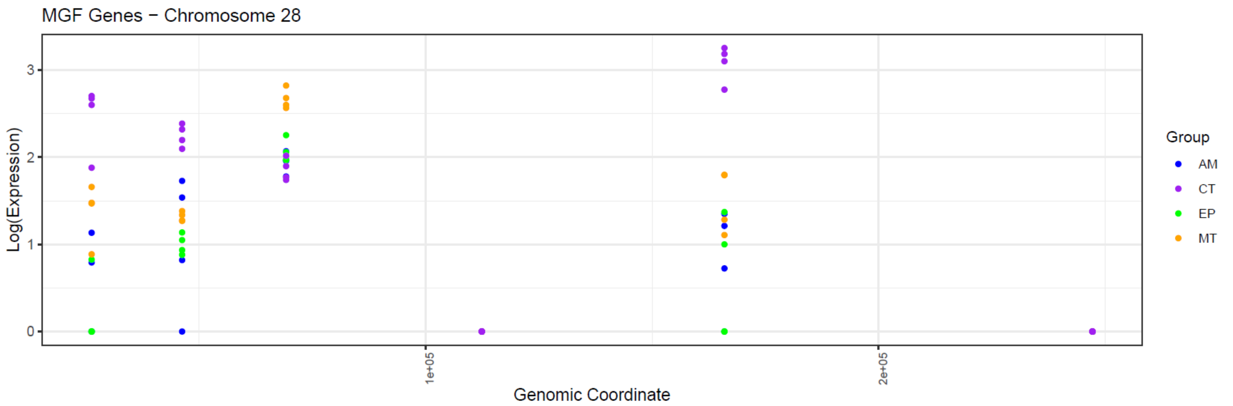
MGF Genes − Chromosome 28
Log(Expression)
Genomic Coordinate
1e+05
2e+05
Group
AM
CT
EP
MT

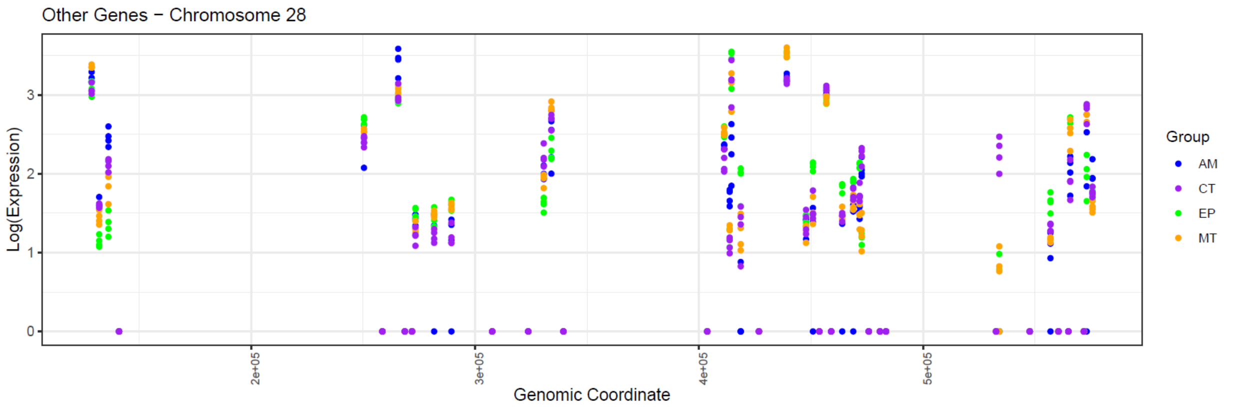
Other Genes − Chromosome 28
Log(Expression)
Genomic Coordinate
2e+05
3e+05
4e+05
5e+05
Group
AM
CT
EP
MT

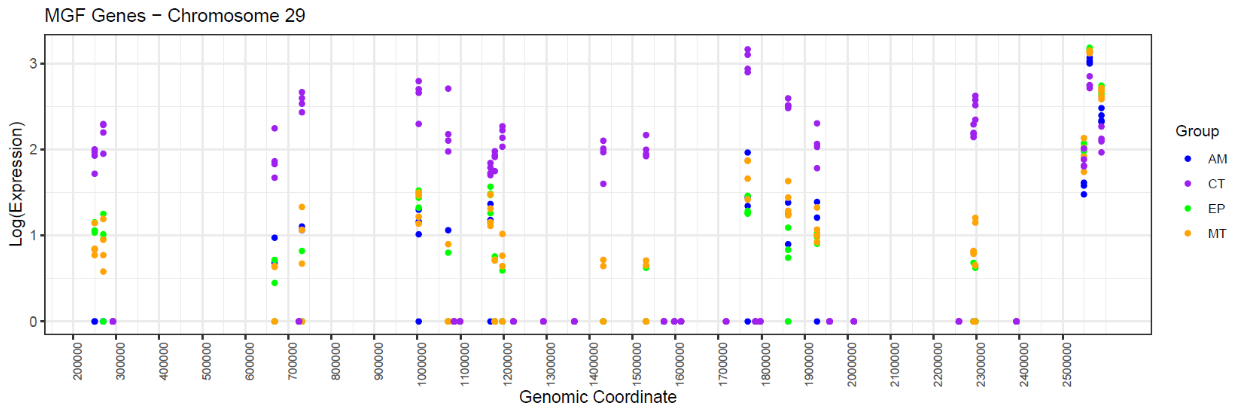
MGF Genes − Chromosome 29
Log(Expression)
Genomic Coordinate
Group
AM
CT
EP
MT

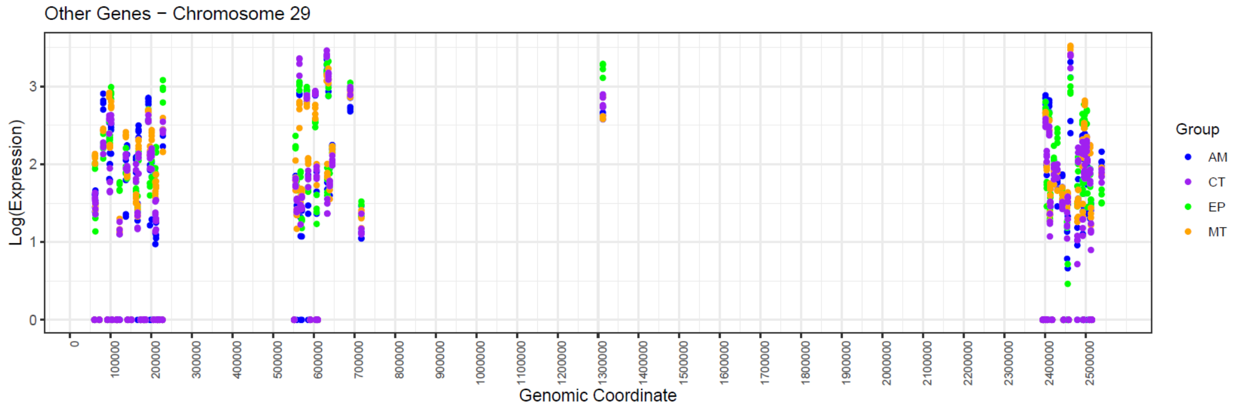
Other Genes − Chromosome 29
Log(Expression)
Genomic Coordinate
Group
AM
CT
EP
MT

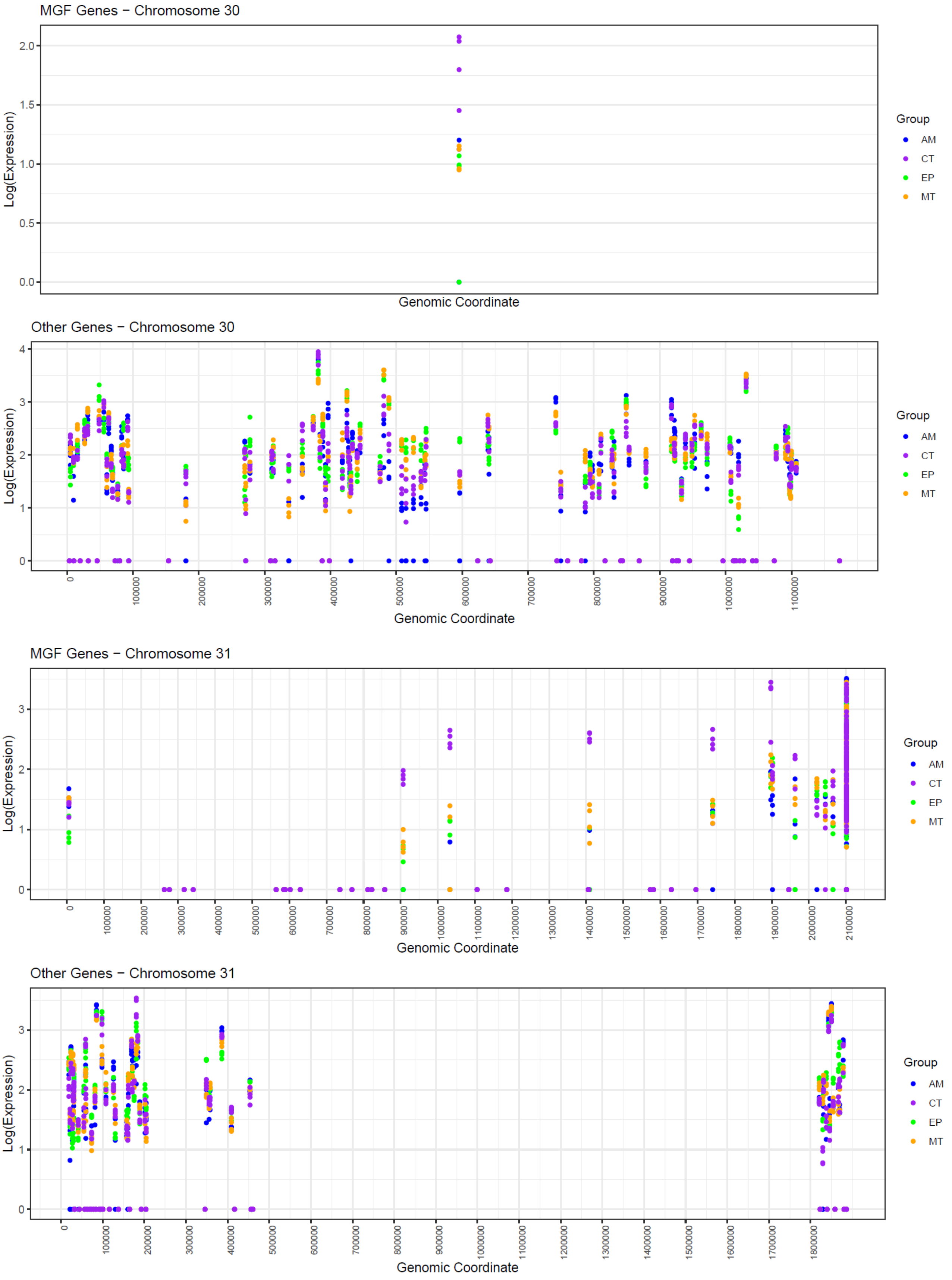
MGF Genes − Chromosome 30
Log(Expression)
Genomic Coordinate
Group
AM
CT
EP
MT
Other Genes − Chromosome 30
Log(Expression)
Genomic Coordinate
Group
AM
CT
EP
MT
MGF Genes − Chromosome 31
Log(Expression)
Genomic Coordinate
Group
AM
CT
EP
MT
Other Genes − Chromosome 31
Log(Expression)
Genomic Coordinate
Group
AM
CT
EP
MT

Supplementary Figure 11. Transcriptomic and proteomic analysis of MGF gene expression in *T. cruzi*. RNA-seq and proteomic data confirm MGF gene expression across multiple chromosomes. The circos plot displays all 31 chromosomes, showing protein (Log_CTp) and RNA (Log_CTr) abundance by genomic coordinates. Red dots represent the log2 abundance of MGF genes, while black dots indicate non-MGF genes, i.e., housekeeping genes (HK).

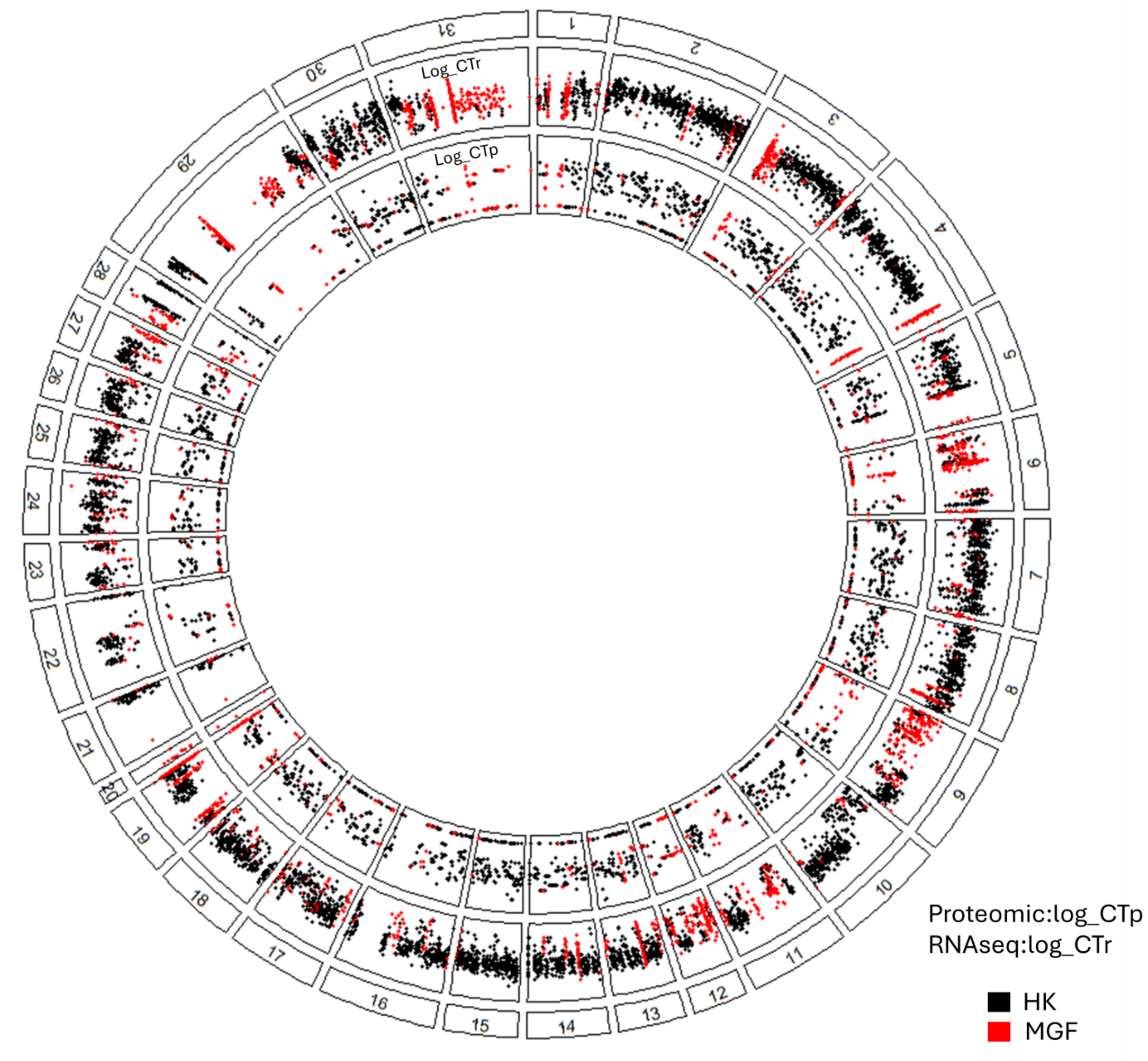

Supplementary Figure 12. Distribution of protein and RNA expression of MGF and housekeeping genes. The density plots show the distribution of log2-transformed expression of MASP, mucin, RHS, DGF-1, GP63, and housekeeping genes in CTs. Data is based on three biological replicates.

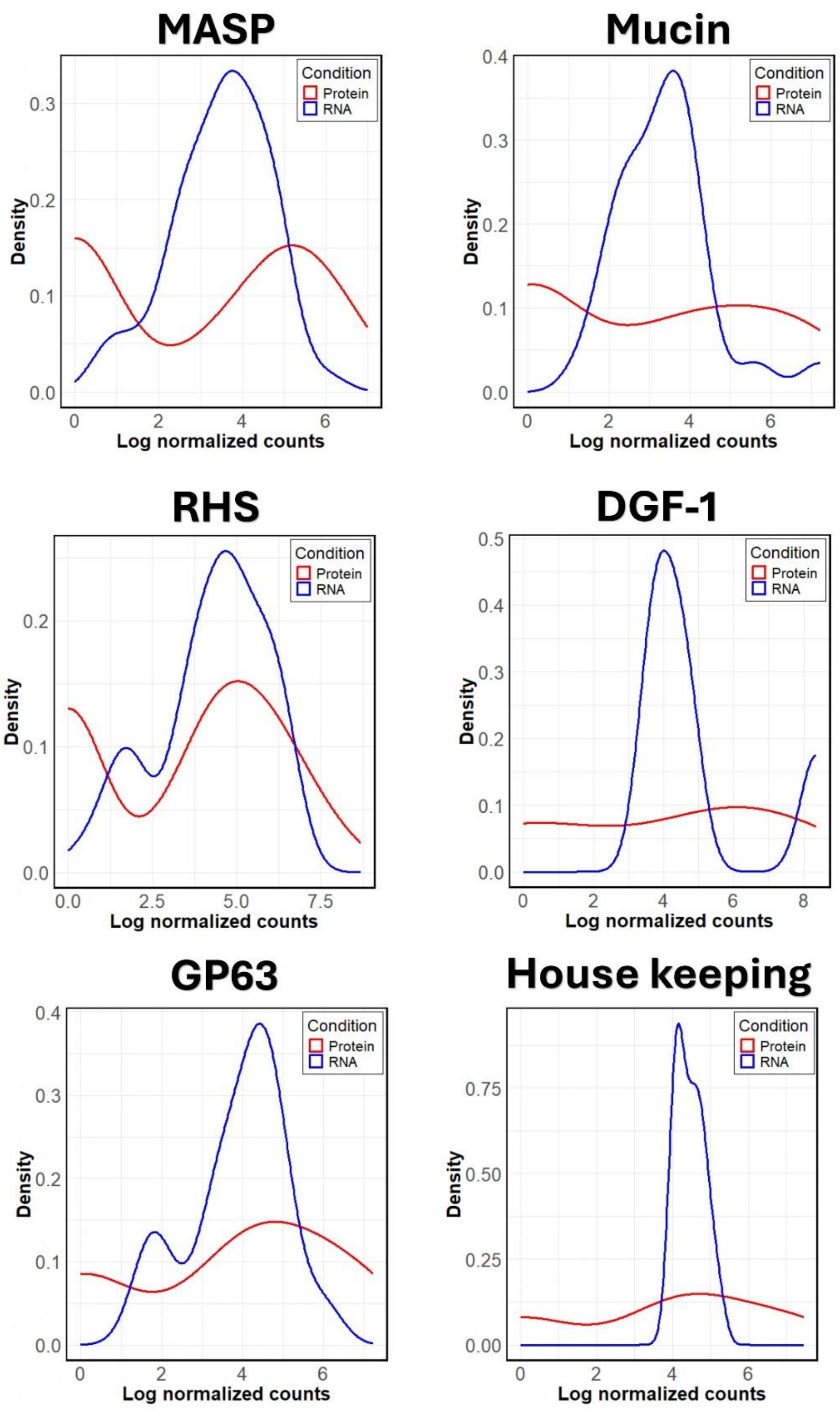

Supplementary Figure 13. Comparison of RNA-seq datasets from HiSeq and MiSeq platforms against *T. cruzi* CTs. A) Heatmaps showing normalized expression counts for each MGF, comparing HiSeq and MiSeq datasets. Data show many MGF genes expressed in the parasite population. Variation in abundance among datasets is also visible. B) Bar plots showing the number of expressed genes per MGF, with HiSeq data in pink and MiSeq data in blue. Data is based on three biological replicates for each platform.

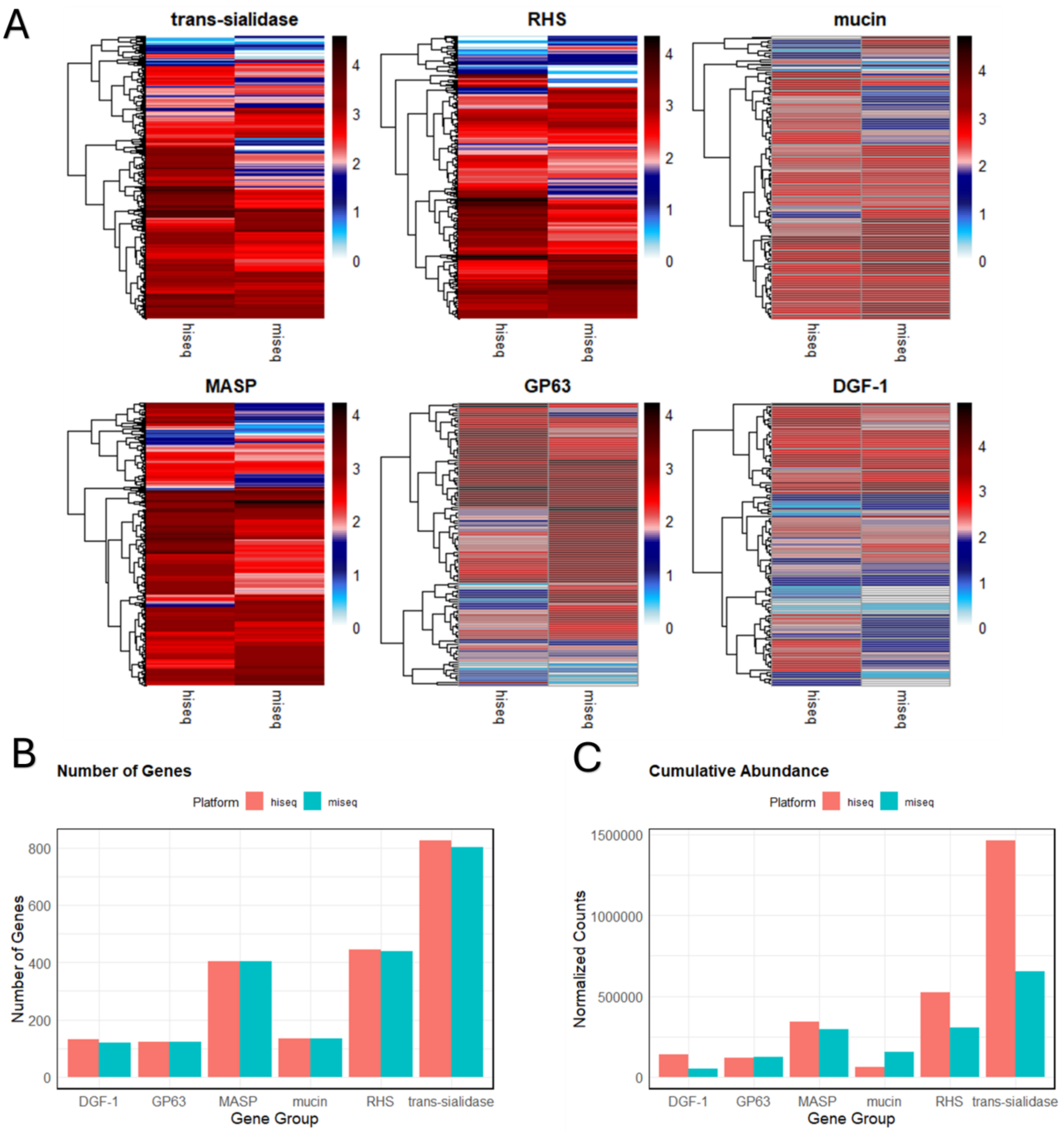

Supplementary Figure 14. MGF expression across multiple rounds of cell infection. A) Volcano plots show differentially expressed transcripts across multiple generations of CTs infections in H9-C2 or CTs from infection in HEK293T cells. The X-axis represents $\log_2$ fold change, while the Y-axis represents $-\log_{10}$(p-value). Transcripts showing significant expression ($\log_2 \geq 1$, *p*-value $\leq .05$) are highlighted in red. B) The bar plot displays the most abundant MGF transcripts across all samples. Their expression is predominantly observed in G4 of CTs from H9-C2 or CTs derived from HEK293T cells. C) The graph represents the number of MGF transcripts expressed per generation after each round of infection. The X-axis represents experimental groups (G0–G4), while the Y-axis indicates the number of MGF genes expressed. G0 is MT, G1-G4 are CTs. Data is based on three biological replicates.

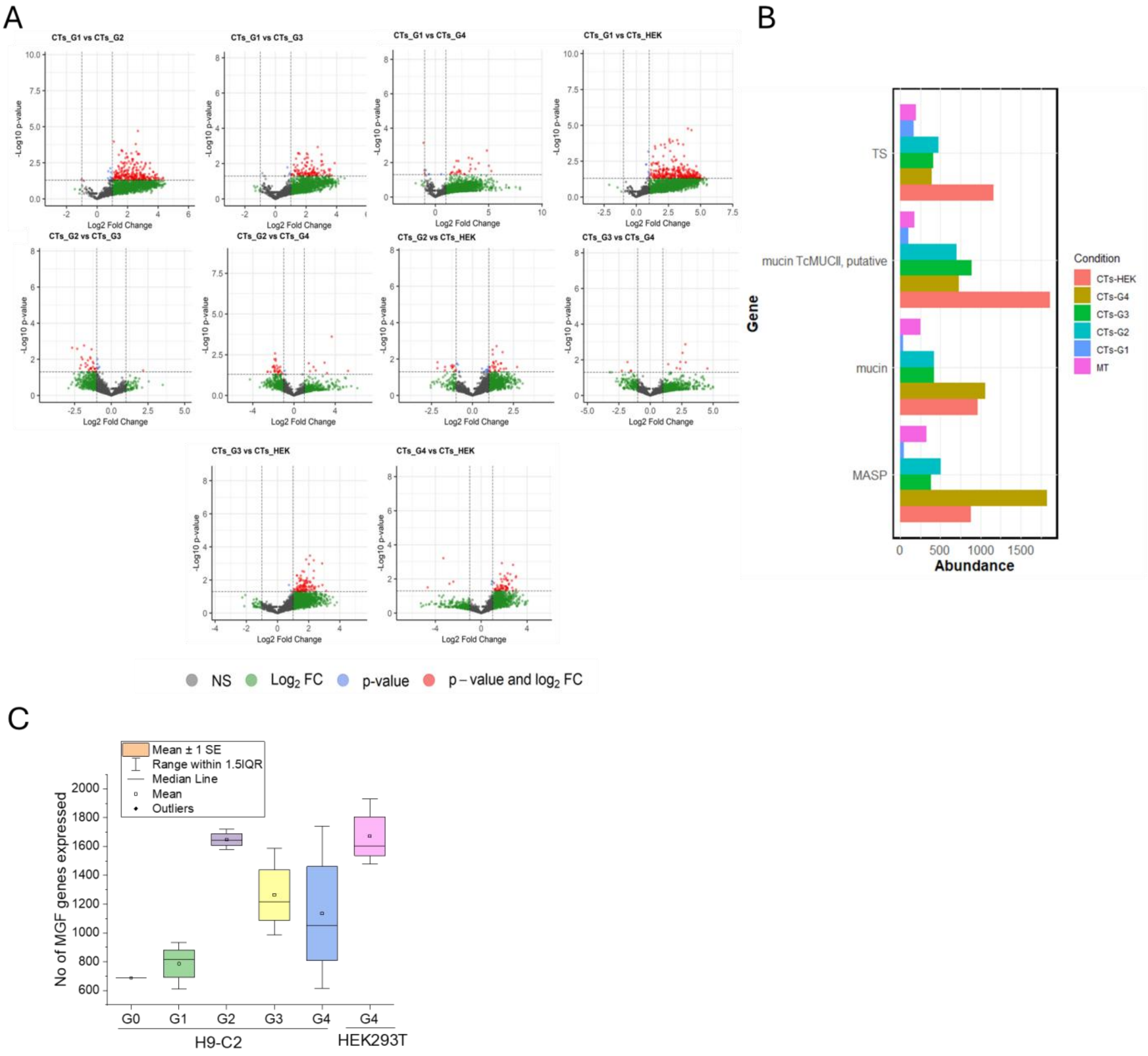

Supplementary Figure 15. Sequence alignment of the N-terminus of 30 TSs recognized by Chagas disease patients' antibodies. Alignment of the amino acids 30-100 of 30 TS proteins reveals significant sequence divergence and a few conserved amino acids. Conserved amino acids are highlighted in colour. The first 30 amino acids were removed to eliminate signal peptides.

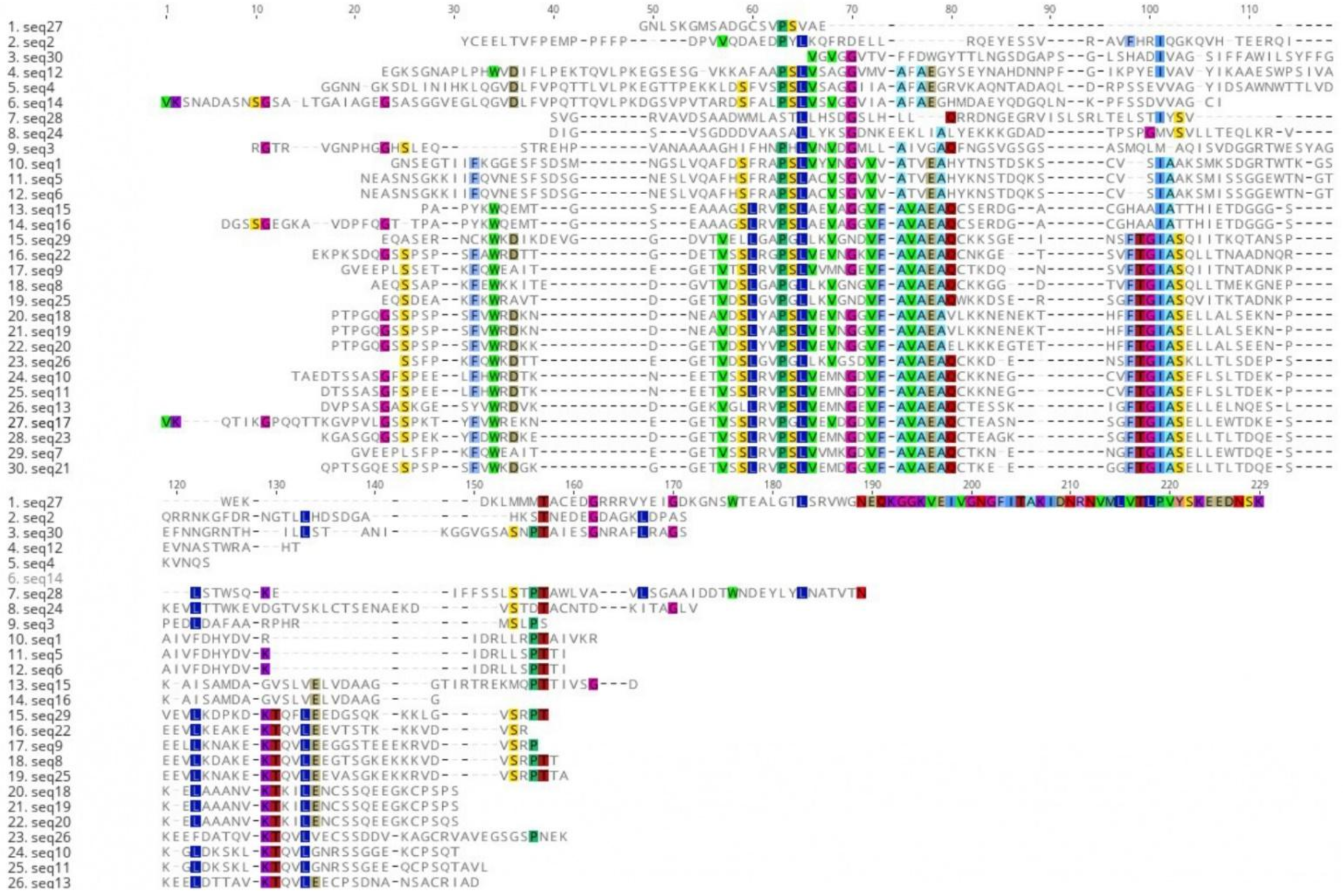

Supplementary Figure 16. Annotation pipeline used in the *T. cruzi* Sylvio X10 strain genome. The *T. cruzi* Sylvio X10 genome was annotated using GenSAS (Human et al. 2019, Methods Mol. Biol.), which integrates expressed sequence tags (ESTs), protein and nucleotide sequences, and RepeatMasker data. RNA-seq reads (Sequence Read Archive accession SRR9202394) were also utilized. Reads were mapped with HISAT2, and alignments were performed using BLASTn and BLAT. Structural annotation combined Augustus, PASA, GeneMarkES, and SNAP, with EvidenceModeler integrating predictions. Functional annotation was performed using BLASTp, DIAMOND, InterProScan, and Pfam, while SignalP and TargetP predicted protein features. TS subgroups were classified using BLAST against *T. cruzi* Dm28c strain. The final annotation was manually curated.

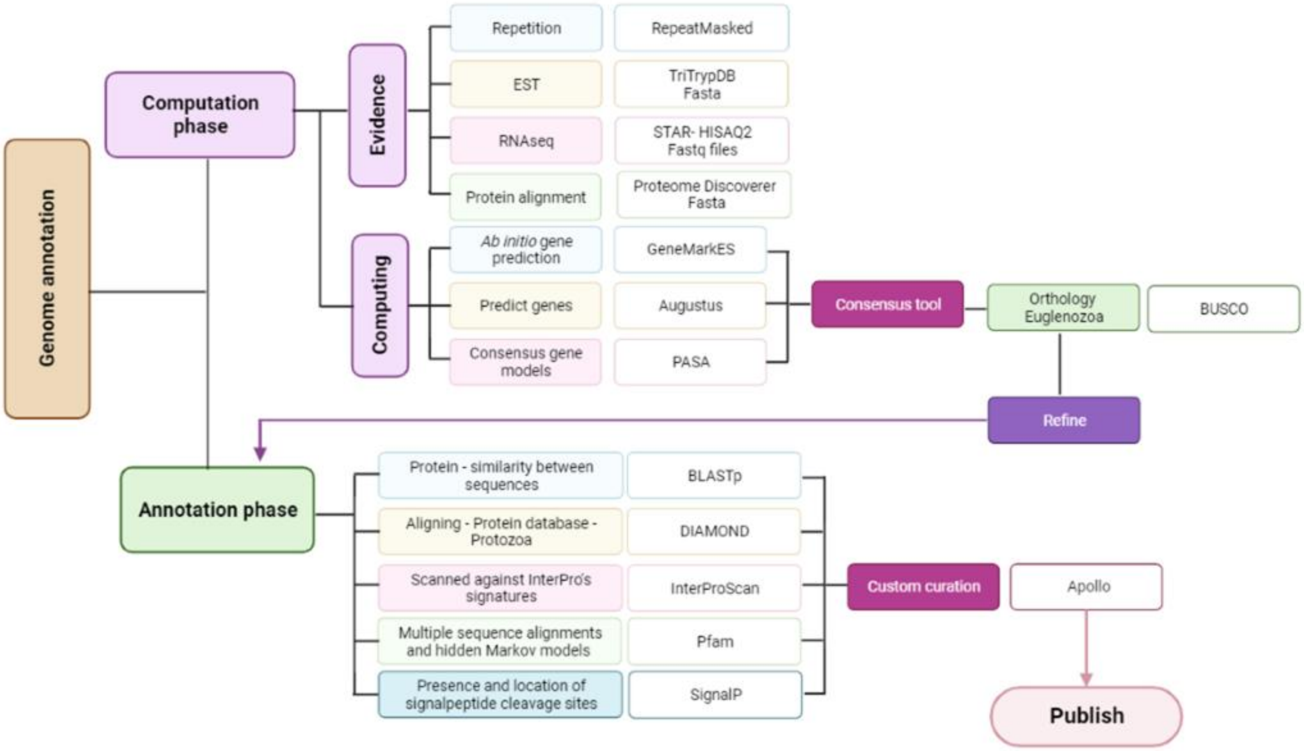

Supplementary Table 1. Synteny analysis between scaffolds and chromosomes. The data show the synteny relationship between scaffolds and chromosomes, including the number of syntenic blocks, total aligned length, total matched length, average alignment length, average percent identity, and average mapping quality.

Scaffolds ID	Chromosome ID	Number of Blocks	Total Aligned Length	Total Match Length	Average Alignment Length	Average Match Percent	Average Mapping Quality
2	TcSylvioHIFI_26	5	1429	1123	285.8	77.0	48.8
2	TcSylvioHIFI_8	17	151860	141539	8932.941176	89.3	28.1
3	TcSylvioHIFI_22	2	137262	35426	68631	25.8	5.0
4	TcSylvioHIFI_1	1	7449	4894	7449	65.7	60.0
4	TcSylvioHIFI_2	1	59996	57217	59996	95.4	60.0
4	TcSylvioHIFI_5	1	917	189	917	20.6	60.0
5	TcSylvioHIFI_30	1	60701	46634	60701	76.8	60.0
7	TcSylvioHIFI_26	1	51621	50126	51621	97.1	60.0
8	TcSylvioHIFI_9	1	38598	38520	38598	99.8	60.0
10	TcSylvioHIFI_25	1	34495	34198	34495	99.1	60.0
11	TcSylvioHIFI_16	1	34269	34048	34269	99.4	60.0
12	TcSylvioHIFI_30	1	33730	32391	33730	96.0	60.0
14	TcSylvioHIFI_30	1	25595	24690	25595	96.5	60.0
15	TcSylvioHIFI_5	1	25331	24662	25331	97.4	60.0
16	TcSylvioHIFI_8	10	116404	113287	11640.4	97.1	12.1
17	TcSylvioHIFI_11	1	10841	10834	10841	99.9	60.0
17	TcSylvioHIFI_3	1	12159	12149	12159	99.9	60.0
18	TcSylvioHIFI_28	3	33226	29331	11075.33333	88.3	5.3
18	TcSylvioHIFI_7	1	11396	11334	11396	99.5	60.0
19	TcSylvioHIFI_12	1	22085	20684	22085	93.7	60.0
20	TcSylvioHIFI_16	6	128862	117279	21477	91.0	0.2
23	TcSylvioHIFI_26	1	18568	17884	18568	96.3	60.0
24	TcSylvioHIFI_10	2	17468	17288	8734	98.0	60.0
29	TcSylvioHIFI_30	1	15848	14645	15848	92.4	60.0
30	TcSylvioHIFI_26	1	15737	15658	15737	99.5	60.0
32	TcSylvioHIFI_30	1	14350	14253	14350	99.3	60.0
34	TcSylvioHIFI_26	1	13342	13294	13342	99.6	60.0
35	TcSylvioHIFI_16	6	78888	74572	13148	94.5	0.5
36	TcSylvioHIFI_2	1	12081	11781	12081	97.5	60.0
37	TcSylvioHIFI_4	2	15837	15631	7918.5	97.9	30.0
38	TcSylvioHIFI_22	1	9802	2467	9802	25.2	39.0
39	TcSylvioHIFI_30	1	9538	9099	9538	95.4	60.0
40	TcSylvioHIFI_10	1	8680	8618	8680	99.3	60.0

Supplementary Table 2. Primers designed for nanopore multigene family sequencing. SL, splice leader sequence. Primers were designed to amplify all annotated MGF sequences.

Primer name	Sequence
SL _T. cruzi	AACGCTATTATTGATACAGTTTC
Actin_long	GACTGTTCTTCGTCAGACAT
β-Tubulin_long	GAGGAGGAGCAGTACTAG
α-Tubulin_long	GATGTGGAGGAGTACTAG
GAPDH_long	GTTCGGCAAGGTTGTAG
Mucin	CAGCCTCAGCAGCTCTG
Mucin-2	AAGCACCAACAGGGCG
TSI	AATGCCGAGGAGATCAAGACCTT
TSII	TTTCTGTACAACCGCCCACT
TSIII	ATGGCTCTAATTGGTGACAGCA
TSIV-V-VI	TTGGGACTGTGGGGGTTTG
TSVII-VIII	ATCCACGAGGTGCCGAA
TS unclassified	CGCGGAAGTAAACA
MASP	ACAGTGACGGCAGCACC
MASP-2	TGCAGCCACCCACTG
RHS	TTCGTACCTCCTCTAC
RHS_2	ACTCGCATTGTACACAG
DGF	TGCTGCGCGATGACGA
DGF-2	AACCGTGATGGCTCTG
GP63-1	GCAGTTTGACAGCTGCA
GP63-2	TCCGACCGCCGTCA
GP63-3	CGTCGGACCGGTATTC
GP63-4	CTGGACCACTGCTGCC
