# Supplementary material for: Variation in surface protein expression leads to heterogeneous Trypanosoma cruzi populations during host cell infection
Source: Nat Commun. 2025 Nov 12;16:9949. doi: 10.1038/s41467-025-64900-2 (PMC12612098; doi:10.1038/s41467-025-64900-2)
Supplement: Supplementary file 2 — Description of Additional Supplementary File [file 41467_2025_64900_MOESM2_ESM.pdf]

Description of Additional Supplementary Files

Supplementary data 1. Quantitative proteomics of *T. cruzi* life cycle stages.

Quantitative proteomics of *Trypanosoma cruzi* using tandem-mass tag mass spectrometry across four different life stages (EP, MT, AM, CT). The data includes Uniprot protein identification, protein description, protein abundance, protein abundance ratio comparing stages, experimental q-values combined, false discovery rates (FDR), combined protein FDR confidence, peptide coverage, number of identified peptides, sum posterior error probability (PEP) score, number of peptide-spectrum matches (PSMs), number of unique peptides, number of amino acids, protein molecular weight [kDa], and calculated isoelectric point (pI). Search engine metrics from Sequest High-Throughput (HT), including sequest score, number of protein pathway groups, and number of razor peptides, are also reported. EP, epimastigotes; MT, metacyclic trypomastigotes; AM, amastigotes; CTs, cell-derived trypomastigotes.

Supplementary data 2. Identification of MGF proteins that react with antibodies from Chagas disease patients using yeast surface display (YSD) of *T. cruzi* proteins. Enrichment analysis of the YSD antibody screen comparing MGF proteins enriched with antibodies from Chagas disease patients with those enriched with antibodies from healthy individuals. Chagas disease or healthy enriched YSD libraries were first compared to non-enriched libraries, and non-significant hits were removed. Only MGF proteins are shown. Statistical comparisons were performed with edgeR using a quasi-likelihood F-test (two-sided). Effect sizes are reported as log2 fold changes and log10 *p*-values. Significant hits are indicated considering a fold-change ≥ 2 and *p*-value ≤ 0.05.
