# Supplementary material for: Variation in surface protein expression leads to heterogeneous Trypanosoma cruzi populations during host cell infection
Source: Nat Commun. 2025 Nov 12;16:9949. doi: 10.1038/s41467-025-64900-2 (PMC12612098; doi:10.1038/s41467-025-64900-2)
Supplement: Supplementary file 5 — Reporting summary [file 41467_2025_64900_MOESM5_ESM.pdf]

nature portfolio

Corresponding author(s): Igor Cestari

Last updated by author(s): Sep 26, 2025

Reporting Summary

Nature Portfolio wishes to improve the reproducibility of the work that we publish. This form provides structure for consistency and transparency in reporting. For further information on Nature Portfolio policies, see our Editorial Policies and the Editorial Policy Checklist.

Statistics

For all statistical analyses, confirm that the following items are present in the figure legend, table legend, main text, or Methods section.

n/a	Confirmed	
☐	☒	The exact sample size (*n*) for each experimental group/condition, given as a discrete number and unit of measurement
☐	☒	A statement on whether measurements were taken from distinct samples or whether the same sample was measured repeatedly
☐	☒	The statistical test(s) used AND whether they are one- or two-sided<br>*Only common tests should be described solely by name; describe more complex techniques in the Methods section.*
☒	☐	A description of all covariates tested
☐	☒	A description of any assumptions or corrections, such as tests of normality and adjustment for multiple comparisons
☐	☒	A full description of the statistical parameters including central tendency (e.g. means) or other basic estimates (e.g. regression coefficient) AND variation (e.g. standard deviation) or associated estimates of uncertainty (e.g. confidence intervals)
☐	☒	For null hypothesis testing, the test statistic (e.g. *F*, *t*, *r*) with confidence intervals, effect sizes, degrees of freedom and *P* value noted<br>*Give P values as exact values whenever suitable.*
☒	☐	For Bayesian analysis, information on the choice of priors and Markov chain Monte Carlo settings
☒	☐	For hierarchical and complex designs, identification of the appropriate level for tests and full reporting of outcomes
☒	☐	Estimates of effect sizes (e.g. Cohen's *d*, Pearson's *r*), indicating how they were calculated

Our web collection on statistics for biologists contains articles on many of the points above.

Software and code

Policy information about availability of computer code

Data collection: Proteome Discoverer 2.2 (ThermoFisher Scientific) was used for mass spectrometry raw data collection and processing. MinKNOW 1.4.2 (Oxford Nanopore Technologies) was used for Oxford nanopore sequencing data collection and processing. BioTek Gen5 software 3.17.17 (BioTek) was used for Cytation 5 image collection and processing. The SMRT Link v25.3 software was used for PacBio HiFi DNA sequencing and data proccessing.

Data analysis	Genome assembly was performed using HiFiasm v0.19.5 for PacBio HiFi reads, followed by scaffolding with SALSA (available at https://github.com/cestari-lab/) using Pore-C data aligned with minimap2 v2.24. BED files were generated using BEDTools v2.30.0. Repeat content was evaluated using RepeatModeler v2.0.1 to build a de novo repeat library, and RepeatMasker v4.1.2 for annotation. Genome scaffolds were aligned and sorted using the AssemblyReferenceSorter tool in NGSEP v4.3.1. Genomic alignments were visualized in JBrowse2 and Circlize v0.4.16 in R v4.2.2. Average Nucleotide Identity (ANI) was calculated using FastANI v1.33.<br>Pore-C reads were mapped with minimap2, and DNA contact matrices were analyzed using pairtools v0.3.0. Chromosome ploidy analysis was conducted with samtools depth v0.1.18, following Schwabl et al.'s protocol.<br>Genome annotation was conducted using the GenSAS platform, incorporating alignments with HISAT2 v2.2.1, BLASTn v2.12.0+, and BLAT v2.5. Gene modeling used PASA v2.4.1, Augustus v3.4.0, GeneMarkES v4.48, and SNAP v2017-05-17. Annotation integration was performed using EvidenceModeler (EVM). Functional annotations employed BLASTp with BLOSUM62 matrix, DIAMOND v2.0.11, InterProScan v5.55-88.0, Pfam v34.0, SignalP v5.0, and TargetP v2.0.<br>Mass spectrometry raw data acquisition was carried out using Orbitrap Fusion (ThermoFisher Scientific) with DDA-MS3 mode and SPS-TMT settings. TMT data analysis was performed according to Thermo's default SPS-MS3 protocols (as in reference 72).<br>All custom scripts used for genome analysis and chromatin data processing are available at: https://github.com/cestari-lab. For coding citation, please refer to MGFseq (10.5281/zenodo.17137432), genome assembly (10.5281/zenodo.17137452), and other lab codes (10.5281/zenodo.17137473).

For manuscripts utilizing custom algorithms or software that are central to the research but not yet described in published literature, software must be made available to editors and reviewers. We strongly encourage code deposition in a community repository (e.g. GitHub). See the Nature Portfolio guidelines for submitting code & software for further information.

Data

Policy information about availability of data

All manuscripts must include a data availability statement. This statement should provide the following information, where applicable:

- Accession codes, unique identifiers, or web links for publicly available datasets
- A description of any restrictions on data availability
- For clinical datasets or third party data, please ensure that the statement adheres to our policy

The assembled genome of the T. cruzi Sylvio X10 strain is available in the National Center for Biotechnology Information (NCBI) with BioProject number PRJNA1237338 and at the DNA DataBank of Japan (DDBJ), the European Nucleotide Archive (ENA), and GenBank at NCBI under the accession JBMETK000000000 [https://www.ncbi.nlm.nih.gov/nuccore/JBMETK000000000.1/]. PacBio HiFi sequences and Pore-C data are available in the Sequence Read Archive (SRA) with the BioProject identification PRJNA1236874 [https://www.ncbi.nlm.nih.gov/bioproject/ PRJNA1236874/]. The mass spectrometry proteomics data have been deposited to the ProteomeXchange Consortium via the PRIDE partner repository with the dataset identifier PXD061891 [https://proteomecentral.proteomexchange.org/cgi/GetDataset?ID=PXD061891]. Codes used for data analysis are available at https://github.com/cestari-lab/. For coding citation, please refer to MGFseq (10.5281/zenodo.17137432), genome assembly (10.5281/zenodo.17137452), and other lab codes (10.5281/zenodo.17137473).

Research involving human participants, their data, or biological material

Policy information about studies with human participants or human data. See also policy information about sex, gender (identity/presentation), and sexual orientation and race, ethnicity and racism.

Reporting on sex and gender	n/a
Reporting on race, ethnicity, or other socially relevant groupings	n/a
Population characteristics	n/a
Recruitment	n/a
Ethics oversight	n/a

Note that full information on the approval of the study protocol must also be provided in the manuscript.

Field-specific reporting

Please select the one below that is the best fit for your research. If you are not sure, read the appropriate sections before making your selection.

☒ Life sciences ☐ Behavioural & social sciences ☐ Ecological, evolutionary & environmental sciences

For a reference copy of the document with all sections, see nature.com/documents/nr-reporting-summary-flat.pdf

Life sciences study design

All studies must disclose on these points even when the disclosure is negative.

Sample size	For genome sequencing, a single high-quality DNA preparation was obtained from approximately 3×10^7 parasites. Similarly, the Pore-C experiment was performed using material from 1.8×10^9 parasites. The TMT-based mass spectrometry included four biological replicates

	collected at different times for each of the four developmental stages of T. cruzi—epimastigotes (EP), metacyclic trypomastigotes (MT), amastigotes (AM), and cell-derived trypomastigotes (CT)—resulting in a total of 16 samples. Both the MGF-Seq analysis and the invasion assays were carried out using three biological replicates per generation of CT.
Data exclusions	No data were excluded from the experiments.
Replication	To ensure the reproducibility of the experimental findings, multiple biological replicates were used across experiments. In the TMT-based mass spectrometry analysis, four biological replicates were collected at different times for each developmental stage of T. cruzi, totaling 16 samples. The consistency of these replicates is demonstrated in Figure 2, where a principal component analysis (PCA) and hierarchical clustering heatmaps confirm that the replicates group tightly by stage, reflecting stage-specific proteomic profiles. For the MGF-Seq analysis and the invasion assays, three biological replicates were used per stage. In the invasion assay, the low dispersion of replicates is evident in the boxplots shown in Figure 3E. Similarly, in the MGF-Seq data, the heatmap in Figure 4B highlights the high similarity in gene expression among biological replicates, supporting the robustness of the transcriptomic profiles. Together, these visual and quantitative confirmations demonstrate that the experimental approaches produced reproducible and reliable results.
Randomization	n/a
Blinding	n/a

Reporting for specific materials, systems and methods

We require information from authors about some types of materials, experimental systems and methods used in many studies. Here, indicate whether each material, system or method listed is relevant to your study. If you are not sure if a list item applies to your research, read the appropriate section before selecting a response.

Materials & experimental systems

n/a	Involved in the study	
☒	☐	Antibodies
☐	☒	Eukaryotic cell lines
☒	☐	Palaeontology and archaeology
☒	☐	Animals and other organisms
☒	☐	Clinical data
☒	☐	Dual use research of concern
☒	☐	Plants

Methods

n/a	Involved in the study	
☒	☐	ChIP-seq
☒	☐	Flow cytometry
☒	☐	MRI-based neuroimaging

Eukaryotic cell lines

Policy information about cell lines and Sex and Gender in Research

Cell line source(s)	The following cell lines were obtained from ATCC (American Type Culture Collection):<br>1. H9C2 (ATCC CRL-1446) – Derived from rat heart tissue (cardiomyocytes).<br>2. HEK293-T (ATCC CRL-3216) – Derived from human embryonic kidney cells.<br>3. NIH/3T3 (ATCC CRL-1658) – Derived from mouse embryonic fibroblast tissue.<br>4. HeLa (ATCC CRM-CCL-2) – Derived from human cervical carcinoma tissue.<br>5. Trypanosoma cruzi strain Sylvio X10 (ATCC 50823) – A protozoan parasite isolated from a mammalian host.<br><br>These cell lines were purchased from ATCC, a well-known source for authenticated cell lines and microbial cultures used in research.
Authentication	The cells were authenticated according to ATCC's standard procedures.
Mycoplasma contamination	The cells were tested according from ATCC's standard procedures.
Commonly misidentified lines (See ICLAC register)	n/a

Plants

Seed stocks	n/a
Novel plant genotypes	n/a
Authentication	n/a
